# Clinical Benefit, Price, and Uptake for Cancer Biosimilars vs Reference Drugs in China: A Systematic Review and Meta-Analysis

**DOI:** 10.1001/jamanetworkopen.2023.37348

**Published:** 2023-10-12

**Authors:** Xingxian Luo, Xin Du, Zhuangqi Li, Jingwen Liu, Xufeng Lv, Haoran Li, Qixiang Guo, Cen Wang, Xuecai Xue, Kaidi Le, Xiaomeng Jiang, Lin Huang, Yue Yang

**Affiliations:** 1 School of Pharmaceutical Sciences, Tsinghua University, Beijing, China; 2 Key Laboratory of Innovative Drug Research and Evaluation, National Medical Products Administration, Beijing, China; 3 Tsinghua-Peking Center for Life Sciences, Beijing, China; 4 Center for Drug Evaluation, National Medical Products Administration, Beijing, China; 5 School of Life Sciences, Fudan University, Shanghai, China; 6 Department of Pharmacy, Peking University People’s Hospital, Beijing, China; 7 Department of Pharmacy, Cancer Institute & Hospital, Chinese Academy of Medical Sciences, Beijing, China

## Abstract

**Importance:**

The high cost of biologics used to treat cancer has been an increasing burden in the world. In China, the recent approval of cancer biosimilar drugs to resolve this problem is promising, but evidence of clinical benefits, price, and uptake for these drugs is still lacking.

**Objectives:**

To compare characteristics of pivotal clinical trials in China and other countries for biosimilars of bevacizumab, rituximab, and trastuzumab and investigate the efficacy or effectiveness, safety, and immunogenicity outcomes of cancer biosimilars compared with reference drugs by meta-analysis.

**Data Sources:**

For this systematic review and meta-analysis, PubMed, Embase, the Cochrane Library, and ClinicalTrials.gov were searched for published studies from database inception to February 1, 2023, using the search topics (*cancers*) AND (*biosimilars*).

**Study Selection:**

Randomized clinical trials and cohort studies that included patients with cancer were included.

**Data Extraction and Synthesis:**

Two authors independently extracted the outcome estimates and characteristics for each study. A random-effects meta-analysis was performed to summarize the relative estimates with 95% CIs. This study was performed following the Preferred Reporting Items for Systematic Reviews and Meta-analyses guideline.

**Main Outcomes and Measures:**

Clinical trial characteristics were collected for biosimilars of bevacizumab, rituximab, and trastuzumab. The relative estimates of efficacy or effectiveness (objective response rate, progression-free survival, and overall survival), safety, and immunogenicity outcomes were analyzed for biosimilars vs reference drugs. The weighted average price and uptake rate were evaluated for biosimilars relative to their reference drugs between 2015 and 2022.

**Results:**

A total of 39 RCTs (involving 18 791 patients) and 10 cohort studies (involving 1998 patients) were included. The biosimilars of bevacizumab (16 RCTs; risk ratio [RR], 0.97; 95% CI, 0.93-1.01; *P* = .17), rituximab (12 RCTs; RR, 1.03; 95% CI, 0.98-1.08; *P* = .70), and trastuzumab (9 RCTs: RR, 1.04; 95% CI, 0.97-1.12; *P* = .29) met equivalence with reference biologics in regard to the objective response rate. The results summarized from cohort studies were consistent with those from RCTs. In 2022, cancer biosimilars were priced at 69% to 90% of the costs for the reference drugs, and their uptake reached 54% to 83% in China.

**Conclusions and Relevance:**

This systematic review and meta-analysis indicated that cancer biosimilars provided comparable clinical benefits at lower prices compared with reference drugs. These findings suggest the potential feasibility of expediting the transition from reference drugs to biosimilars to benefit more patients with cancer.

## Key Points

**Question:**

What are the clinical benefits, price, and uptake rates of approved cancer biosimilars compared with their reference drugs in China and other countries?

**Findings:**

In this systematic review and meta-analysis of 39 randomized clinical trials and 10 cohort studies, clinical benefits of biosimilars of rituximab, bevacizumab, and trastuzumab were comparable to those of their reference drugs. In China in 2022, the biosimilar price was 69% to 90% of the reference drug cost, while uptake rates were 54% to 83%.

**Meaning:**

This study found equivalent clinical outcomes and lower prices of cancer biosimilars vs reference drugs, suggesting that initiatives to increase uptake of biosimilars in China could benefit more patients.

## Teaser

This systematic review and meta-analysis examines clinical benefits, costs, and uptake rates of approved cancer biosimilars of bevacizumab, rituximab, and trastuzumab compared with their corresponding reference drugs in China vs other countries.

## Introduction

Biological agents are increasingly used in clinical practice for the treatment of cancer, immune-mediated inflammatory disorders, and infectious diseases, with substantial effects.^1,2,3^ In 2019, biologics accounted for 27% of all novel cancer drugs approved by the European Medicines Agency (EMA), the US Food and Drug Administration (FDA), and the Japan Pharmaceuticals and Medical Devices Agency (PMDA).^4^ However, the high prices of biologics impose a serious burden on limited health care expenditures even for high-income countries.^5^

To address the high expense of novel biologics, the EMA established the first internationally approved pathway for biosimilars and authorized the first biosimilar, somatropin, in 2006.^6^ Similarly, the US established the Biologics Price Competition and Innovation Act of 2009 to abbreviate the regulatory pathway for biosimilars.^7^ In promoting the development of biosimilars, the PMDA issued the Guideline for the Quality, Safety, and Efficacy Assurance of Follow-on Biologics in 2009.^8^ As of December 2022, the EMA,^9^ FDA,^10^ and PMDA^11^ had approved more than 90, 40, and 32 biosimilars, respectively. Several studies have shown that biosimilars are equivalent to reference drugs, with varying degrees of price reduction.^7,12,13,14,15,16^ However, it was reported that biosimilars are still confronted with serious challenges to their use, including patent litigation, exclusivity protection, anticompetitive behavior, and interchangeability requirements, which have limited the use of biosimilars^4^ (eTable 1 in Supplement 1).

China has approved more than 200 new molecular entities since 2015, substantially improving patient accessibility.^17,18,19,20^ Of these, therapeutic biologics account for about one-third of all novel drugs. Like other low- and middle-income countries, China is also confronted with high costs for these novel biologic products despite their superior clinical benefits.^21^ Therefore, the Chinese government has placed a high priority on the development of biosimilars to improve affordability (eFigure 1 in Supplement 1). In 2015, China’s National Medical Products Administration (NMPA) issued technical guidelines for the development and evaluation of biosimilars to facilitate their development.^22^ Subsequently, the NMPA also issued guidelines for the development of individual biosimilars, such as rituximab,^23^ trastuzumab,^24^ and bevacizumab^25^ (eTable 2 in Supplement 1). These policies provided substantial incentives for research and development of biosimilars. As of February 2023, China had approved more than 20 biosimilars developed by local companies,^26^ trailing behind only the US, the European Union, and Japan; these approvals are expected to markedly increase accessibility of biosimilars for Chinese patients (eTables 3 and 4 in Supplement 1).

The high morbidity and mortality rates of cancer in China have resulted in a substantial economic burden. The biosimilars of bevacizumab, rituximab, and trastuzumab approved in China are anticipated to alleviate the financial burden on patients with cancer. To our knowledge, there are 37 cancer biosimilars under development in China (eTable 5 in Supplement 1). As the number of cancer biosimilar approvals increases, their clinical benefit (efficacy or effectiveness, safety, and immunogenicity), price, and uptake are rarely reported in China. Therefore, this study conducted a systematic review and meta-analysis of randomized clinical trials (RCTs) and cohort studies comparing the clinical benefit between cancer biosimilars and their respective reference drugs. Furthermore, the price and the uptake rate of biosimilars were also estimated relative to the reference drugs. This study may provide robust evidence for expanding the use of cancer biosimilars in China and elsewhere.

## Methods

### Data Sources

This systematic review and meta-analysis followed the related portions of the Preferred Reporting Items for Systematic Reviews and Meta-analyses (PRISMA) reporting guideline. The approvals for cancer biosimilars and their pivotal trials (defined as a phase 3 clinical trial of a biosimilar vs a reference drug) through December 2022 were extracted using the publicly available listing databases of the China NMPA,^26^ FDA,^10^ EMA,^9^ and PMDA.^11^ The prices and uptake of biosimilars were collected from a centralized procurement platform and the Pharnexcloud database module.^27^

### Eligibility and Exclusion Criteria

We included RCTs and cohort studies that included patients with cancer. The intervention groups were patients receiving any of the rituximab, trastuzumab, or bevacizumab biosimilars. The comparators were patients receiving the respective reference drugs. We did not have any restrictions on dosage, lines of therapy, treatment regimen, or number of patients included. Noncomparative studies (eg, reviews, expert commentaries, editorials, and clinical guidelines) were excluded. A detailed description of the eligibility and exclusion criteria is shown in eAppendix 1 in Supplement 1.

### Evidence Sources, Search Strategy, and Selection Process

We conducted a systematic search in Embase, PubMed, the Cochrane Library, and ClinicalTrials.gov from database inception to February 1, 2023, using the search topics (*cancers*) AND (*biosimilars*). The detailed evidence sources and search strategy are described in eAppendix 2 in Supplement 1. In addition, we manually searched publicly available review reports of cancer biosimilars from the NMPA,^26^ FDA,^10^ EMA,^9^ and PMDA^11^ and extracted their information on pivotal clinical trials. If the same study was published in multiple journals or posted in other sources (eg, ClinicalTrials.gov and review reports), we chose the report with the most informative data. Since some of the studies did not have publicly available full-text information, we extracted data based on abstracts. No restrictions were placed on the language. Two of us (X.L., X.D.) were responsible for screening documents independently for double-blind studies that met the inclusion criteria by title, abstract, and full text. If there was an inconsistency, it was resolved by a third reviewer (Z.L.).

### Data Extraction

Two of us (X.L., X.D.) extracted the characteristics of cancer biosimilars from RCTs and cohort studies, including the number of patients, whether the study was sponsored by a manufacturer, study design, duration of study, lines of therapy, efficacy or effectiveness end points, safety, and immunogenicity. The efficacy end points included objective response rate (ORR), progression-free survival (PFS), and overall survival (OS). The safety outcomes included adverse events of grade 3 or greater according to the Common Terminology Criteria for Adverse Events (version 4.0),^28^ serious adverse events, death, neutropenia (grade ≥3), and thrombocytopenia (grade ≥3). Additionally, immunogenicity outcomes included incidence of neutralizing antibodies and antidrug antibodies during treatment. Prices and quarterly sale volumes of each cancer biosimilar and reference drug were extracted for 2015 to 2022 (a detailed description is shown in eAppendix 3 in Supplement 1).

### Assessment of Risk of Bias

Two investigators (X.L., X.D.) assessed the risk of bias in the RCTs according to the Cochrane Collaboration’s tool,^29^ including selection bias (sequence generation and allocation concealment), performance bias (blinding of participants, personnel, and outcome assessors), attrition bias (incomplete outcome data), reporting bias (selective outcome reporting), and other bias. In addition, the Newcastle-Ottawa Scale was used to assess the risk of bias for the cohort studies.^30^

### Statistical Analysis

Medians (IQRs) were used for continuous variables, and counts and percentages were used for categorical variables. We determined the median of weighted average prices (WAPs) for drugs that had several approved biosimilars. For biosimilars or reference drugs with varying dose strengths, we unified the conversion to price per milligram (eg, trastuzumab is available in 440 mg per vial or 600 mg per vial in China) to better compare the price difference between them. Since the sales volume obtained was quarterly, the uptake rate of biosimilars was defined as the ratio between quarterly (3-month) sales volume of the biosimilar and total quarterly sales volume (quarterly sales volume of the biosimilar plus its reference product). The χ^2^ test was used to compare the uptake rate of biosimilars for 3 cancer drugs (rituximab, bevacizumab, and trastuzumab) with the uptake rates of the respective reference drugs in the first and second years after the entry of biosimilars into the market.

We pooled the relative estimates (eg, relative risk and hazard ratio) of rituximab, bevacizumab, and trastuzumab biosimilars separately, as the indications and mechanisms of the tested drugs were substantially different. Also, the pooled relative estimates were calculated separately for the RCTs and cohort studies due to substantial differences in their designs. The efficacy or effectiveness, safety, and immunogenicity outcomes of cancer biosimilars were all pooled using meta-analysis with a random-effects model, similar to a previous study.^7^ Heterogeneity across studies was assessed using the Cochrane *Q* statistic and quantified by *I*^2^ values.^31^ Subgroup analyses, including the type of indication, country of the sponsors, and sample size, were performed to assess the source of heterogeneity. To estimate potential publication biases, funnel plots, Begg tests, and Egger tests were conducted for primary efficacy end points. Relative estimates and 95% CIs were calculated based on the available data (number of cases and noncases by treatment group) if they were not reported in the trials. Sensitivity analyses were conducted for the primary efficacy end points by removing each study.

Statistical analyses were performed and graphical representations generated using IBM SPSS, version 20 (IBM Corp) and R, version 4.1.0 (R Project for Statistical Computing). The R packages used in the analysis included meta (version 5.2.0), forestplot (version 1.10.1), and ggplot2 (version 3.4.0). Two-sided tests were conducted with a significance threshold of *P* < .05.

## Results

### Search Results

eFigure 2 in Supplement 1 shows the process of study screening. Of the 1195 records sourced from the database and manually searched, 49 studies met the inclusion criteria, including 39 RCTs^32,33,34,35,36,37,38,39,40,41,42,43,44,45,46,47,48,49,50,51,52,53,54,55,56,57,58,59,60,61,62,63,64,65,66,67,68,69,70^ and 10 cohort studies.^71,72,73,74,75,76,77,78,79,80^

### RCT and Cohort Study Characteristics

Table 1 and eTable 6 in Supplement 1 summarize the characteristics of the 39 RCTs, which included 18 791 patients with cancer.^32,33,34,35,36,37,38,39,40,41,42,43,44,45,46,47,48,49,50,51,52,53,54,55,56,57,58,59,60,61,62,63,64,65,66,67,68,69,70^ Of those, 37 RCTs were published in journals,^32,33,34,35,37,38,39,40,41,42,43,44,45,46,47,48,49,50,52,53,54,55,56,57,58,59,60,61,62,63,64,65,66,67,68,69,70^ 1 was reported in ClinicalTrials.gov,^51^ and 1 was reported in an NMPA review.^36^ Of the 39 RCTs, 18 studied bevacizumab biosimilars,^32,33,34,35,36,37,38,39,40,41,42,43,44,45,46,47,48,49^ 12 studied rituximab biosimilars,^50,51,52,53,54,55,56,57,58,59,60,61^ and 9 studied trastuzumab biosimilars.^62,63,64,65,66,67,68,69,70^

**Table 1.** Characteristics of the Included RCTs and Cohort Studies

**Source**	**Country**	**Cancer type**	**Biosimilar drug**	**Reference drug**	**Patients, No.** ^a^	**Females, No./males, No.**	**Primary end point**	**Study design**
**Bevacizumab biosimilar vs bevacizumab**								
Stroyakovskiy et al,^34^ 2022	Russia	NSCLC	BCD-021	Bevacizumab	357	110/230	ORR	RCT
Thatcher et al,^48^ 2019	UK	NSCLC	ABP215	Bevacizumab	642	NA	ORR	RCT
Reinmuth et al,^49^ 2019	US	NSCLC	PF-06439535	Bevacizumab	719	252/467	ORR	RCT
Reck et al,^46^ 2020	Korea	NSCLC	SB8	Bevacizumab	763	255/508	ORR	RCT
Yang et al,^47^ 2019	China	NSCLC	IBI305	Bevacizumab	450	162/279	ORR	RCT
Trukhin et al,^39^ 2021	Ukraine	NSCLC	MB02	Bevacizumab	627	244/383	ORR	RCT
Rezvani et al,^45^ 2020	Iran	MCRC	BE1040V	Bevacizumab	126	35/91	PFS	RCT
Qin et al,^43^ 2021	China	MCRC	HLX04	Bevacizumab	677	271/404	PFS	RCT
Shi et al,^42^ 2021	China	NSCLC	LY01008	Bevacizumab	649	237/352	ORR	RCT
Chu et al,^44^ 2021	China	NSCLC	QL1101	Bevacizumab	535	217/318	ORR	RCT
Hengrui (sponsor),^36^ 2022	China	NSCLC	BP102	Bevacizumab	517	NA	ORR	RCT
Wan et al,^38^ 2021	China	NSCLC	MIL60	Bevacizumab	517	183/325	ORR	RCT
Syrigos et al,^40^ 2021	UK	NSCLC	FKB238	Bevacizumab	731	248/483	ORR	RCT
Kim et al,^35^ 2021	US	NSCLC	BI695502	Bevacizumab	671	246/417	ORR	RCT
Socinski et al,^41^ 2021	US	NSCLC	MYL-14020	Bevacizumab	671	247/424	ORR	RCT
Chen et al,^37^ 2022	China	NSCLC	BAT1706	Bevacizumab	651	NA	ORR	RCT
Lu et al,^32^ 2023	China	NSCLC	Table 008	Bevacizumab	549	194/457	ORR	RCT
Verschraegen et al,^33^ 2022	US	NSCLC	CT-P16	Bevacizumab	689	149/397	ORR	RCT
Zhao et al,^71^ 2023	China	DLBCL	QL1101	Bevacizumab	946	413/533	ORR	Cohort study
**Rituximab biosimilar vs rituximab**								
Kaplanov et al,^61^ 2014	Russia	FL	BCD-020	Rituximab	92	NA	ORR	RCT
Toogeh et al,^57^ 2018	Iran	CLL	Zytux	Rituximab	70	13/57	ORR	RCT
Jurczak et al,^60^ 2017	Poland	FL	GP2013	Rituximab	629	350/277	ORR	RCT
Kim et al,^59^ 2017	Korea	FL	CT-P10	Rituximab	140	77/63	ORR	RCT
Ogura et al,^58^ 2018	Japan	FL	CT-P10	Rituximab	258	145/123	ORR	RCT
Poddubnaya et al,^54^ 2020	Russia	Indolent NHL	BCD-020	Rituximab	174	90/84	ORR	RCT
Candelaria et al,^56^ 2019	Mexico	DLBCL	RTXM83	Rituximab	272	117/155	ORR	RCT
Sharman et al,^53^ 2020	US	FL	PF-05280586	Rituximab	394	216/178	ORR	RCT
Niederwieser et al,^55^ 2020	Germany	FL	ABP 798	Rituximab	256	126/130	ORR	RCT
Archigen (sponsor),^51^ 2020	Korea	FL	SAIT101	Rituximab	315	174/141	ORR	RCT
Shi et al,^52^ 2020	China	DLBCL	HLX01	Rituximab	407	182/220	ORR	RCT
Song et al,^50^ 2021	China	DLBCL	IBI301	Rituximab	419	202/214	ORR	RCT
Roy et al,^74^ 2013	India	DLBCL	Reditux	Rituximab	173	51/112	NA	Cohort study
Bankar et al,^73^ 2020	India	DLBCL	Reditux	Rituximab	152	41/111	NA	Cohort study
Deng et al,^72^ 2022	China	DLBCL	HLX-01	Rituximab	33	13/20	NA	Cohort study
**Trastuzumab biosimilar vs trastuzumab**								
Stebbing et al,^69^ 2017	UK	Early BC	CT-P6	Trastuzumab	549	549/0	PCR	RCT
Rugo et al,^70^ 2017	US	MBC	MYL-14010	Trastuzumab	500	500/0	ORR	RCT
von Minckwitz et al,^67^ 2018	Germany	Early BC	ABP 980	Trastuzumab	725	725/0	PCR	RCT
Pivot et al,^68^ 2018	France	Early BC	SB3	Trastuzumab	875	875/0	PCR	RCT
Pegram et al,^66^ 2019	US	MBC	PF-05280014	Trastuzumab	707	707/0	ORR	RCT
Pivot et al,^62^ 2022	France	Early BC	HD201	Trastuzumab	502	502/0	PCR	RCT
Alexeev et al,^65^ 2020	Russia	MBC	BCD-022	Trastuzumab	225	225/0	ORR	RCT
Xu et al,^64^ 2021	China	MBC	HLX02	Trastuzumab	649	649/0	ORR	RCT
Nodehi et al,^63^ 2022	Iran	Early BC	TA4415V	Trastuzumab	92	92/0	PCR	RCT
Bae et al,^80^ 2021	Korea	Early and metastatic BC	CT-P6	Trastuzumab	254	NA	PCR and PFS	Cohort study
Park et al,^77^ 2022	Korea	Advanced GC	CT-P6	Trastuzumab	102	14/88	NA	Cohort study
Yang et al,^76^ 2022	Canada	Early BC	MYL-1401O	Trastuzumab	136	NA	PCR	Cohort study
Bernat-Peguera et al,^79^ 2022	Spain	Early BC	CT-P6	Trastuzumab	44	NA	NA	Cohort study
Eser et al,^75^ 2023	Turkey	Early and metastatic BC	MYL-1401O	Trastuzumab	53	NA	PCR and PFS	Cohort study
Liu et al,^78^ 2022	China	Early BC	HLX02	Trastuzumab	105	105/0	NA	Cohort study

Abbreviations: BC, breast cancer; CLL, chronic lymphocytic leukemia; DLBCL, diffuse large B-cell lymphoma; FL, follicular lymphoma; GC, gastric cancer; MBC, metastatic breast cancer; MCRC, metastatic colorectal cancer; NA, not available; NHL, non-Hodgkin lymphoma; NSCLC, non–small cell lung cancer; ORR, objective response rate; PCR, pathologic complete remission; PFS, progression-free survival; RCT, randomized clinical trial.

^a^ The number of patients was defined as the number of patients at randomization. The combined number of males and females of some studies does not equal the patient total because data on sex were missing for some patients.

Of the RCTs, 33 were prespecified as equivalence designs,^32,33,34,35,36,37,38,39,40,41,42,43,44,46,47,48,49,50,51,52,53,54,55,58,60,62,64,65,66,67,68,69,70^ while 6 were noninferiority designs^45,56,57,59,61,63^ (eTable 6 in Supplement 1). More than half of the RCTs (23 [59.0%]) were multiregional clinical trials.^34,35,37,39,40,41,46,48,49,51,53,54,55,56,60,61,62,64,65,66,67,69,70^ The median number of patients enrolled in the RCTs was 646 (IQR, 522-676) for bevacizumab biosimilars, 265 (IQR, 166-397) for rituximab biosimilars, and 549 (IQR, 500-707) for trastuzumab biosimilars. The median duration for assessing the primary efficacy end points was 18 weeks (IQR, 18-21 weeks) for bevacizumab biosimilars, 23 weeks (IQR, 23-24 weeks) for rituximab biosimilars, and 24 weeks (IQR, 23-24 weeks) for trastuzumab biosimilars. Thirty-four RCTs reported the incidence of antidrug antibodies,^32,33,34,35,36,37,38,39,40,41,42,43,45,46,47,48,49,50,51,52,53,54,55,56,58,59,60,62,63,64,66,67,68,70^ and 28 reported the incidence of neutralizing antibodies.^32,33,34,35,36,37,38,39,40,41,42,43,47,48,49,50,51,52,53,54,55,58,62,63,64,65,66,67^

Table 1 and eTable 7 in Supplement 1 summarize the characteristics of the 10 cohort studies with a total of 1998 patients.^71,72,73,74,75,76,77,78,79,80^ Of the 10 cohort studies, 1 (10.0%) was of a bevacizumab biosimilar,^71^ 3 (30.0%) were of rituximab biosimilars,^72,73,74^ and 6 (60.0%) were of trastuzumab biosimilars.^75,76,77,78,79,80^ All cohort study designs were retrospective.

The risk of bias assessment in RCTs and cohort studies is shown in eTables 8 and 9, respectively, in Supplement 1. Of the RCTs, 32 (82.1%) were low risk,^32,33,34,35,36,38,39,40,42,43,45,46,47,48,49,50,51,53,54,55,57,58,59,60,63,64,65,66,67,68,69,70^ 5 (12.8%) were uncertain risk,^37,41,44,52,62^ and 2 (5.1%) were high risk.^56,61^ Of the cohort studies, 4 (40.0%) were high risk^72,76,77,79^ and 6 (60.0%) were low risk.^71,73,74,75,78,80^

### Comparison With Pivotal Trials Among the 4 Agencies

As of February 2023, the FDA had approved 12 cancer biosimilars; the EMA, 16; the NMPA, 11; and the PMDA, 9 (Table 2 and eTable 10 in Supplement 1). The majority of biosimilars approved by the 4 regulatory agencies were evaluated in equivalence trial designs with low risk of bias (45 of 47 [95.7%]). In regard to the cancer types, RCTs of bevacizumab biosimilars enrolled primarily patients with non–small cell lung cancer (NSCLC). For rituximab biosimilars, the EMA, FDA, and PMDA included patients with follicular lymphoma, whereas the NMPA included patients with diffuse large B-cell lymphoma (DLBCL). Patients with early and metastatic breast cancer were included in pivotal clinical trials for biosimilars of trastuzumab (Table 2). Table 3 summarizes a pooled analysis of the primary efficacy end points in pivotal clinical trials of cancer biosimilars approved by the 4 regulatory agencies. With the exception of an EMA-approved rituximab biosimilar for patients with follicular lymphoma, the findings revealed no significant difference in primary efficacy end points between biosimilars and reference drugs.

**Table 2.** Comparison of Pivotal Clinical Trials Used to Support Approval of Cancer Biosimilars by the FDA, EMA, PMDA, and NMPA

Characteristic	FDA	EMA	PMDA	NMPA
**Bevacizumab biosimilar vs bevacizumab**				
Products, No.	4	7	4	8
Pivotal trials, No.	4	7	4	8
Sample size, median (IQR), No.	666 (638-696)	689 (657-725)	666 (638-696)	542 (517-650)
Equivalence, No.	4/4	7/7	4/4	8/8
Cancer type				
NSCLC, No./total No.	4/4	7/7	4/4	7/8
MCRC, No./total No.	0/4	0/7	0/4	1/8
**Rituximab biosimilar vs rituximab**				
Products, No.	3	3	2	2
Pivotal trials, No.	3	3	2	2
Sample size, median (IQR), No.	258 (257-326)	256 (198-325)	512 (452-570)	413 (410-416)
Equivalence, No.	2/3	2/3	2/2	2/2
Cancer type				
FL, No./total No.	3/3	3/3	2/2	0/2
DLBCL, No./total No.	0/3	0/3	0/2	2/2
**Trastuzumab biosimilar vs trastuzumab**				
Products, No.	5	6	3	1
Pivotal trials, No.	5	6	3	1
Sample size, median (IQR), No.	707 (549-725)	678 (574-721)	707 (628-716)	649 (649-649)
Equivalence, No.	5/5	6/6	3/3	1/1
Cancer type				
Early BC, No./total No.	3/5	3/6	2/3	0/1
MBC, No./total No.	2/5	3/6	1/3	1/1

Abbreviations: BC, breast cancer; DLBCL, diffuse large B-cell lymphoma; EMA, European Medicines Agency; FDA, US Food and Drug Administration; FL, follicular lymphoma; MBC, metastatic breast cancer; MCRC, metastatic colorectal cancer; NMPA, National Medical Products Administration; NSCLC, non–small cell lung cancer; PMDA, Japanese Pharmaceuticals and Medical Devices Agency.

**Table 3.** Summary Results of Primary Efficacy End Points Used to Support Pivotal Trials for FDA, EMA, PMDA, and NMPA Approval of Cancer Biosimilars

Outcome and cancer subgroup	RCTs, No.	Sample size, No.		Test of heterogeneity		RR (95% CI)^a^	*P* value
		Biosimilar	Reference drug	*I*^2^, %	*P* value		
**Bevacizumab biosimilar vs bevacizumab**							
NSCLC ORR							
FDA	4	1343	1334	0	.78	0.97 (0.89-1.06)	.48
EMA	7	2423	2419	0	.65	0.99 (0.93-1.05)	.64
PMDA	4	1343	1334	0	.76	0.97 (0.89-1.06)	.48
NMPA	7	1938	1930	0	.60	0.99 (0.93-1.05)	.63
CRC PFS							
NMPA	1	340	337	NA	NA	0.92 (0.78-1.06)	.27
**Rituximab biosimilar vs rituximab**							
FL ORR							
FDA	3	552	524	0	.66	1.06 (0.99-1.14)	.12
EMA	3	462	466	0	.70	1.06 (1.00-1.13)	.049
PMDA	2	510	513	17	.27	1.01 (0.95-1.08)	.69
DLBCL							
NMPA	2	410	416	52	.15	0.99 (0.93-1.04)	.65
**Trastuzumab biosimilar vs trastuzumab**							
Early BC PCR							
FDA	3	1072	1077	68	.04	1.11 (0.94-1.32)	.21
EMA	3	1072	1077	68	.04	1.11 (0.94-1.32)	.21
PMDA	2	635	639	74	.05	1.05 (0.83-1.34)	.68
MBC ORR							
FDA	2	602	605	64	.09	1.01 (0.87-1.16)	.93
EMA	3	926	930	28	.25	1.00 (0.93-1.08)	.99
PMDA	1	352	355	NA	NA	0.94 (0.84-1.05)	.27
NMPA	1	324	325	NA	NA	1.00 (0.91-1.11)	.98

Abbreviations: BC, breast cancer; CRC, colorectal cancer; DLBCL, diffuse large B-cell lymphoma; EMA, European Medicines Agency; FDA, US Food and Drug Administration; FL, follicular lymphoma; MBC, metastatic breast cancer; NA, not available; NMPA, National Medical Products Administration; NSCLC, non–small cell lung cancer; ORR, objective response rate; PCR, pathologic complete remission; PFS, progression-free survival; PMDA, Japan Pharmaceuticals and Medical Devices Agency; RCT, randomized clinical trial; RR, risk ratio.

^a^ Results of meta-analysis.

### Clinical Benefit of Bevacizumab Biosimilars vs Bevacizumab

Meta-analysis of 16 RCTs^32,33,34,35,36,37,38,39,40,41,42,44,46,47,48,49^ showed that the primary efficacy end point (ORR) of bevacizumab biosimilars was comparable to that of the reference drugs in the treatment of NSCLC (risk ratio [RR], 0.97; 95% CI, 0.93-1.01; *P* = .17) (eFigure 3 in Supplement 1). No significant differences in the primary efficacy end point of PFS rate (RR, 0.92; 95% CI, 0.78-1.07; *P* = .27) or PFS (HR, 0.79; 95% CI, 0.46-1.35; *P* = .47) between bevacizumab biosimilars and the reference drugs were observed among patients with metastatic colorectal cancer. Subgroup analysis showed no significant differences in ORR between bevacizumab biosimilars and reference drugs across disease setting, country of sponsors, and sample size (eTable 11 in Supplement 1). Overall survival and PFS were not significantly different between biosimilars and their reference drugs. No significant differences were found in safety and immunogenicity outcomes between bevacizumab biosimilars and reference drugs (eTable 12 in Supplement 1).

A study from a clinical setting showed that the ORR of bevacizumab biosimilars was also comparable to that of the reference drug in patients with NSCLC.^76^ No significant difference in safety outcomes was observed between bevacizumab biosimilars and reference groups (eTable 13 in Supplement 1).

### Clinical Benefit of Rituximab Biosimilars vs Rituximab

The meta-analysis of 12 RCTs^50,51,52,53,54,55,56,57,58,59,60,61^ revealed no significant difference in the primary efficacy end point (ORR) between rituximab biosimilars and reference drugs in the treatment of lymphoma (RR, 1.03; 95% CI, 0.98-1.08; *P* = .70) (eFigure 4 in Supplement 1). Also, subgroup analyses indicated a comparable ORR between biosimilars and reference drugs (eTable 11 in Supplement 1). No significant difference was found in secondary efficacy end points (OS and PFS), safety outcomes, and immunogenicity outcomes between rituximab biosimilars and the reference drugs (eTable 12 in Supplement 1).

Three cohort studies of rituximab biosimilars were reported for the treatment of DLBCL.^72,73,74^ The pooled ORR results showed comparable effectiveness of rituximab biosimilars and originator drugs. The occurrence of grade 3 or higher AEs, neutropenia (grade ≥3), and thrombocytopenia (grade ≥3) did not differ significantly between the rituximab biosimilar group and the reference groups (eTable 13 in Supplement 1).

### Clinical Benefit of Trastuzumab Biosimilars vs Trastuzumab

The meta-analysis of 9 RCTs^62,63,64,65,66,67,68,69,70^ found no difference in the primary efficacy end points (ORR and pathologic remission) between trastuzumab biosimilars and reference drugs in the treatment of breast cancer (RR, 1.04; 95% CI, 0.97-1.12; *P* = .29) (eFigure 5 in Supplement 1). Subgroup analysis also suggested comparable efficacy outcomes between trastuzumab biosimilars and reference drugs (eTable 11 in Supplement 1). No significant differences were observed in secondary efficacy end points (OS and PFS), safety outcomes, and immunogenicity outcomes between trastuzumab biosimilars and the reference drugs (eTable 12 in Supplement 1).

Five cohort studies of trastuzumab biosimilars were for breast cancer,^75,76,78,79,80^ while 1 was for gastric cancer.^77^ There were no significant differences in effectiveness and safety outcomes between the rituximab biosimilar group and the reference group (eTable 13 in Supplement 1).

### Sensitivity Analysis and Publication Bias

Sensitivity analyses showed no significant change in the results of the primary efficacy end points after deleting each of the studies for the biosimilars of bevacizumab (eFigure 6 in Supplement 1), rituximab (eFigure 7 in Supplement 1), and trastuzumab (eFigure 8 in Supplement 1). No significant bias was observed in the primary efficacy end points for bevacizumab biosimilars (eFigure 9 in Supplement 1), rituximab biosimilars (eFigure 10 in Supplement 1), and trastuzumab biosimilars (eFigure 11 in Supplement 1), as suggested by the results of the Egger test, Begg test, and funnel plot.

### Price and Uptake of Cancer Biosimilars vs Reference Drugs

**Figure 1.**
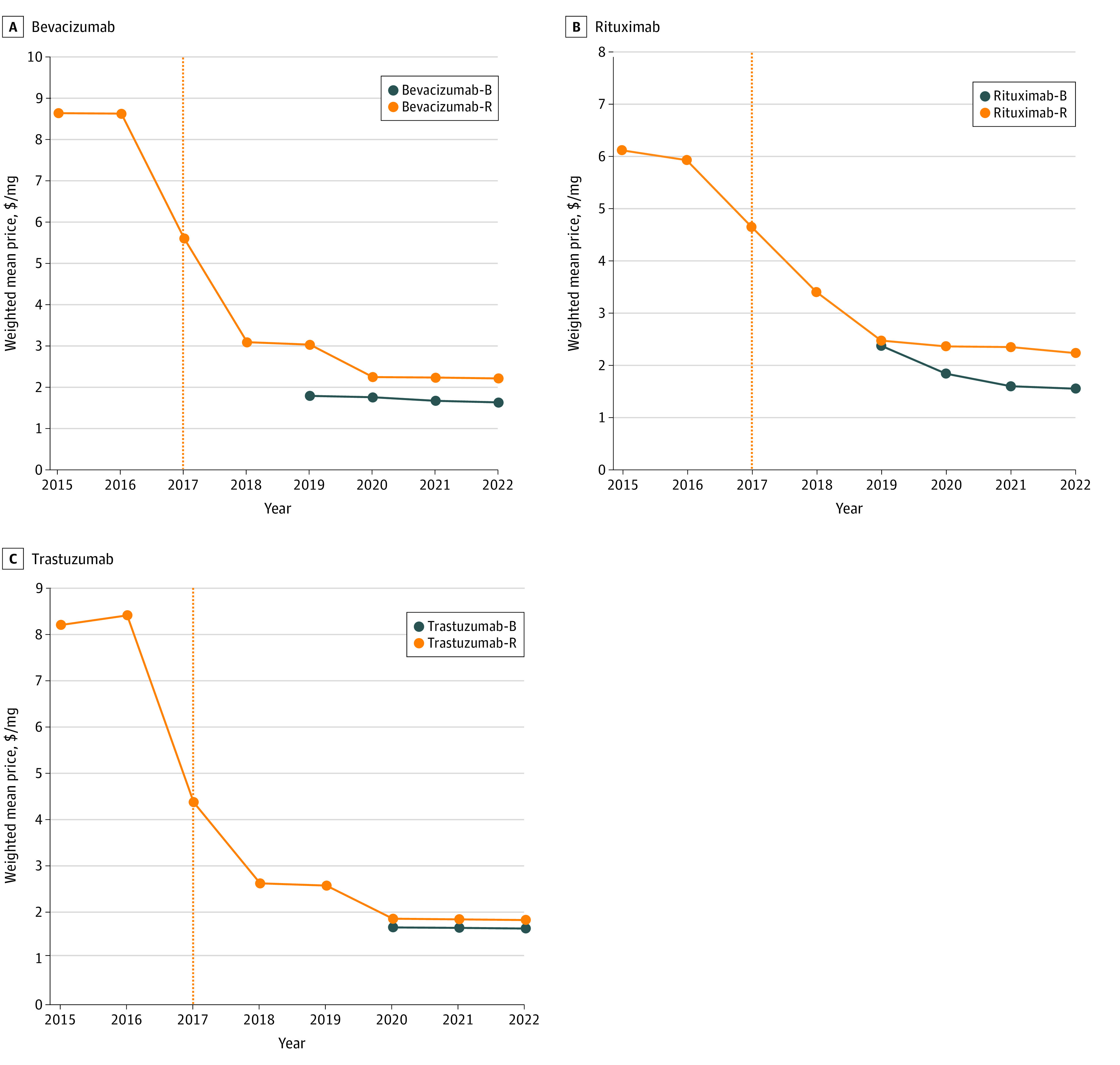
Trends in Weighted Average Price (WAP) per Unit for Rituximab, Bevacizumab, and Trastuzumab Biosimilars Compared With Reference Drugs Between 2015 and 2022 Orange dotted lines represent the year bevacizumab, rituximab, and trastuzumab were included on the National Reimbursement Drug List. B indicates biosimilar; R, reference.

**Figure 2.**
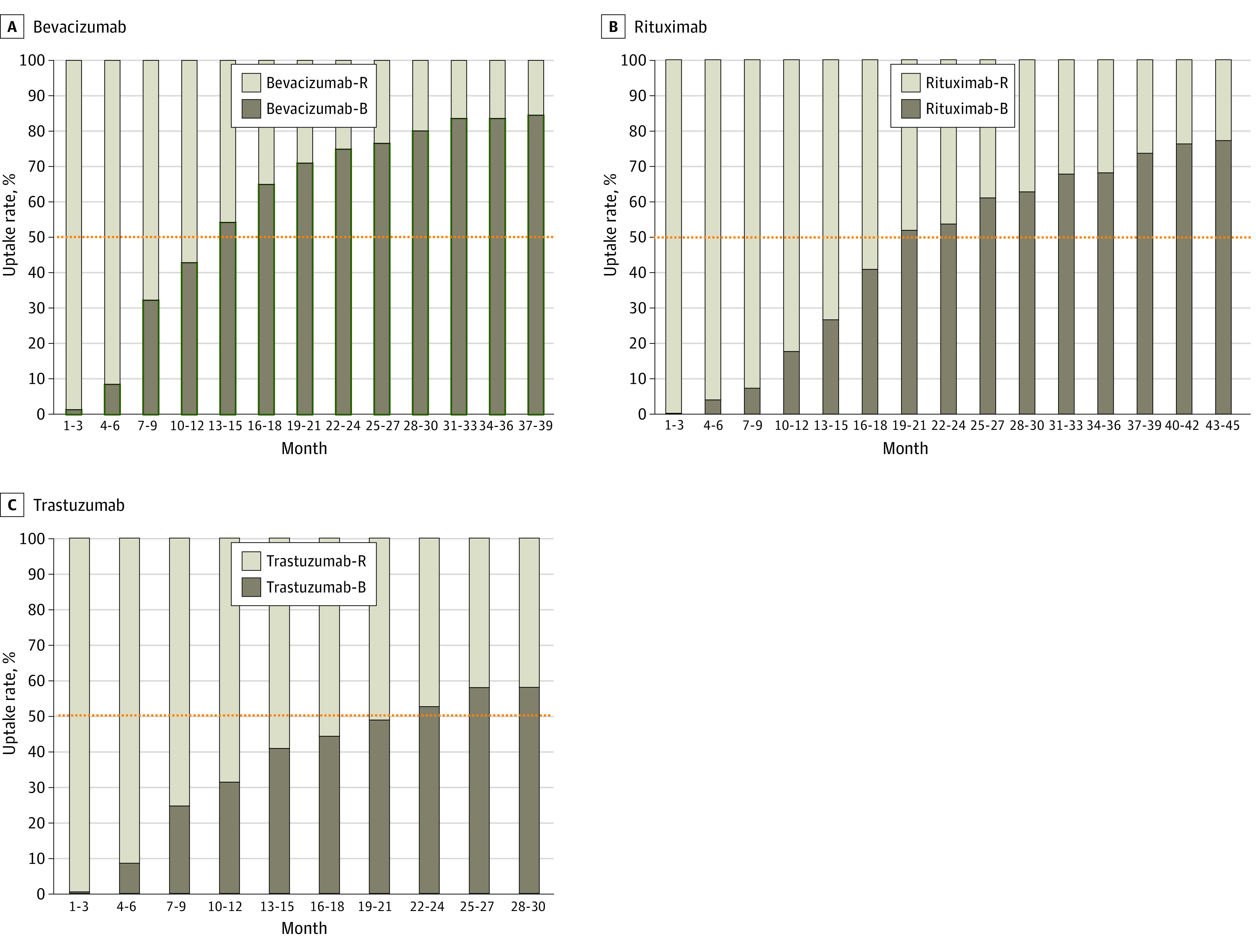
Trends in the Uptake Rate of Rituximab, Bevacizumab, and Trastuzumab Biosimilars vs Reference Drugs After Market Entry The x-axis represents the time since the biosimilar was introduced to the market. The y-axis represents the uptake rates of biosimilars vs their reference drugs per quarter. Bevacizumab biosimilars and rituximab biosimilars were launched for sale in 2019, whereas trastuzumab biosimilar was launched for sale in 2020. The cutoff date for price uptake rates of biosimilars was December 31, 2022. The annual uptake rate for biosimilars is calculated by dividing the 1-year sales of biosimilars by the 1-year sales of their reference drugs. Orange dotted lines represent a 50% uptake rate of biosimilars vs reference drugs. B indicates biosimilar; R, reference.

The median WAP of cancer biosimilars vs reference drugs from 2015 to 2022 is shown in Figure 1 and eTable 14 in Supplement 1. Prices of cancer biosimilars showed a significant decline after 2017. In 2022, the estimated median WAP was 74% of the reference drug for bevacizumab biosimilars, 69% for rituximab biosimilars, and 90% for trastuzumab biosimilars. The uptake rate of cancer biosimilars showed an increasing trend over time (Figure 2 and eTable 15 in Supplement 1). eTable 16 in Supplement 1 shows the uptake rates of biosimilars for the 3 cancer drugs in the first and second year after launch and at the latest time (2022). The uptake rates of biosimilars for the 3 cancer drugs were significantly different in the first and second year after market entry. Bevacizumab biosimilars had the highest uptake rates in the first and second years after launch (26% and 68%, respectively), followed by trastuzumab biosimilars (20% and 47%) and rituximab biosimilars (6% and 44%). In 2022, the uptake rates for bevacizumab, rituximab, and trastuzumab biosimilars were 83%, 74%, and 54%, respectively.

## Discussion

China initiated its biosimilar development at a later stage in comparison to the European Union, the US, and Japan. Nevertheless, with the initiation of China’s drug review reform in 2015, the research and development of biosimilars has garnered significant interest among domestic Chinese enterprises. The results showed that locally developed cancer biosimilars approved by the NMPA were comparable to those of the FDA, EMA, and PMDA in terms of the quantity and quality of clinical trial designs. All cancer biosimilars authorized by China were required to have a double-anonymized, equivalence, and low risk-of-bias design. Additionally, our study suggested that cancer biosimilars approved in China were shown to be equivalent to reference drugs in efficacy end points, safety outcomes, and immunogenicity outcomes. These findings indicated that China established rigorous and globally consistent review standards for cancer biosimilars.

The pooled analysis of 39 RCTs and 10 cohort studies revealed that bevacizumab biosimilars, rituximab biosimilars, and trastuzumab biosimilars did not differ significantly from the reference drugs with respect to efficacy, safety, and immunogenicity outcomes. Previous studies^7,13^ also showed that the biosimilars of bevacizumab, rituximab, and trastuzumab had equally rigorous clinical trial designs and similar efficacy as the reference drugs, which was consistent with our findings. However, in most cases, studies included in our meta-analysis were exclusively premarket pivotal RCTs, which typically had more stringent patient inclusion and exclusion criteria, potentially differing to some extent from patients being treated in a clinical setting.^81^ Therefore, this study included cohort studies and suggested that the effectiveness and safety outcomes of these cancer biosimilars did not differ noticeably from those of the reference medications. These results from routine clinical practice add credence to the view that cancer biosimilars and reference medicines are clinically equivalent.

The price of cancer biosimilars is a worldwide issue since it directly affects affordability for patients and public health expenditures.^4^ Typically, biosimilars are priced between 70% and 85% of the reference product, while generics can be priced at only 20% of the originators.^82,83^ Our results showed varying degrees of price reductions for cancer biosimilars compared with their reference drugs in China. In 2022, rituximab biosimilars accounted for the lowest price relative to the reference drugs (69%), followed by bevacizumab (74%) and trastuzumab (90%) biosimilars, similar to other countries.^4^ On a worldwide scale, biosimilars for the treatment of cancer are typically discounted by 30% in Europe, 10% to 33% in the US, and 30% in Japan.^4^ In the current circumstances, the reimbursement policy for biosimilars and their reference drugs is identical in China^84^ (eTable 17 in Supplement 1). Interestingly, in 2022, trastuzumab biosimilars had a lesser price drop than the reference drug (90% of the reference drug price) compared with rituximab biosimilars (69% of the reference drug price) and bevacizumab biosimilars (74% of the reference drug price), possibly because only trastuzumab biosimilars were approved in China.

Indication extrapolation policies for biosimilars can play a crucial role in ensuring that patients have access to medications. A previous study reported that biosimilars in the US and Japan were under pressure from considerable patent litigation.^4^ Our findings also suggested that the NMPA and EMA appeared to have less stringent extrapolation requirements than the US and Japan (eTable 18 in Supplement 1). To our knowledge, bevacizumab, trastuzumab, or rituximab are not currently granted regulatory exclusivity or a patent term extension in China, which contributes to some extent to the development of cancer biosimilars. A previous study by some of us reported that China was strengthening intellectual property protection for novel drugs, including the establishment of patent term extension and regulatory exclusivity.^17^ Hence, it is anticipated that China will strive to achieve a harmonious balance in promoting research on biosimilars and reference medications in the forthcoming years.

The rapid shift from biologic reference drugs to biosimilars has been a worldwide challenge.^4,85,86^ The uptake of cancer biosimilars also varies significantly between countries. A previous study showed that Denmark reached a 90% uptake rate in trastuzumab biosimilar entry after 3 months, while the lowest rate any country had was less than 10%.^87^ In the US, a slow market uptake of cancer biosimilars was reported.^88,89^ In China, bevacizumab biosimilars had the highest rate of uptake in the second year of marketing (68%), followed by trastuzumab biosimilars (47%) and rituximab biosimilars (44%). It should be admitted that the shift to cancer biosimilars in China has been relatively slow compared with Denmark. The success of the Danish model was largely attributed to its automated substitution system and the joint coordination among clinicians, administrators, patient organizations, and drug suppliers.^90^ This strategy was similar to China’s national volume-based procurement (NVBP) for generic pharmaceuticals, which has also yielded favorable outcomes.^91^ Therefore, accelerating the uptake of cancer biosimilars through NVBP and multistakeholder consensus should be considered in China. Additionally, the uptake rate of biosimilars can be influenced by the preferences of both patients and clinicians.^92^ Further enhancement of understanding of biosimilars by physicians and patients is needed in China.

### Limitations

There are some limitations to this study. First, due to the unavailability of comprehensive publications for some of the trials, our analysis was solely based on abstracts, thereby restricting our full understanding of the investigations, similar to a previous study.^7^ Second, this study did not conduct research on influencing patient and physician preferences for biosimilars, which would be more conducive to expansion of biosimilar use in China. Furthermore, this study rarely retrieved cancer biosimilars with quality problems or rejection of biologics license applications, likely because this information was not readily available to the public.^93^ Finally, this study focused on a pooled analysis of the primary efficacy end point. Other secondary efficacy end points, such as duration of response, were not analyzed due to the limited information available.

## Conclusions

This systematic review and meta-analysis found that the clinical benefits of cancer biosimilars were comparable to those of the reference biologics according to the evidence of the included RCTs and cohort studies. Furthermore, these biosimilars were considerably less expensive, which could have substantial implications for expanding access to treatment for patients with cancer. Our findings suggest that China should speed up its efforts to promote the use of biosimilars to benefit more patients with cancer.

## Notes

Supplement 1. **eAppendix 1.** Detailed Description of the Eligibility and Exclusion Criteria **eAppendix 2.** Evidence Sources and Search Strategy **eAppendix 3.** Price and Uptake of Biosimilar and Reference Drugs **eTable 1.** Comparison of Regulatory Factors for Biosimilar Oncology Drugs in the NMPA, FDA, EMA, and PMDA **eTable 2.** Individual Biosimilar Guidelines Issued by the NMPA in China **eTable 3.** Cancer Biosimilars Approved by the FDA, EMA, PMDA, and NMPA as of February 1, 2023 **eTable 4.** Biosimilar Products Approved in China **eTable 5.** Approved and In-Development Oncology Biosimilar Products in China as of February 1, 2023 **eTable 6.** Demographic and Clinical Characteristics of the Included RCTs **eTable 7.** Demographic and Clinical Characteristics of the Included Cohort Studies **eTable 8.** Results of the Risk of Bias Assessment of the RCTs Based on the Cochrane Collaboration’s Tools **eTable 9.** Results of the Risk of Bias Assessment of the Cohort Studies Based on the Newcastle-Ottawa Risk of Bias Assessment Tool **eTable 10.** Characteristics of Pivotal Clinical Trials Used to Support Approval of Cancer Biosimilars by the NMPA, FDA, EMA, and PMDA **eTable 11.** Subgroup Analysis of Cancer Biosimilars Compared With Reference Drugs Derived From Randomized Clinical Trials **eTable 12.** Pooled Analysis of Efficacy, Safety, and Immunogenicity of Cancer Biosimilars Derived From Randomized Clinical Trials **eTable 13.** Pooled Analysis of Efficacy, Safety, and Immunogenicity of Cancer Biosimilars Derived From Cohort Studies **eTable 14.** Weighted Mean Price per Unit of Rituximab, Bevacizumab, Trastuzumab, and Their Biosimilars Between 2015 and 2022 **eTable 15.** Trends in the Uptake Rate of Rituximab, Bevacizumab, and Trastuzumab Biosimilars vs Reference Drugs After Market Entry as of December 2022 **eTable 16.** Uptake Rates of Cancer Biosimilars vs Reference Drugs in the First Year, Second Year, and Latest Time on the Market **eTable 17.** Reimbursable Cancer Indications for the Biosimilars and Reference Drugs of Bevacizumab, Rituximab, and Trastuzumab in the National Reimbursement Drug List in China as of July 1, 2023 **eTable 18.** Comparison of the Approved Indications of Cancer Biosimilars vs Reference Drugs by the NMPA, FDA, EMA, and PMDA as of July 2023 **eFigure 1.** Development History of Biosimilars in China **eFigure 2.** Flowchart of Included Studies for the Systematic Review and Meta-analysis **eFigure 3.** Meta-analysis of the Primary Efficacy End Points Between Bevacizumab Biosimilars and the Originator Derived from RCTs **eFigure 4.** Meta-analysis of the Primary Efficacy End Points Between Rituximab Biosimilars and the Originator Stratified by Cancer Type Derived From RCTs **eFigure 5.** Meta-analysis of the Primary Efficacy End Points Between Trastuzumab Biosimilars and the Originator Stratified by Cancer Type Derived From RCTs **eFigure 6.** Sensitivity Analysis of Primary Efficacy End Points for Bevacizumab Derived From RCTs **eFigure 7.** Sensitivity Analysis of Primary Efficacy End Points for Rituximab Derived From RCTs **eFigure 8.** Sensitivity Analysis of Primary Efficacy End Points for Trastuzumab Derived From RCTs **eFigure 9.** Publication Bias Assessment of Primary Efficacy End Points for Bevacizumab Derived From RCTs **eFigure 10.** Publication Bias Assessment of Primary Efficacy End Points for Rituximab Derived From RCTs **eFigure 11.** Publication Bias Assessment of Primary Efficacy End Points for Trastuzumab Derived From RCTs
eReferences

Supplement 2.
Data Sharing Statement

## References

[1] Singh S, Murad MH, Fumery M, . Comparative efficacy and safety of biologic therapies for moderate-to-severe Crohn’s disease: a systematic review and network meta-analysis. Lancet Gastroenterol Hepatol. 2021;6(12):1002-1014. doi:10.1016/S2468-1253(21)00312-5 34688373 PMC8933137

[2] Schrama D, Reisfeld RA, Becker JC. Antibody targeted drugs as cancer therapeutics. Nat Rev Drug Discov. 2006;5(2):147-159. doi:10.1038/nrd1957 16424916

[3] Angriman F, Ferreyro BL, Burry L, . Interleukin-6 receptor blockade in patients with COVID-19: placing clinical trials into context. Lancet Respir Med. 2021;9(6):655-664. doi:10.1016/S2213-2600(21)00139-9 33930329 PMC8078877

[4] Bennett CL, Schoen MW, Hoque S, . Improving oncology biosimilar launches in the EU, the USA, and Japan: an updated policy review from the Southern Network on Adverse Reactions. Lancet Oncol. 2020;21(12):e575-e588. doi:10.1016/S1470-2045(20)30485-X 33271114 PMC12491062

[5] Ioannidis JP, Karassa FB, Druyts E, Thorlund K, Mills EJ. Biologic agents in rheumatology: unmet issues after 200 trials and $200 billion sales. Nat Rev Rheumatol. 2013;9(11):665-673. doi:10.1038/nrrheum.2013.134 23999553

[6] Cohen HP, Hachaichi S, Bodenmueller W, Kvien TK, Danese S, Blauvelt A. Switching from one biosimilar to another biosimilar of the same reference biologic: a systematic review of studies. BioDrugs. 2022;36(5):625-637. doi:10.1007/s40259-022-00546-6 35881304 PMC9485085

[7] Bloomfield D, D’Andrea E, Nagar S, Kesselheim A. Characteristics of clinical trials evaluating biosimilars in the treatment of cancer: a systematic review and meta-analysis. JAMA Oncol. 2022;8(4):537-545. doi:10.1001/jamaoncol.2021.7230 35113135 PMC8814981

[8] Guideline for the quality, safety, and efficacy assurance of follow-on biologics. Japan Pharmaceuticals and Medical Devices Agency. March 4, 2009. Accessed September 11, 2023. https://www.pmda.go.jp/files/000153851.pdf33662290

[9] European Medicines Agency. European public assessment reports. Accessed September 11, 2023. https://www.ema.europa.eu/en/medicines/download-medicine-data#european-public-assessment-reports-(epar)-section

[10] US Food and Drug Administration. Biosimilars. Accessed September 11, 2023. https://www.fda.gov/drugs/therapeutic-biologics-applications-bla/biosimilars

[11] Pharmaceuticals and Medical Devices Agency. Review reports: drugs. Accessed September 11, 2023. https://www.pmda.go.jp/english/review-services/reviews/approved-information/drugs/0001.html

[12] Ascef BO, Almeida MO, Medeiros-Ribeiro AC, Oliveira de Andrade DC, Oliveira Junior HA, de Soárez PC. Therapeutic equivalence of biosimilar and reference biologic drugs in rheumatoid arthritis: a systematic review and meta-analysis. JAMA Netw Open. 2023;6(5):e2315872. doi:10.1001/jamanetworkopen.2023.15872 37234004 PMC10220520

[13] Yang J, Yu S, Yang Z, . Efficacy and safety of anti-cancer biosimilars compared to reference biologics in oncology: a systematic review and meta-analysis of randomized controlled trials. BioDrugs. 2019;33(4):357-371. doi:10.1007/s40259-019-00358-1 31175632

[14] Rémuzat C, Kapuśniak A, Caban A, . Supply-side and demand-side policies for biosimilars: an overview in 10 European member states. J Mark Access Health Policy. 2017;5(1):1307315. doi:10.1080/20016689.2017.1307315 28740617 PMC5508392

[15] Kvien TK, Patel K, Strand V. The cost savings of biosimilars can help increase patient access and lift the financial burden of health care systems. Semin Arthritis Rheum. 2022;52:151939. doi:10.1016/j.semarthrit.2021.11.009 35027243

[16] Xue W, Lloyd A, Falla E, Roeder C, Papsch R, Bühler K. A cost-effectiveness evaluation of the originator follitropin alpha compared to the biosimilars for assisted reproduction in Germany. Int J Womens Health. 2019;11:319-331. doi:10.2147/IJWH.S193048 31191040 PMC6524790

[17] Luo X, Yang L, Du X, Yang J, Qian F, Yang Y. Analysis of patent and regulatory exclusivity for novel agents in China and the United States: a cohort study of drugs approved between 2018 and 2021. Clin Pharmacol Ther. 2022;112(2):335-343. doi:10.1002/cpt.2625 35485980

[18] Luo X, Qian F, Yang L, Li Y, Yang Y. Assessment of the breakthrough-therapy-designated drugs granted in China: a pooled analysis 2020-2022. Drug Discov Today. 2022;27(12):103370. doi:10.1016/j.drudis.2022.103370 36154876

[19] Luo X, Du X, Li Z, Qian F, Yang Y. Assessment of the delay in novel anticancer drugs between China and the United States: a comparative study of drugs approved between 2010 and 2021. Clin Pharmacol Ther. 2023;113(1):170-181. doi:10.1002/cpt.2755 36151921

[20] Luo X, Du X, Huang L, . The price, efficacy, and safety of within-class targeted anticancer medicines between domestic and imported drugs in China: a comparative analysis. Lancet Reg Health West Pac. 2022;32:100670. doi:10.1016/j.lanwpc.2022.100670 36785854 PMC9918802

[21] Huang HY, Wu DW, Ma F, . Availability of anticancer biosimilars in 40 countries. Lancet Oncol. 2020;21(2):197-201. doi:10.1016/S1470-2045(19)30860-5 32007192

[22] Technical guidelines for development and evaluation of biosimilar drugs. China National Medical Products Administration. Accessed September 11, 2023. https://www.cde.org.cn/zdyz/domesticinfopage?zdyzIdCODE=f044cdf4b7d7286aa12ffb85fc81a74c

[23] Guidelines for clinical trials of rituximab injection biosimilars. China National Medical Products Administration. Accessed September 11, 2023. https://www.cde.org.cn/zdyz/domesticinfopage?zdyzIdCODE=1c8cbfee534239f519cf1b976041321c

[24] Guidelines for clinical trials of trastuzumab biosimilars for injection. China National Medical Products Administration. Accessed September 11, 2023. https://www.cde.org.cn/zdyz/domesticinfopage?zdyzIdCODE=aa6f11df97765a072f0d2de8b8e1ad16

[25] Clinical trial guidelines for bevacizumab injection biosimilars. China National Medical Products Administration. Accessed September 11, 2023. https://www.cde.org.cn/zdyz/domesticinfopage?zdyzIdCODE=b29a336524c3b8c8d49f34d94bb3b955

[26] The database of listed drugs. China National Medical Products Administration. Accessed September 11, 2023. https://www.cde.org.cn/main/xxgk/listpage/b40868b5e21c038a6aa8b4319d21b07d

[27] Pharnexcloud. National hospital sales volume. Accessed September 11, 2023. https://pharma.bcpmdata.com/

[28] Common terminology criteria for adverse events (CTCAE). Version 4.0. National Cancer Institute. Accessed September 11, 2023. https://ctep.cancer.gov/protocolDevelopment/electronic_applications/ctc.htm

[29] Higgins JP, Altman DG, Gøtzsche PC, ; Cochrane Bias Methods Group; Cochrane Statistical Methods Group. The Cochrane Collaboration’s tool for assessing risk of bias in randomised trials. BMJ. 2011;343:d5928. doi:10.1136/bmj.d5928 22008217 PMC3196245

[30] Vu T, Feih J, Juul J. Fluctuating voriconazole concentrations during extracorporeal membrane oxygenation. J Pharm Pract. 2023;36(4):998-1001. doi:10.1177/08971900211060959 35612553

[31] Ioannidis JP, Patsopoulos NA, Evangelou E. Uncertainty in heterogeneity estimates in meta-analyses. BMJ. 2007;335(7626):914-916. doi:10.1136/bmj.39343.408449.80 17974687 PMC2048840

[32] Lu S, Qin S, Zhou Z, . Bevacizumab biosimilar candidate TAB008 compared to Avastin in patients with locally advanced, metastatic EGFR wild-type non-squamous non-small cell lung cancer: a randomized, double-blind, multicenter study. J Cancer Res Clin Oncol. 2023;149(9):5907-5914. doi:10.1007/s00432-022-04563-4 36595042 PMC11796671

[33] Verschraegen C, Andric Z, Moiseenko F, . Candidate bevacizumab biosimilar CT-P16 versus European Union reference bevacizumab in patients with metastatic or recurrent non-small cell lung cancer: a randomized controlled trial. BioDrugs. 2022;36(6):749-760. doi:10.1007/s40259-022-00552-8 36169807 PMC9649513

[34] Stroyakovskiy DL, Fadeeva NV, Matrosova MP, . Randomized double-blind clinical trial comparing safety and efficacy of the biosimilar BCD-021 with reference bevacizumab. BMC Cancer. 2022;22(1):129. doi:10.1186/s12885-022-09243-7 35105329 PMC8808992

[35] Kim ES, Balser S, Rohr KB, Lohmann R, Liedert B, Schliephake D. Phase 3 trial of BI 695502 plus chemotherapy versus bevacizumab reference product plus chemotherapy in patients with advanced nonsquamous NSCLC. JTO Clin Res Rep. 2021;3(1):100248. doi:10.1016/j.jtocrr.2021.100248 34993498 PMC8713120

[36] review reports of bevacizumab biosimilar. China National Medical Products Administration. Accessed August 4, 2023. https://file1.dxycdn.com/2022/0922/070/7198507456499203753-117.pdf

[37] Chen L, Trukhin D, Kolesnik O, . Clinical efficacy and safety of the BAT1706 (proposed bevacizumab biosimilar) compared with reference bevacizumab in patients with advanced nonsquamous NSCLC: a randomized, double-blind, phase III study. J Clin Oncol. 2022;40(16)(suppl):9041. doi:10.1200/JCO.2022.40.16_suppl.9041

[38] Wan R, Dong X, Chen Q, . Efficacy and safety of MIL60 compared with bevacizumab in advanced or recurrent non-squamous non-small cell lung cancer: a phase 3 randomized, double-blind study. EClinicalMedicine. 2021;42:101187. doi:10.1016/j.eclinm.2021.101187 34841235 PMC8606331

[39] Trukhin D, Poddubskaya E, Andric Z, ; STELLA Investigators. Efficacy, safety and immunogenicity of MB02 (bevacizumab biosimilar) versus reference bevacizumab in advanced non-small cell lung cancer: a randomized, double-blind, phase III study (STELLA). BioDrugs. 2021;35(4):429-444. doi:10.1007/s40259-021-00483-w 33914256 PMC8295170

[40] Syrigos K, Abert I, Andric Z, ; AVANA Investigators. Efficacy and safety of bevacizumab biosimilar FKB238 versus originator bevacizumab: results from AVANA, a phase III trial in patients with non-squamous non-small-cell lung cancer (non-sq-NSCLC). BioDrugs. 2021;35(4):417-428. doi:10.1007/s40259-021-00489-4 34264503 PMC8295151

[41] Socinski MA, Waller CF, Idris T, . Phase III double-blind study comparing the efficacy and safety of proposed biosimilar MYL-1402O and reference bevacizumab in stage IV non-small-cell lung cancer. Ther Adv Med Oncol. 2021;13:17588359211045845. doi:10.1177/17588359211045845 34819997 PMC8606731

[42] Shi Y, Lei K, Jia Y, . Bevacizumab biosimilar LY01008 compared with bevacizumab (Avastin) as first-line treatment for Chinese patients with unresectable, metastatic, or recurrent non-squamous non-small-cell lung cancer: a multicenter, randomized, double-blinded, phase III trial. Cancer Commun (Lond). 2021;41(9):889-903. doi:10.1002/cac2.12179 34184418 PMC8441057

[43] Qin S, Li J, Bai Y, . Efficacy, safety, and immunogenicity of HLX04 versus reference bevacizumab in combination with XELOX or mFOLFOX6 as first-line treatment for metastatic colorectal cancer: results of a randomized, double-blind phase III study. BioDrugs. 2021;35(4):445-458. doi:10.1007/s40259-021-00484-9 PMC8295119 34014555

[44] Chu T, Lu J, Bi M, . Equivalent efficacy study of QL1101 and bevacizumab on untreated advanced non-squamous non-small cell lung cancer patients: a phase 3 randomized, double-blind clinical trial. Cancer Biol Med. 2021;18(3):816-824. doi:10.20892/j.issn.2095-3941.2020.0212 33710815 PMC8330542

[45] Rezvani H, Mortazavizadeh SM, Allahyari A, . Efficacy and safety of proposed bevacizumab biosimilar BE1040V in patients with metastatic colorectal cancer: a phase III, randomized, double-blind, noninferiority clinical trial. Clin Ther. 2020;42(5):848-859. doi:10.1016/j.clinthera.2020.03.009 32334845

[46] Reck M, Luft A, Bondarenko I, . A phase III, randomized, double-blind, multicenter study to compare the efficacy, safety, pharmacokinetics, and immunogenicity between SB8 (proposed bevacizumab biosimilar) and reference bevacizumab in patients with metastatic or recurrent nonsquamous non-small cell lung cancer. Lung Cancer. 2020;146:12-18. doi:10.1016/j.lungcan.2020.05.027 32502923

[47] Yang Y, Wu B, Huang L, . Biosimilar candidate IBI305 plus paclitaxel/carboplatin for the treatment of non-squamous non-small cell lung cancer. Transl Lung Cancer Res. 2019;8(6):989-999. doi:10.21037/tlcr.2019.12.23 32010577 PMC6976344

[48] Thatcher N, Goldschmidt JH, Thomas M, . Efficacy and Safety of the Biosimilar ABP 215 Compared with Bevacizumab in Patients With Advanced Nonsquamous Non-small Cell Lung Cancer (MAPLE): a randomized, double-blind, phase III study. Clin Cancer Res. 2019;25(7):2088-2095. doi:10.1158/1078-0432.CCR-18-2702 30617139

[49] Reinmuth N, Bryl M, Bondarenko I, . PF-06439535 (a bevacizumab biosimilar) compared with reference bevacizumab (Avastin), both plus paclitaxel and carboplatin, as first-line treatment for advanced non-squamous non-small-cell lung cancer: a randomized, double-blind study. BioDrugs. 2019;33(5):555-570. doi:10.1007/s40259-019-00363-4 31338773 PMC6790355

[50] Song Y, Zhou H, Zhang H, . Efficacy and safety of the biosimilar IBI301 plus standard CHOP (I-CHOP) in comparison with rituximab plus CHOP (R-CHOP) in patients with previously untreated diffuse large B-cell lymphoma (DLBCL): a randomized, double-blind, parallel-group, phase 3 trial. Adv Ther. 2021;38(4):1889-1903. doi:10.1007/s12325-020-01603-8 33751401

[51] A randomized, double-blind, multi-center, multi-national trial to evaluate the efficacy, safety, and immunogenicity of SAIT101 versus rituximab as a first-line immunotherapy treatment in patients with low tumor burden follicular lymphoma (RAMO-2). ClinicalTrials.gov identifier: NCT02809053. Updated October 8, 2020. Accessed September 11, 2023. https://www.clinicaltrials.gov/study/NCT02809053

[52] Shi Y, Song Y, Qin Y, . A phase 3 study of rituximab biosimilar HLX01 in patients with diffuse large B-cell lymphoma. J Hematol Oncol. 2020;13(1):38. doi:10.1186/s13045-020-00871-9 32299513 PMC7164184

[53] Sharman JP, Liberati AM, Ishizawa K, . A randomized, double-blind, efficacy and safety study of pf-05280586 (a rituximab biosimilar) compared with rituximab reference product (MabThera) in subjects with previously untreated CD20-positive, low-tumor-burden follicular lymphoma (LTB-FL). BioDrugs. 2020;34(2):171-181. doi:10.1007/s40259-019-00398-7 PMC7113218 31820339

[54] Poddubnaya IV, Alekseev SM, Kaplanov KD, . Proposed rituximab biosimilar BCD-020 versus reference rituximab for treatment of patients with indolent non-Hodgkin lymphomas: an international multicenter randomized trial. Hematol Oncol. 2020;38(1):67-73. doi:10.1002/hon.2693 31724191

[55] Niederwieser D, Hamm C, Cobb P, . Efficacy and safety of ABP 798: results from the JASMINE trial in patients with follicular lymphoma in comparison with rituximab reference product. Target Oncol. 2020;15(5):599-611. doi:10.1007/s11523-020-00748-4 33044684 PMC7568694

[56] Candelaria M, González DE, Delamain MT, ; RTXM83 study. Rituximab biosimilar RTXM83 versus reference rituximab in combination with CHOP as first-line treatment for diffuse large B-cell lymphoma: a randomized, double-blind study. Leuk Lymphoma. 2019;60(14):3375-3385. doi:10.1080/10428194.2019.1633632 31272251

[57] Toogeh G, Faranoush M, Razavi SM, . A double-blind, randomized comparison study between Zytux vs MabThera in treatment of CLL with FCR regimen: non-inferiority clinical trial. Int J Hematol Oncol Stem Cell Res. 2018;12(2):84-91. 30233768 PMC6141434

[58] Ogura M, Sancho JM, Cho SG, . Efficacy, pharmacokinetics, and safety of the biosimilar CT-P10 in comparison with rituximab in patients with previously untreated low-tumour-burden follicular lymphoma: a randomised, double-blind, parallel-group, phase 3 trial. Lancet Haematol. 2018;5(11):e543-e553. doi:10.1016/S2352-3026(18)30157-1 30389036

[59] Kim WS, Buske C, Ogura M, . Efficacy, pharmacokinetics, and safety of the biosimilar CT-P10 compared with rituximab in patients with previously untreated advanced-stage follicular lymphoma: a randomised, double-blind, parallel-group, non-inferiority phase 3 trial. Lancet Haematol. 2017;4(8):e362-e373. doi:10.1016/S2352-3026(17)30120-5 28712940

[60] Jurczak W, Moreira I, Kanakasetty GB, . Rituximab biosimilar and reference rituximab in patients with previously untreated advanced follicular lymphoma (ASSIST-FL): primary results from a confirmatory phase 3, double-blind, randomised, controlled study. Lancet Haematol. 2017;4(8):e350-e361. doi:10.1016/S2352-3026(17)30106-0 28712941

[61] Kaplanov K, Zaritskiy A, Alexeev S, . Key results of international randomized open-label clinical study of BCD-020 (rituximab biosimilar candidate) in patients with B-Cell non-Hodgkin’s lymphoma. Blood. 2014;124(21):5467. doi:10.1182/blood.V124.21.5467.5467

[62] Pivot X, Georgievich MA, Shamrai V, . Efficacy of HD201 vs referent trastuzumab in patients with ERBB2-positive breast cancer treated in the neoadjuvant setting: a multicenter phase 3 randomized clinical trial. JAMA Oncol. 2022;8(5):698-705. doi:10.1001/jamaoncol.2021.8171 35238873 PMC8895313

[63] Nodehi RS, Kalantari B, Raafat J, . A randomized, double-blind, phase III, non-inferiority clinical trial comparing the efficacy and safety of TA4415V (a proposed trastuzumab biosimilar) and Herceptin (trastuzumab reference product) in HER2-positive early-stage breast cancer patients. BMC Pharmacol Toxicol. 2022;23(1):57. doi:10.1186/s40360-022-00599-x 35902898 PMC9336069

[64] Xu B, Zhang Q, Sun T, ; HLX02-BC01 Investigators. Efficacy, safety, and immunogenicity of HLX02 compared with reference trastuzumab in patients with recurrent or metastatic HER2-positive breast cancer: a randomized phase III equivalence trial. BioDrugs. 2021;35(3):337-350. doi:10.1007/s40259-021-00475-w 33826080 PMC8084805

[65] Alexeev SM, Khorinko AV, Mukhametshina GZ, . Randomized double-blind clinical trial comparing safety and efficacy of the biosimilar BCD-022 with reference trastuzumab. BMC Cancer. 2020;20(1):783. doi:10.1186/s12885-020-07247-9 32819305 PMC7439710

[66] Pegram MD, Bondarenko I, Zorzetto MMC, . PF-05280014 (a trastuzumab biosimilar) plus paclitaxel compared with reference trastuzumab plus paclitaxel for HER2-positive metastatic breast cancer: a randomised, double-blind study. Br J Cancer. 2019;120(2):172-182. doi:10.1038/s41416-018-0340-2 30568294 PMC6342915

[67] von Minckwitz G, Colleoni M, Kolberg HC, . Efficacy and safety of ABP 980 compared with reference trastuzumab in women with HER2-positive early breast cancer (LILAC study): a randomised, double-blind, phase 3 trial. Lancet Oncol. 2018;19(7):987-998. doi:10.1016/S1470-2045(18)30241-9 29880292

[68] Pivot X, Bondarenko I, Nowecki Z, . Phase III, randomized, double-blind study comparing the efficacy, safety, and immunogenicity of SB3 (trastuzumab biosimilar) and reference trastuzumab in patients treated with neoadjuvant therapy for human epidermal growth factor receptor 2-positive early breast cancer. J Clin Oncol. 2018;36(10):968-974. doi:10.1200/JCO.2017.74.0126 29373094

[69] Stebbing J, Baranau Y, Baryash V, . CT-P6 compared with reference trastuzumab for HER2-positive breast cancer: a randomised, double-blind, active-controlled, phase 3 equivalence trial. Lancet Oncol. 2017;18(7):917-928. doi:10.1016/S1470-2045(17)30434-5 28592386

[70] Rugo HS, Barve A, Waller CF, ; Heritage Study Investigators. Effect of a proposed trastuzumab biosimilar compared with trastuzumab on overall response rate in patients with ERBB2 (HER2)-positive metastatic breast cancer: a randomized clinical trial. JAMA. 2017;317(1):37-47. doi:10.1001/jama.2016.18305 27918780

[71] Zhao Z, Zhao L, Xia G, . Efficacy and safety of bevacizumab biosimilar compared with reference bevacizumab in locally advanced and advanced non-small cell lung cancer patients: a retrospective study. Front Oncol. 2023;12:1036906. doi:10.3389/fonc.2022.1036906 36698393 PMC9868544

[72] Deng W, Yang S, Yang C, . Rituximab biosimilar HLX01 versus reference rituximab in the treatment of diffuse large B-cell lymphoma: real-world clinical experience. J Oncol Pharm Pract. Published online July 3, 2022. doi:10.1177/10781552221110470 35786067

[73] Bankar A, Korula A, Abraham A, . Comparison of the efficacy of innovator rituximab and its biosimilars in diffuse large B cell lymphoma patients: a retrospective analysis. Indian J Hematol Blood Transfus. 2020;36(1):71-77. doi:10.1007/s12288-019-01167-w 32174693 PMC7042466

[74] Roy PS, John S, Karankal S, . Comparison of the efficacy and safety of rituximab (Mabthera) and its biosimilar (Reditux) in diffuse large B-cell lymphoma patients treated with chemo-immunotherapy: a retrospective analysis. Indian J Med Paediatr Oncol. 2013;34(4):292-298. doi:10.4103/0971-5851.125248 24604960 PMC3932598

[75] Eser K, Sezer E, Erçolak V, İnal A. Evaluation of biosimilar trastuzumab MYL-1401O in HER2-positive breast cancer. Am J Manag Care. 2023;29(2):e36-e42. doi:10.37765/ajmc.2023.89234 36811986

[76] Yang C, Khwaja R, Tang P, Nixon N, King K, Lupichuk S. A review of trastuzumab biosimilars in early breast cancer and real world outcomes of neoadjuvant MYL-1401O versus reference trastuzumab. Curr Oncol. 2022;29(6):4224-4234. doi:10.3390/curroncol29060337 35735446 PMC9221768

[77] Park JH, Yeo JH, Kim YS, . Efficacy and safety of trastuzumab biosimilar (CT-P6) compared with reference trastuzumab in patients with HER2-positive advanced gastric cancer: a retrospective analysis. Am J Clin Oncol. 2022;45(2):61-65. doi:10.1097/COC.0000000000000887 34991106 PMC8781232

[78] Liu Z, Guan Y, Yao Y, . Effectiveness and safety of Zercepac and reference trastuzumab in the neoadjuvant setting for early-stage breast cancer: a retrospective cohort study. J Oncol. 2022;2022:9998114. doi:10.1155/2022/9998114 36385963 PMC9649327

[79] Bernat-Peguera A, Trigueros M, Ferrando-Díez A, . Efficacy of CT-P6 (trastuzumab biosimilar) versus reference trastuzumab in combination with pertuzumab in HER2-positive early-stage breast cancer: preclinical and real-life clinical data. Breast. 2022;62:1-9. doi:10.1016/j.breast.2022.01.007 35078146 PMC8787779

[80] Bae SJ, Kim JH, Ahn SG, . Real-world clinical outcomes of biosimilar trastuzumab (CT-P6) in HER2-positive early-stage and metastatic breast cancer. Front Oncol. 2021;11:689587. doi:10.3389/fonc.2021.689587 34150658 PMC8213064

[81] Sherman RE, Anderson SA, Dal Pan GJ, . Real-world evidence—what is it and what can it tell us? N Engl J Med. 2016;375(23):2293-2297. doi:10.1056/NEJMsb1609216 27959688

[82] Simoens S, Verbeken G, Huys I. Biosimilars and market access: a question of comparability and costs? Target Oncol. 2012;7(4):227-231. doi:10.1007/s11523-011-0192-7 22249657

[83] Renwick MJ, Smolina K, Gladstone EJ, Weymann D, Morgan SG. Postmarket policy considerations for biosimilar oncology drugs. Lancet Oncol. 2016;17(1):e31-e38. doi:10.1016/S1470-2045(15)00381-2 26758759

[84] Chai Q, Wen H, Lang Y, Zhang L, Song Y, Liu X. Budget impact analysis of the introduction of a trastuzumab biosimilar for HER2-positive breast cancer in China. Clin Drug Investig. 2022;42(11):937-947. doi:10.1007/s40261-022-01197-9 36115003

[85] Andersen JT, Jensen TB. Variation in biosimilar uptake in Europe. JAMA Intern Med. 2021;181(3):403-404. doi:10.1001/jamainternmed.2020.6567 33284317

[86] Jensen TB, Kim SC, Jimenez-Solem E, Bartels D, Christensen HR, Andersen JT. Shift from adalimumab originator to biosimilars in Denmark. JAMA Intern Med. 2020;180(6):902-903. doi:10.1001/jamainternmed.2020.0338 32227137 PMC7105946

[87] Azuz S, Newton M, Bartels D, Poulsen BK. Uptake of biosimilar trastuzumab in Denmark compared with other European countries: a comparative study and discussion of factors influencing implementation and uptake of biosimilars. Eur J Clin Pharmacol. 2021;77(10):1495-1501. doi:10.1007/s00228-021-03155-4 34008071 PMC8440249

[88] Yang J, Liu R, Ektare V, Stephens J, Shelbaya A. Does biosimilar bevacizumab offer affordable treatment options for cancer patients in the USA? a budget impact analysis from US commercial and Medicare payer perspectives. Appl Health Econ Health Policy. 2021;19(4):605-618. doi:10.1007/s40258-021-00637-5 33506318 PMC8270829

[89] Nabhan C, Valley A, Feinberg BA. Barriers to oncology biosimilars uptake in the United States. Oncologist. 2018;23(11):1261-1265. doi:10.1634/theoncologist.2018-0066 30049886 PMC6291332

[90] Jensen TB, Bartels D, Sædder EA, . The Danish model for the quick and safe implementation of infliximab and etanercept biosimilars. Eur J Clin Pharmacol. 2020;76(1):35-40. doi:10.1007/s00228-019-02765-3 31677117

[91] Yuan J, Lu ZK, Xiong X, Jiang B. Lowering drug prices and enhancing pharmaceutical affordability: an analysis of the national volume-based procurement (NVBP) effect in China. BMJ Glob Health. 2021;6(9):e005519. doi:10.1136/bmjgh-2021-005519 34518200 PMC8438819

[92] Wu Q, Lian Z, Wang X, . Factors associated with the uptake of biosimilars for breast cancer treatment from the perspectives of physicians and patients—evidence from China. Front Pharmacol. 2023;13:1044798. doi:10.3389/fphar.2022.1044798 36712662 PMC9877225

[93] Lurie P, Chahal HS, Sigelman DW, Stacy S, Sclar J, Ddamulira B. Comparison of content of FDA letters not approving applications for new drugs and associated public announcements from sponsors: cross sectional study. BMJ. 2015;350:h2758. doi:10.1136/bmj.h2758 26063327 PMC4462714

