# Supplementary material for: Clinical Benefit, Price, and Uptake for Cancer Biosimilars vs Reference Drugs in China: A Systematic Review and Meta-Analysis
Source: JAMA Netw Open. 2023 Oct 12;6(10):e2337348. doi: 10.1001/jamanetworkopen.2023.37348 (PMC10570888; doi:10.1001/jamanetworkopen.2023.37348)
Supplement: Supplement 1. — eAppendix 1. Detailed Description of the Eligibility and Exclusion Criteria eAppendix 2. Evidence Sources and Search Strategy eAppendix 3. Price and Uptake of Biosimilar and Reference Drugs eTable 1. Comparison of Regulatory Factors for Biosimilar Oncology Drugs in the NMPA, FDA, EMA, and PMDA eTable 2. Individual Biosimilar Guidelines Issued by the NMPA in China eTable 3. Cancer Biosimilars Approved by the FDA, EMA, PMDA, and NMPA as of February 1, 2023 eTable 4. Biosimilar Products Approved in China eTable 5. Approved and In-Development Oncology Biosimilar Products in China as of February 1, 2023 eTable 6. Demographic and Clinical Characteristics of the Included RCTs eTable 7. Demographic and Clinical Characteristics of the Included Cohort Studies eTable 8. Results of the Risk of Bias Assessment of the RCTs Based on the Cochrane Collaboration’s Tools eTable 9. Results of the Risk of Bias Assessment of the Cohort Studies Based on the Newcastle-Ottawa Risk of Bias Assessment Tool eTable 10. Characteristics of Pivotal Clinical Trials Used to Support Approval of Cancer Biosimilars by the NMPA, FDA, EMA, and PMDA eTable 11. Subgroup Analysis of Cancer Biosimilars Compared With Reference Drugs Derived From Randomized Clinical Trials eTable 12. Pooled Analysis of Efficacy, Safety, and Immunogenicity of Cancer Biosimilars Derived From Randomized Clinical Trials eTable 13. Pooled Analysis of Efficacy, Safety, and Immunogenicity of Cancer Biosimilars Derived From Cohort Studies eTable 14. Weighted Mean Price per Unit of Rituximab, Bevacizumab, Trastuzumab, and Their Biosimilars Between 2015 and 2022 eTable 15. Trends in the Uptake Rate of Rituximab, Bevacizumab, and Trastuzumab Biosimilars vs Reference Drugs After Market Entry as of December 2022 eTable 16. Uptake Rates of Cancer Biosimilars vs Reference Drugs in the First Year, Second Year, and Latest Time on the Market eTable 17. Reimbursable Cancer Indications for the Biosimilars and Reference Drugs of Bevacizumab, Ritux [file jamanetwopen-e2337348-s001.pdf]

Supplementary Online Content

Luo X, Du X, Li Z, et al. Clinical benefit, price, and uptake for cancer biosimilars vs reference drugs in China: a systematic review and meta-analysis. *JAMA Netw Open*. 2023;6(10):e2337348. doi:10.1001/jamanetworkopen.2023.37348

eAppendix 1. Detailed Description of the Eligibility and Exclusion Criteria
eAppendix 2. Evidence Sources and Search Strategy
eAppendix 3. Price and Uptake of Biosimilar and Reference Drugs
eTable 1. Comparison of Regulatory Factors for Biosimilar Oncology Drugs in the NMPA, FDA, EMA, and PMDA
eTable 2. Individual Biosimilar Guidelines Issued by the NMPA in China
eTable 3. Cancer biosimilars Approved by the FDA, EMA, PMDA, and NMPA as of February 1, 2023
eTable 4. Biosimilar Products Approved in China
eTable 5. Approved and In-Development Oncology Biosimilar Products in China as of February 1, 2023
eTable 6. Demographic and Clinical Characteristics of the Included RCTs
eTable 7. Demographic and Clinical Characteristics of the Included Cohort Studies
eTable 8. Results of the Risk of Bias Assessment of the RCTs Based on the Cochrane Collaboration’s Tools
eTable 9. Results of the Risk of Bias Assessment of the Cohort Studies Based on the Newcastle-Ottawa Risk of Bias Assessment Tool
eTable 10. Characteristics of Pivotal Clinical Trials Used to Support Approval of Cancer Biosimilars by the NMPA, FDA, EMA, and PMDA
eTable 11. Subgroup Analysis of Cancer Biosimilars Compared With Reference Drugs Derived From Randomized Clinical Trials
eTable 12. Pooled Analysis of Efficacy, Safety, and Immunogenicity of Cancer Biosimilars Derived From Randomized Clinical Trials
eTable 13. Pooled Analysis of Efficacy, Safety, and Immunogenicity of Cancer Biosimilars Derived From Cohort Studies
eTable 14. Weighted Mean Price per Unit of Rituximab, Bevacizumab, Trastuzumab, and Their Biosimilars Between 2015 and 2022
eTable 15. Trends in the Uptake Rate of Rituximab, Bevacizumab, and Trastuzumab Biosimilars vs Reference Drugs After Market Entry as of December 2022
eTable 16. Uptake Rates of Cancer Biosimilars vs Reference Drugs in the First Year, Second Year, and Latest Time on the Market
eTable 17. Reimbursable Cancer Indications for the Biosimilars and Reference Drugs of Bevacizumab, Rituximab, and Trastuzumab in the National Reimbursement Drug List in China as of July 1, 2023
eTable 18. Comparison of the Approved Indications of Cancer Biosimilars vs Reference Drugs by the NMPA, FDA, EMA, and PMDA as of July 2023
eFigure 1. Development History of Biosimilars in China
eFigure 2. Flowchart of Included Studies for the Systematic Review and Meta-analysis
eFigure 3. Meta-analysis of the Primary Efficacy End Points Between Bevacizumab Biosimilars and the Originator Derived from RCTs
eFigure 4. Meta-analysis of the Primary Efficacy End Points Between Rituximab Biosimilars and the Originator Stratified by Cancer Type Derived From RCTs
eFigure 5. Meta-analysis of the Primary Efficacy End Points Between Trastuzumab Biosimilars and the Originator Stratified by Cancer Type Derived From RCTs
eFigure 6. Sensitivity Analysis of Primary Efficacy End Points for Bevacizumab Derived From RCTs

© 2023 Luo X et al. *JAMA Network Open.*

eFigure 7. Sensitivity Analysis of Primary Efficacy End Points for Rituximab Derived From RCTs
eFigure 8. Sensitivity Analysis of Primary Efficacy End Points for Trastuzumab Derived From RCTs
eFigure 9. Publication Bias Assessment of Primary Efficacy End Points for Bevacizumab Derived From RCTs
eFigure 10. Publication Bias Assessment of Primary Efficacy End Points for Rituximab Derived From RCTs
eFigure 11. Publication Bias Assessment of Primary Efficacy End Points for Trastuzumab Derived From RCTs
eReferences

This supplementary material has been provided by the authors to give readers additional information about their work.

© 2023 Luo X et al. *JAMA Network Open.*

eAppendix 1. A detailed description of the eligibility and exclusion criteria

Eligibility criteria

Participants: the patient was diagnosed with a malignant cancer that met the criteria of international guidelines. We did not limit the age, sex, number of lines of treatment, stage of the cancer, and other primary demographic characteristics of the patients with the cancer.

Intervention and Comparators: the intervention group was any of the rituximab biosimilars, trastuzumab biosimilars, and bevacizumab biosimilars (As of February 2023, only these three therapeutic cancer biosimilars have been approved in China). The comparators were their reference drug. We did not have any restrictions on dosage, treatment duration, treatment regimen, or number of patients included.

Study designs: we included randomized controlled trials (RCT) and cohort studies (comparative studies of biosimilars and originator drugs). The RCT was limited to explicit pre-specification for a non-inferiority or equivalence trial design. In addition, the primary endpoints of randomized controlled trials should include at least one of the efficacy endpoints. For the cohort studies, we did not restrict the study design (equipotent or noninferiority) and did not set out to have at least one primary efficacy endpoint, considering the evidence from the cohort studies as complementary evidence to the RCT.

Exclusion criteria

(1) Non-comparative studies (e.g., reviews, expert commentaries, editorials, and clinical guidelines);

(2) Studies without comparators;

(3) The primary endpoints of the randomized controlled trials were pharmacokinetic parameters or safety;

(4) The data for the cohort study design were derived from survey research.

© 2023 Luo X et al. *JAMA Network Open.*

eAppendix 2. Evidence sources and search strategy.

We conducted a systematic search based on PICO items in Embase, PubMed, Cochrane Library and ClinicalTrials.gov from database inception to February 1, 2023. XXL and XD were responsible for screening documents independently that met the inclusion criteria by title, abstract, and full text. If there was an inconsistency, it is resolved by the third reviewer.

Participants: Carcinoma or cancer or adenocarcinoma or tumor or oncology or neoplasm or malignant or lymphoma.

Intervention: biosimilar or 'follow-on biologic' or 'subsequent-entry biologic' or 'similar biologic' or 'highly similar' OR 'biologic copy' or 'subsequent-entry biological' or 'subsequent-entry biologic' or 'subsequent entry biologic' or 'subsequent entry biological' or 'biosimilarity' or Riabni or Ruxience or Truxima or rituximab-arrx or rituximab-pvvr or rituximab-abbs or Herzuma or trastuzumab-pkrb or Kanjinti ortrastuzumab-anns or Ogivri or trastuzumab-dkst or Ontruzant or trastuzumab-dttb or Trazimera or trastuzumab-qyyp or Mvasi or bevacizumab-awwb or Zirabev or bevacizumab-bvzr.

Comparators: Avastin or bevacizumab or Herceptin or trastuzumab or Rituxan or Rituximab or reference or originator or brand name or brand-name.

Study designs: Compared or comparison or compare or contrast or versus or equivalence or switching or equivalent or equivalence or equivalence or comparing.

The research strategy was similar to the previous study [JAMA Oncol. 2022;8(4):537-545] and the search strategy in this study as follow:

**Search strategy of PubMed**	
#1	Carcinoma[Title/Abstract] OR cancer[Title/Abstract] OR adenocarcinoma[Title/Abstract] OR tumor[Title/Abstract] OR oncology[Title/Abstract] OR neoplasm[Title/Abstract] OR malignant[Title/Abstract] OR lymphoma[Title/Abstract]
#2	biosimilar[Title/Abstract] OR 'follow-on biologic'[Title/Abstract] OR 'subsequent-entrybiologic'[Title/Abstract] OR 'similar biologic'[Title/Abstract] OR 'highly similar'[Title/Abstract] OR 'biologic copy'[Title/Abstract] OR 'subsequent-entry biological'[Title/Abstract] OR 'subsequent-entry biologic'[Title/Abstract] OR 'subsequent entry biologic'[Title/Abstract] OR 'subsequent entry biological'[Title/Abstract] OR 'biosimilarity'[Title/Abstract] OR Riabni[Title/Abstract] OR Ruxience[Title/Abstract] OR Truxima[Title/Abstract] OR rituximab-arrx[Title/Abstract] OR rituximab-pvvr[Title/Abstract] OR rituximab-abbs[Title/Abstract] OR Herzuma[Title/Abstract] OR trastuzumab-pkrb[Title/Abstract] OR Kanjinti ortrastuzumab-anns[Title/Abstract] OR Ogivri[Title/Abstract] OR trastuzumab-dkst[Title/Abstract] OR Ontruzant[Title/Abstract] OR trastuzumab-dttb[Title/Abstract] OR Trazimera[Title/Abstract] OR trastuzumab-qyyp[Title/Abstract] OR Mvasi[Title/Abstract] OR bevacizumab-awwb[Title/Abstract] OR Zirabev[Title/Abstract] OR bevacizumab-bvzr[Title/Abstract]
#3	Avastin[Title/Abstract] OR bevacizumab[Title/Abstract] OR Herceptin[Title/Abstract] OR trastuzumab[Title/Abstract] OR Rituxan[Title/Abstract] OR Rituximab[Title/Abstract] OR reference[Title/Abstract] OR originator[Title/Abstract] OR brand name[Title/Abstract] OR brand-name[Title/Abstract]
#4	Compared[Title/Abstract] OR comparison[Title/Abstract] OR compare[Title/Abstract] OR contrast[Title/Abstract] OR versus[Title/Abstract] OR equivalence[Title/Abstract] OR switching[Title/Abstract] OR equivalent[Title/Abstract] OR equivalence[Title/Abstract] OR equivalence[Title/Abstract] OR comparing[Title/Abstract]
#5	#1 and #2 and #3 and #4
**Search strategy of Embase**	

© 2023 Luo X et al. *JAMA Network Open.*

#1	carcinoma:ab,ti OR cancer:ab,ti OR adenocarcinoma:ab,ti OR tumor:ab,ti OR oncology:ab,ti OR neoplasm:ab,ti OR malignant:ab,ti OR lymphoma:ab,ti
#2	biosimilar:ab,ti OR 'follow-on biologic':ab,ti OR 'subsequent-entrybiologic':ab,ti OR 'similar biologic':ab,ti OR 'highly similar':ab,ti OR 'biologic copy':ab,ti OR 'subsequent-entry biological':ab,ti OR 'subsequent-entry biologic':ab,ti OR 'subsequent entry biologic':ab,ti OR 'subsequent entry biological':ab,ti OR 'biosimilarity':ab,ti OR riabni:ab,ti OR ruxience:ab,ti OR truxima:ab,ti OR 'rituximab arrx':ab,ti OR 'rituximab pvvr':ab,ti OR 'rituximab abbs':ab,ti OR herzuma:ab,ti OR 'trastuzumab pkrb':ab,ti OR 'kanjinti ortrastuzumab-anns':ab,ti OR ogivri:ab,ti OR 'trastuzumab dkst':ab,ti OR ontruzant:ab,ti OR 'trastuzumab dttb':ab,ti OR trazimera:ab,ti OR 'trastuzumab qyyp':ab,ti OR mvasi:ab,ti OR 'bevacizumab awwb':ab,ti OR zirabev:ab,ti OR 'bevacizumab bvzr':ab,ti
#3	avastin:ab,ti OR bevacizumab:ab,ti OR herceptin:ab,ti OR trastuzumab:ab,ti OR rituxan:ab,ti OR rituximab:ab,ti OR reference:ab,ti OR originator:ab,ti OR 'brand name':ab,ti
#4	compared:ab,ti OR comparison:ab,ti OR compare:ab,ti OR contrast:ab,ti OR versus:ab,ti OR switching:ab,ti OR equivalent:ab,ti OR equivalence:ab,ti OR comparing:ab,ti
#5	#1 and #2 and #3 and #4
**Cochrane library**	
#1	(Carcinoma or cancer or adenocarcinoma or adenocarcinoma or malignancy or tumor or oncology or neoplasm or malignant or malignant or lymphoma):ti,ab,kw
#2	(biosimilar or 'follow-on biologic' or 'subsequent-entrybiologic' or 'similar biologic' or 'highly similar' OR 'biologic copy' or 'subsequent-entry biological' or 'subsequent-entry biologic' or 'subsequent entry biologic' or 'subsequent entry biological' or 'biosimilarity' or Riabni or Ruxience or Truxima or rituximab-arrx or rituximab-pvvr or rituximab-abbs or Herzuma or trastuzumab-pkrb or Kanjinti ortrastuzumab-anns or Ogivri or trastuzumab-dkst or Ontruzant or trastuzumab-dttb or Trazimera or trastuzumab-qyyp or Mvasi or bevacizumab-awwb or Zirabev or bevacizumab-bvzr):ti
#3	(Avastin or bevacizumab or Herceptin or trastuzumab or Rituxan or Rituximab or reference or originator or brand name or brand-name):ti
#4	(Carcinoma or cancer or adenocarcinoma or adenocarcinoma or malignancy or tumor or oncology or neoplasm or malignant or malignant or lymphoma):ti,ab,kw
#5	#1 and #2 and #3 and #4
**Search strategy of Clinicaltrials.gov**	

© 2023 Luo X et al. *JAMA Network Open.*

#1	(Rituximab OR Trastuzumab OR Bevacizumab) AND Biosimilar AND ("compared" OR "comparison" OR "compare" OR "contrast" OR "versus" OR "equivalence" OR "switching" OR "equivalent" OR "equivalence" OR "bioequivalence" OR "comparing") | Completed WITH RESULTS

© 2023 Luo X et al. *JAMA Network Open.*

eAppendix 3. Price and uptake of biosimilar and reference drugs.

The Chinese healthcare system is established under a multi-tiered governance structure, involving the central, provincial, regional, and county governments[1,2]. The majority of drug procurement occurs through centralized bidding at the provincial or city level, which may result in regional variations[1]. Therefore, XXL and XD extracted the winning prices of each biosimilar and originator drug between 2015-2022 from different provinces or cities and calculated the weighted average price of the drug using the number of population as weights. The exchange rate of the U.S. dollar to the Chinese yuan was 6.9. Similar to the previous study[1], we adjusted the price of biosimilars and their reference drug based on the annual consumption index published by the National Bureau of Statistics of China given the effect of inflation.

The healthcare facilities are categorized into primary healthcare facilities, first, second and third level hospitals in China. Usually, a higher level means a greater level of treatment. In this study, we used the national hospital sales volume from Pharnexcloud databse module[3], which covered the sales volume of cancer biosimilars in secondary and higher hospitals across the country, as no public database of drug sales volume was available. XXL and XD extracted the sales volume of each cancer biosimilar and originator drugs for each quarter since the biosimilar was introduced to the market. For multiple biosimilars that were approved (e.g. eight bevacizumab biosimilars were approved in China), we aggregated the sales volume of these biosimilars. The annual uptake rate for biosimilars is calculated by dividing the one-year sales of biosimilars by the one-year sales of their reference drugs.

The cut-off date for price uptake rates of biosimilars was December 31, 2022.

© 2023 Luo X et al. *JAMA Network Open.*

eTable 1. Comparison of regulatory factors for biosimilar oncology drugs in the NMPA, FDA, EMA, and PMDA.

Items	NMPA	FDA	EMA	PMDA
**Key patent expiration of Bevacizumab**	2018	2017	2018	2017
**Key patent expiration of R ituximab**	2013	2018	2013	NA
**Key patent expiration of Trastuzumab**	NA	2019	2014	NA
**Patent challenges**	Rarely occur	Either formal litigation in court or administrative challenges (inter partes reviews) occur for 56% of approved oncology biosimilars	Rarely occur	In January, 2019, Celltrion won two appeal cases claiming that Roche's patent for trastuzumab for the treatment of HER2-positive breast cancer was invalid;Tokyo District Court dismissed Genentech's claim against Sandoz-Kirin to prohibit the marketing of a rituximab biosimilar
**Indication extrapolation**	Primary consideration is given to core patents, with more limited impact of patents on indications; Exclusivity periods have not been established	Impact of core and indication patents; Impact of exclusivity period (especially for 7-year orphan drug designation)	Impact of core and indication patents; Impact of exclusivity period	Impact of core and indication patents; Impact of exclusivity period.
**Reimbursement policy**	Determined by China National Healthcare Security Administration	Determined by individual insurers	Determined by national authorities	Determined centrally by Japan's Central Social Medical Insurance Council of the Ministry of Health, Labour, and Welfare
**Switching of patients from a reference biologic to a biosimilar**	No regulatory designation for interchangeability	Not permitted unless the biosimilar has been formally designated as interchange-able by FDA	No regulatory designation for interchangeability	Permitted under the PMDA's Guideline for the quality, safety, and efficacy assurance of follow-on biologics (revised Feb 2, 2020)

© 2023 Luo X et al. *JAMA Network Open.*

**Pharmacist substitution of a reference biologic with a biosimilar**	Not permitted	Not permitted unless FDA has designated the biosimilar as interchangeable with the reference biologic, and even if it has 45 US states do not permit substitution unless patient consent, doctor consent, or both have been documented	Permitted in several EU countries	Not permitted
**Price**	Price competition; Price discounts of biosimilars in China range from 10-31% in 2022 (the data derived from eTable 14 of this study)	Price competition; Price discounts of biosimilars in the USA range from 10% to 33%; Impact of pay for delaying agreements between biosimilars and originator drugs	Price competition; European price discounts for biosimilars average 30%	The Japanese Ministry of Health, Labour, and Welfare sets prices for biosimilars at a 30% discount in principle, and at 40% if the biosimilar has ten or more approved indications

Items of FDA, EMA, and PMDA derived from published literature (Lancet Oncol. 2020;21(12):e575-e588).
Abbreviations: NA, not applicable; FDA, US Food and Drug Administration; EMA, European Medicines Agency; PMDA, Japanese Pharmaceuticals and Medical Devices Agency; NMPA, National Medical Products Administration.

© 2023 Luo X et al. *JAMA Network Open.*

eTable 2. Individual biosimilar guidelines issued by the NMPA in China(n=10).

Initiation date	Guidelines	Biosimilar approval or not
2020-05-28	Clinical trial design guidelines for liraglutide injection biosimilars	No
2020-07-20	Guidelines for clinical trials of rituximab injection biosimilars	Yes
2020-07-20	Guidelines for clinical trials of trastuzumab biosimilars for injection	Yes
2020-08-03	Clinical trial guidelines for adalimumab injection biosimilars	Yes
2020-08-03	Clinical trial guidelines for bevacizumab injection biosimilars	Yes
2021-02-04	Guidelines for clinical trials of omalizumab biosimilars for injection	No
2021-04-22	Guidelines for clinical trials of tolimumab injection biosimilars	Yes
2021-04-22	Guidelines for clinical trials of patalizumab injection biosimilars	No
2022-01-20	Clinical trial design guidelines for cetuximab injection biosimilars	No
2022-01-26	Guidelines for clinical trial design of teriparatide injection biosimilars	Yes

© 2023 Luo X et al. *JAMA Network Open.*

eTable 3. The cancer biosimilars approved by the FDA, EMA, PMDA, and NMPA (as of February 1, 2023).

Product	NMPA		EMA		FDA		PMDA	
INN	Reference	Biosimilars	Reference	Biosimilars	Reference	Biosimilars	Reference	Biosimilars
Bevacizumab	Avastin (2010)	Ankeda (2019)<br>Dayoutong (2020)<br>Boyounuo (2021)<br>Airuituo (2021)<br>Pubeixi (2021)<br>Beianting (2021)<br>Puxinting (2021)<br>Hanbeitai (2021)	Avastin (2005)	Mvasi (2018)<br>Zirabev (2019)<br>Aybintio (2020)<br>Alymsys (2021)<br>Oyavas (2021)<br>Abevmy (2021)<br>Vegzelma (2022)	Avastin (2004)	Mvasi (2017)<br>Zirabev (2019)<br>Alymsys (2022)<br>Vegzelma (2022)	Avastin (2007)	Bevacizumab(Pfizer) (2019)<br>Bevacizumab (Daiichi Sankyo) (2019)<br>Bevacizumab (Nichiiko) (2021)<br>Bevacizumab (CTNK) (2022)
Rituximab	Mabthera (2000)	Hanlikang (2019)<br>Dabohua (2020)	Mabthera (1998)	Truxima (2017)<br>Rixathon (2017)<br>Ruxience (2020)	Rituxan (1997)	Truxima (2018)<br>Ruxience (2019)<br>Riabni (2020)	Rituxan (2001)	Rituximab (KHK) (2017)<br>Rituximab (Pfizer) (2019)
Trastuzumab	Herceptin (2002)	Hanyouqu (2020)	Herceptin (1998)	Ontruzant (2017)<br>Herzuma (2018)<br>Kanjinti (2018)<br>Trazimera (2018)<br>Ogivri (2018)<br>Zercepac (2020)	Herceptin (1998)	Ogivri (2017)<br>Herzuma (2018)<br>Ontruzant (2019)<br>Trazimera (2019)<br>Kanjinti (2019)	Herceptin (2001)	Trastuzumab (NK/CTH) (2017)<br>Trastuzumab (Daiichi Sankyo) (2018)<br>Trastuzumab (Pfizer) (2018)

The reference products and biosimilars are expressed as trade names. The bracketed content indicates the year of approval.
Abbreviations: INN, International Nonproprietary Names. FDA, US Food and Drug Administration. EMA, European Medicines Agency. PMDA, Japanese Pharmaceuticals and Medical Devices Agency. NMPA, National Medical Products Administration.

© 2023 Luo X et al. *JAMA Network Open.*

eTable 4. Biosimilar products approved in China (n=23).

Numbers	Biosimilars		Reference drugs		Time difference[a]
	Names	Approval date	Names	Approval date	
1	Rituximab-HLX01	2019-02-22	Rituximab	2000-03-15	19.0
2	Rituximab-IBI301	2020-09-30			
3	Teriparatide-SAL001	2019-09-29	Teriparatide	2011-03-04	8.6
4	Adalimumab-BAT1406	2019-11-04	Adalimumab	2010-02-26	9.7
5	Adalimumab-HS016	2019-12-06			
6	Adalimumab-IBI303	2020-09-02			
7	Adalimumab-HLX03	2020-12-02			
8	Adalimumab-TQZ2301	2022-01-18			
9	Adalimumab-UBP1211	2022-03-01			
10	Bevacizumab-QL1101	2019-12-06	Bevacizumab	2010-02-26	9.8
11	Bevacizumab-IBI305	2020-06-17			
12	Bevacizumab-LY01008	2021-04-30			
13	Bevacizumab-BP102	2021-06-22			
14	Bevacizumab-BAT1706	2021-11-17			
15	Bevacizumab-MIL60	2021-11-24			
16	Bevacizumab-TAB008	2021-11-30			
17	Bevacizumab-HLX04	2021-11-30			
18	Trastuzumab-HL02	2020-08-12	Trastuzumab	2002-09-05	17.9
19	Infliximab-CMAB008	2021-07-12	Infliximab	2006-05-17	15.2
20	Infliximab-HS626	2021-09-24			
21	Infliximab-GB242	2022-02-23			
22	Insulin aspart-HS005	2021-09-13	Insulin aspart	2002-08-16	19.1
23	Denosumab-LY06006	2022-11-08	Denosumab	2019-05-21	3.5

[a]Time difference is defined as the duration between the first approved biosimilar and the reference drug.

© 2023 Luo X et al. *JAMA Network Open.*

eTable 5. The number of approved and in-development oncology biosimilars products in China (As of February 1, 2023)

Biosimilars	I phase	III phase	NDA review	Approved
Bevacizumab	5	9	2	8
Rituximab	2	5	2	2
Trastuzumab	2	7	3	1

© 2023 Luo X et al. *JAMA Network Open.*

eTable 6. Demographic and clinical characteristics of the included RCTs (n=39).

Number	First Author	Year	Clinical trial number	Sponsor	Country	Study design	MRCT	Cancer types	Biosimilar	Originator	Sex (M/F)	No. Patients in biosimilar	No. Patients in originator	Primary efficacy endpoints (Times)	study duration[a] (month)
**Bevacizumab biosimilars vs Bevacizumab**															
**1**	Stroyakovskiy et al.[1]	2022	NCT01763645	Biocad	Russian	equivalence	Yes	advanced non-squamous NSCLC	BCD-021	bevacizumab	230/110	212	145	ORR (18w)	25.37
**2**	Thatcher et al.[2]	2019	NCT01966003	Amgen	US	equivalence	Yes	advanced nonsquamous NSCLC	ABP215	bevacizumab	NA	328	314	ORR (25w)	20.63
**3**	Reinmuth et al.[3]	2019	NCT02364999	Pfizer	US	equivalence	Yes	newly diagnosed Stage IIIB or IV NSCLC	PF-06439535	bevacizumab	467/252	358	361	ORR (19w)	25.37
**4**	Reck et al.[4]	2020	NCT02754882	Samsung	Korea	equivalence	Yes	metastatic or recurrent NSCLC	SB8	bevacizumab	508/255	379	384	ORR (24w)	18.93
**5**	Yang et al.[5]	2019	NCT02954172	Innovent	China	equivalence	No	metastatic or recurrent non-squamous NSCLC	IBI305	bevacizumab	279/162	224	226	ORR (18w)	23.00
**6**	Trukhin et al.[6]	2021	NCT03296163	mAbxience	Spain	equivalence	Yes	stage IIIB/IV non-squamous NSCLC	MB02	bevacizumab	383/244	315	312	ORR (18w)	17.07
**7**	Rezvani et al.[7]	2020	NCT03288987	AryoGen	Iran	noninferiority	No	metastatic CRC	BE1040V	bevacizumab	91/35	82	42	PFS (NA)	22.13
**8**	Qin et al.[8]	2021	NCT03511963	Henlius	China	equivalence	No	metastatic CRC	HLX04	bevacizumab	404/271	340	337	PFSR (36w)	24.57
**9**	Shi et al.[9]	2021	NCT03533127	Luye	China	equivalence	No	unresectable, metastatic, or recurrent non-squamous NSCLC	LY01008	bevacizumab	352/237	324	325	ORR (18w)	31.47
**10**	Chu et al[10]	2021	NCT03169335	Qilu	China	equivalence	No	untreated advanced non-squamous NSCLC	QL1101	bevacizumab	318/217	269	266	ORR (18w)	15.07
**11**	Hengrui[11] (Sponsor)	2022	NCT05169801	HengRui	China	equivalence	No	metastatic or recurrent non-squamous NSCLC	BP102	bevacizumab	NA	262	255	ORR (18w)	21.13
**12**	Wan et al.[12]	2021	NCT03196986	Mabworks	China	equivalence	No	metastatic or recurrent non-squamous NSCLC	MIL60	bevacizumab	325/183	257	260	ORR (12w)	23.87
**13**	Syrigos et al.[13]	2021	NCT02810457	Centus	UK	equivalence	Yes	advanced/recurrent non-squamous NSCLC	FKB238	bevacizumab	483/248	364	367	ORR (30m)	28.97
**14**	Kim et al.[14]	2022	NCT02272413	Boehringer Ingelheim	Germany	equivalence	Yes	recurrent or metastatic (stage IV) NSCLC	BI695502	bevacizumab	417/246	338	333	ORR (18w)	24.10

© 2023 Luo X et al. *JAMA Network Open.*

Number	First Author	Year	Clinical trial number	Sponsor	Country	Study design	MRCT	Cancer types	Biosimilar	Originator	Sex (M/F)	No. Patients in biosimilar	No. Patients in originator	Primary efficacy endpoints (Times)	study duration[a] (month)
**15**	Socinski et al.[15]	2021	EudraCT no. 2015-005141-32	Mylan GmbH	US	equivalence	Yes	IV unresectable, recurrent, or metastatic NSCLC	MYL-14020	bevacizumab	424/247	337	334	ORR (18w)	28.83
**16**	Chen et al.[16]	2022	NCT03329911	Bio-Thera	China	equivalence	Yes	advanced nonsquamous NSCLC	BAT1706	bevacizumab	NA	325	326	ORR (18w)	24.87
**17**	Lu et al.[17]	2023	NCT05427305	TOT	China	equivalence	No	metastatic EGFR wild-type non-squamous NSCLC	TAB 008	bevacizumab	457/194	277	272	ORR (18w)	29.53
**18**	Verschraegen et al.[18]	2022	NCT03676192	Celltrion	Korea	equivalence	No	metastatic or recurrent non-squamous NSCLC.	CT-P16	bevacizumab	397/149	342	347	ORR (21w)	28.50
**Rituximab biosimilars vs Rituximab**															
**1**	Kaplanov et al.[19]	2014	NA	Biocad	Russia	noninferiority	Yes	B-Cell Non-Hodgkin's Lymphoma/follicular non-Hodgkin's lymphoma	BCD-020	rituximab	NA	46	46	ORR (NA)	23.73
**2**	Toogeh et al.[20]	2018	IRCT201305296302N5	AryoGen	Iran	noninferiority	No	untreated or recurrent B-CLL	Zytux	rituximab	57/13	35	35	ORR (NA)	8.00
**3**	Jurczak et al.[21]	2017	NCT01419665	Sandoz	Switzerland	equivalence	Yes	untreated, advanced stage (Ann Arbor stage III or IV) follicular lymphoma	GP2013	rituximab	277/350	314	315	ORR (21w)	43.90
**4**	Kim et al.[22]	2017	NCT02162771	Celltrion	Korea	noninferiority	No	newly diagnosed advancedstage follicular lymphoma	CT-P10	rituximab	63/77	70	70	ORR (24w)	18.23
**5**	Ogura et al.[23]	2018	NCT02260804	Celltrion	Korea	equivalence	No	untreated low-tumour-burden follicular lymphoma	CT-P10	rituximab	123/145	130	128	ORR (7m)	26.23
**6**	Poddunaya et al.[24]	2020	NCT01701232	Biocad	Russia	equivalence	Yes	indolent non-Hodgkin lymphomas	BCD-020	rituximab	84/90	89	85	ORR (4w)	64.97

© 2023 Luo X et al. *JAMA Network Open.*

Number	First Author	Year	Clinical trial number	Sponsor	Country	Study design	MRCT	Cancer types	Biosimilar	Originator	Sex (M/F)	No. Patients in biosimilar	No. Patients in originator	Primary efficacy endpoints (Times)	study duration[a] (month)
**7**	Candelaria et al.[25]	2019	NCT02268045	mAbxience	Spain	noninferiority	Yes	DLBCL with untreated CD20þ, at stage I (only with bulky disease) or stages II to IV disease	RTXM83	rituximab	155/117	136	136	ORR (18w)	36.53
**8**	Sharman et al.[26]	2020	NCT02213263	Pfizer	US	equivalence	Yes	Low Tumor Burden, Follicular Lymphoma	PF-05280586	rituximab	178/216	196	198	ORR (26w)	37.30
**9**	Niederwieser et al.[27]	2020	NCT02747043	Amgen	US	equivalence	Yes	Follicular Lymphoma	ABP 798	rituximab	130/126	128	128	ORR (28w)	37.63
**10**	Archigen[28] (Sponor)	2020	NCT02809053	Archigen	Korea	equivalence	Yes	patients with low Tumor Burden Follicular lymphoma	SAIT101	rituximab	141/174	157	158	ORR (28w)	30.33
**11**	Shi et al.[29]	2020	NCT02787239； CTR20150583	Henlius	China	equivalence	No	treatment-naïve, CD20-positive DLBCL	HLX01	rituximab	220/182	201	206	ORR (18w)	31.43
**12**	Song et al.[30]	2021	NCT02867566	Innovent	China	equivalence	No	untreated CD20 DLBCL	IBI301	rituximab	214/202	209	210	ORR (18w)	36.97
**Trastuzumab biosimilars vs Trastuzumab**															
**1**	Stebbing et al.[31]	2017	NCT02162667	Celltrion	Korea	equivalence	Yes	neoadjuvant treatment of HER2-positive early-stage breast cancer.	CT-P6	trastuzumab	0/549	271	278	pCR (30w)	24.17
**2**	Rugo et al.[32]	2017	NCT02472964	Mylan Inc.	UK	equivalence	Yes	ERBB2 (HER2)–Positive Metastatic Breast Cancer	MYL-14010	trastuzumab	0/528	250	250	ORR (24w)	44.63
**3**	Von Minckwitz et al.[33]	2018	NCT01901146/CT 2012-004319-29	Amgen	US	equivalent	Yes	Early BC	ABP 980	trastuzumab	0/725	364	361	pCR (7w)	36.73
**4**	Pivot et al.[34]	2018	NCT02149524	Samsung	Korea	equivalence	No	Early BC	SB3	trastuzumab	0/875	437	438	pCR (24w)	23.33
**5**	Pegram et al.[35]	2019	NCT01989676	Pfizer	US	equivalence	Yes	HER2-positive metastatic breast cancer	PF-05280014	trastuzumab	0/707	352	355	ORR (25w)	30.40

© 2023 Luo X et al. *JAMA Network Open.*

Number	First Author	Year	Clinical trial number	Sponsor	Country	Study design	MRCT	Cancer types	Biosimilar	Originator	Sex (M/F)	No. Patients in biosimilar	No. Patients in originator	Primary efficacy endpoints (Times)	study duration[a] (month)
**6**	Pivot et al.[36]	2022	NCT03013504	Prestige	Singapore	equivalence	Yes	ERBB2-positive early breast cancer	HD201	trastuzumab	0/502	251	251	pCR (24w)	14.13
**7**	Alexeev et al.[37]	2020	NCT01764022	Biocad	Russian	equivalence	Yes	metastatic HER2(+) breast cancer	BCD-022	trastuzumab	0/225	115	110	ORR (18w)	29.37
**8**	Xu et al.[38]	2021	NCT03084237; EudraCT 2016-000206-10; CTR20160526	Henlius	China	equivalence	Yes	Recurrent or Metastatic HER2-Positive breast cancer	HLX02	trastuzumab	0/649	324	325	ORR (24w)	24.17
**9**	Nodehi et al.[39]	2022	NCT03425656	AryoGen	Iran	noninferiority	No	HER2-positive early-stage breast cancer patients	TA4415V	trastuzumab	0/92	48	44	pCR (23w)	20.17

[a]Study duration is defined as the period from the date of first enrolled patient to cut-off date of primary analysis. The median duration for assessing the primary efficacy endpoints of bevacizumab biosimilars, rituximab biosimilars, and trastuzumab biosimilars was 18 weeks, 23 weeks, and 24 weeks, respectively.

Abbreviations: NA, not available; ORR, objective response rate; pCR, pathologic complete response; PFS, progression-free survival; PFSR, progression-free survival rate; MRCT, multi-regional clinical trial; Early BC, early breast cancer; w, week.

© 2023 Luo X et al. *JAMA Network Open.*

eTable 7. Demographic and clinical characteristics of the included cohort studies (n=10).

First Author	Year	Trial design	Country	Sex(M/F)	Cancer types	Biosimilar	Originator	No. Patients in biosimilar	No. Patients in originator	Primary efficacy endpoints	study duration[a] (month)
**Rituximab**											
Roy et al.[40]	2013	retrospective analysis	India	156/67	diffuse large B-cEll lymphoma	Reditux	Rituximab	72	101	NA	79.07
Bankar et al.[41]	2020	retrospective analysis	India	111/41	diffuse large B-cEll lymphoma	Reditux	Rituximab	92	60	NA	48.67
Deng et al.[42]	2022	retrospective analysis	China	20/13	diffuse large B-cEll lymphoma	HLX-01	Rituximab	15	18	NA	26.30
**Bevacizumab**											
Zhao et al.[43]	2022	retrospective analysis	China	533/413	advanced non-small-cell lung cancer	QL1101	Bevacizumab	551	395	ORR	35.47
**Trastuzumab**											
Bae et al.[44]	2021	retrospective analysis	Korea	NA	Early-stage and metastatic breast cancer	CT-P6	Trastuzumab	125	129	pCR & PFS	69.00
Park et al.[45]	2022	retrospective analysis	Korea	NA	advanced gastric Cancer	CT-P6	Trastuzumab	30	72	NA	120.70
Yang et al.[46]	2022	retrospective analysis	Canada	20/13	early breast cancer w/ neoadjuvant chemotherapy	MYL-1401 O	Trastuzumab	59	77	pCR	12.13
Bernat-Peguera et al.[47]	2022	retrospective analysis	Spain	NA	early breast cancer	CT-P6	Trastuzumab	20	24	NA	48.67
Eser et al.[48]	2023	real-world use/ retrospective analysis	Turkey	NA	early and metastatic breast cancer	MYL-1401 O	Trastuzumab	25	28	pCR and PFS	42.53
Liu et al.[49]	2022	retrospective analysis	China	105/0	early breast cancer	HLX02	Trastuzumab	65	40	NA	9.07

[a]Study duration is defined as the period from the period of participants will be followed and observed to assess the occurrence of specific outcomes.
Abbreviations: NA, not available; ORR, objective response rate; pCR, pathologic complete response; PFS, progression-free survival.

© 2023 Luo X et al. *JAMA Network Open.*

eTable 8. The results of the risk of bias assessment of the RCTs based on the Cochrane Collaboration's tools.

First Author	Year	Random sequence generation (selection bias)	Allocation concealment (selection bias)	Blinding of participants and personnel (performance bias)	Blinding of outcome assessors (performance bias)	Incomplete outcome data (attrition bias)	Selective outcome reporting (reporting bias)	Other potential bias	Overall assessment: risk of bias
**Bevacizumab biosimilars vs Bevacizumab**									
Stroyakovskiy et al.[1]	2022	+	+	+	+	+	+	+	low
Thatcher et al.[2]	2019	+	+	+	+	+	+	+	low
Reinmuth et al.[3]	2019	+	+	+	—	+	+	+	low
Reck et al.[4]	2020	+	+	+	+	—	+	+	low
Yang et al.[5]	2019	+	+	+	+	+	+	+	low
Trukhin et al.[6]	2021	+	+	+	+	+	+	+	low
Rezvani et al.[7]	2020	+	+	+	+	+	+	+	low
Qin et al.[8]	2021	+	+	+	+	+	+	+	low
Shi et al.[9]	2021	+	+	+	—	+	+	+	low
Chu et al[10]	2021	+	+	+	?	—	+	+	unclear
Hengrui[11] (Sponsor)	2022	?	+	+	+	+	+	+	low
Wan et al.[12]	2021	+	+	+	+	+	+	+	low
Syrigos et al.[13]	2021	+	+	+	+	+	+	+	low
Kim et al.[14]	2021	+	+	+	+	—	+	+	low
Socinski et al.[15]	2021	?	?	+	+	+	+	+	unclear
Chen et al.[16]	2022	?	+	+	?	+	+	+	unclear

© 2023 Luo X et al. *JAMA Network Open.*

First Author	Year	Random sequence generation (selection bias)	Allocation concealment (selection bias)	Blinding of participants and personnel (performance bias)	Blinding of outcome assessors (performance bias)	Incomplete outcome data (attrition bias)	Selective outcome reporting (reporting bias)	Other potential bias	Overall assessment: risk of bias
Lu et al.[17]	2023	+	+	+	+	—	+	+	low
Verschraegen et al.[18]	2022	+	+	+	+	+	+	+	low
**Rituximab biosimilars vs Rituximab**									
Kaplanov et al.[19]	2014	+	+	—	—	—	+	+	high
Toogeh et al.[20]	2018	+	+	+	+	+	+	+	low
Jurczak et al.[21]	2017	+	+	+	+	+	+	+	low
Kim et al.[22]	2017	+	+	+	+	+	+	+	low
Ogura et al.[23]	2018	+	+	+	+	+	+	+	low
Poddunaya et al.[24]	2020	+	+	—	—	+	+	+	high
Candelaria et al.[25]	2019	+	+	+	+	+	+	+	low
Sharman et al.[26]	2020	+	+	+	?	+	+	+	low
Niederwieser et al.[27]	2020	+	+	+	+	+	+	+	low
Archigen[28] (Sponor)	2020	?	+	+	+	+	+	+	low
Shi et al.[29]	2020	?	+	+	?	+	+	+	unclear
Song et al.[30]	2021	+	+	+	+	+	+	+	low
**Trastuzumab biosimilars vs Trastuzumab**									
Stebbing et al.[31]	2017	+	+	+	+	+	+	+	low

© 2023 Luo X et al. *JAMA Network Open.*

First Author	Year	Random sequence generation (selection bias)	Allocation concealment (selection bias)	Blinding of participants and personnel (performance bias)	Blinding of outcome assessors (performance bias)	Incomplete outcome data (attrition bias)	Selective outcome reporting (reporting bias)	Other potential bias	Overall assessment: risk of bias
Rugo et al.[32]	2017	+	+	+	+	+	+	+	low
von Minckwitz et al.[33]	2018	+	+	+	+	+	+	+	low
Pivot et al.[34]	2018	+	+	+	?	+	+	+	low
Pegram et al.[35]	2019	+	+	+	+	+	+	+	low
Pivot et al.[36]	2022	+	+	+	?	?	+	+	unclear
Alexeev et al.[37]	2020	+	+	+	+	+	+	+	low
Xu et al.[38]	2021	+	+	+	+	+	+	+	low
Nodehi et al.[39]	2022	?	+	+	+	+	+	+	low

The green, yellow and red color indicates low risk, unclear and high risk of bias, respectively.

© 2023 Luo X et al. *JAMA Network Open.*

eTable 9. The results of the risk of bias assessment of the cohort studies based on the Newcastle-Ottawa Risk of Bias Assessment Tool.

First Author	Year	Representativeness of the exposed cohort	Selection of the non-exposed cohort	Ascertainment of exposure	Demonstration that outcome of interest was not present at baseline	Comparability of cohorts on the basis of the design or analysis	Assessment of outcomes	Was follow-up long enough for outcomes occur	Adequacy of follow up of cohorts	Total scores[a] (risk of bias)
Roy et al.[40]	2013	1	1	1	1	0	1	1	1	7 (low)
Bankar et al.[41]	2020	1	1	1	1	1	1	1	0	7 (low)
Deng et al.[42]	2022	1	1	1	1	1	1	0	0	6 (high)
Zhao et al. [43]	2023	1	1	1	1	2	1	0	0	7 (low)
Bae et al.[44]	2021	1	1	1	1	2	1	1	0	8 (low)
Park et al.[45]	2022	1	1	1	1	1	1	0	0	6 (high)
Yang et al.[46]	2022	1	1	1	1	1	0	0	0	5 (high)
Bernat-Peguera et al.[47]	2022	1	1	1	1	1	1	0	0	6 (high)
Eser et al.[48]	2023	1	1	1	1	2	1	0	0	7 (low)
Liu et al.[49]	2022	1	1	1	1	2	1	0	0	7 (low)

The green, yellow and red colours indicates low risk, some concerns and high risk of bias, respectively; [a]Total scores (risk of bias): scoring from 7-9: high quality; Scoring from 4-6: high risk of bias; Scoring from 0-3: very high risk of bias.

© 2023 Luo X et al. *JAMA Network Open.*

eTable 10. Characteristics of pivotal clinical trials used to support approval of cancer biosimilars by the NMPA, FDA, EMA, and PMDA.

Regulatory Agencies	Brand name	Clinical trial number	Study design	Margin criterion	Cancer types	Biosimilar	Originator	No. Patients in biosimilar	No. Patients in originator	Primary efficacy endpoints (Times)
**Bevacizumab biosimilars**										
FDA/EMA/PMDA	Mvasi/Bevacizumab BS	NCT01966003	equivalence	RR=90%CI (0.67, 1.50)	NSCLC	ABP215	bevacizumab	328	314	ORR (25w)
FDA/EMA/PMDA	Zirabev/Bevacizumab BS	NCT02364999	equivalence	RR=90%CI (0.73, 1.37)	NSCLC	PF-06439535	bevacizumab	358	361	ORR (19w)
FDA/EMA/PMDA	Vegzelma/Bevacizumab BS	NCT03676192	equivalence	RR=90%CI (0.73,1.36); RD=95%CI(-12.5%, 12.5%)	NSCLC	CT-P16	bevacizumab	342	347	ORR (21w)
FDA/EMA/PMDA	Alymsys/Bevacizumab BS	NCT03296163	equivalence	RR=90%CI (0.73,1.36); RD=(-12%, 12%)	NSCLC	MB02	bevacizumab	315	312	ORR (18w)
EMA	Aybintio	NCT02754882	equivalence	RR=90%CI (0.74,1.36); RD=95%CI(-12.5%, 12.5%)	NSCLC	SB8	bevacizumab	379	384	ORR (24w)
EMA	Equidacent (withdrawal)	NCT02810457	equivalence	RR=90%CI (0.73, 1.38); RD=95%CI (-12.21%, 12.21%)	NSCLC	FKB238	bevacizumab	364	367	ORR (30m)
EMA	Abevmy	EudraCT no. 2015-005141-32	equivalence	RR=90%CI (0.73, 1.36); RD=95%CI(-12.5%, 12.5%)	NSCLC	MYL-14020	bevacizumab	337	334	ORR (18w)
NMPA	Dayoutong	NCT02954172	equivalence	RR=90%CI (0.75,1.33)	NSCLC	IBI305	bevacizumab	224	226	ORR (18w)
NMPA	Hanbeitai	NCT03511963	equivalence	RR=90%CI (0.8, 1.25); RD=95%CI (-11%, 15%)	mCRC	HLX04	bevacizumab	340	337	PFSR (36w)
NMPA	Boyounuo	NCT03533127	equivalence	RR=90%CI (0.75, 1.33)	NSCLC	LY01008	bevacizumab	324	325	ORR (18w)
NMPA	Ankeda	NCT03169335	equivalence	RR=90%CI (0.75, 1.33)	NSCLC	QL1101	bevacizumab	269	266	ORR (18w)
NMPA	Airuituo	NCT05169801	equivalence	RR=90%CI (0.75, 1.33)	NSCLC	BP102	bevacizumab	262	255	ORR (18w)
NMPA	Beianting	NCT03196986	equivalence	RR=90%CI (0.75, 1.33)	NSCLC	MIL60	bevacizumab	257	260	ORR (12w)
NMPA	Pubeixi	NCT03329911	equivalence	RR=90%CI (0.75, 1.33)	NSCLC	BAT1706	bevacizumab	325	326	ORR (18w)
NMPA	Puxinting	NCT05427305	equivalence	RR=90%CI (0.75, 1.33)	NSCLC	TAB 008	bevacizumab	277	272	ORR (18w)

© 2023 Luo X et al. *JAMA Network Open.*

**Rituximab biosimilars**										
FDA/EMA/PMDA	Ruxience/Rituximab biosimilar	NCT02213263	equivalence	RD=95%CI (-16%, 16%)	FL	PF-05280587	rituximab	196	198	ORR (27w)
FDA	Truxima	NCT02260804	equivalence	RD=90%CI (-17%, 17%)	FL	CT-P10	rituximab	130	128	ORR (7m)
FDA/EMA	Riabni	NCT02747043	noninferiority	RD=90%CI (-15%, 35.5%)	FL	ABP 798	rituximab	128	128	ORR (28w)
EMA	Truxima	NCT02162771	noninferiority	one-sided 97.5% CI lay on the positive side of the –7% margin	FL	CT-P10	rituximab	70	70	ORR (24w)
PMDA	Rituximab biosimilar	NCT01419665	equivalence	RD=95%CI (-12%, 12%)	FL	GP2013	rituximab	314	315	ORR (21w)
NMPA	Hanlikang	NCT02787239；CTR20150583	equivalence	RD=95%CI (-12%, 12%)	DLBCL	HLX01	rituximab	201	206	ORR (18w)
NMPA	Dabohua	NCT02867566	equivalence	RD=95%CI (-12%, 12%)	DLBCL	IBI301	rituximab	209	210	ORR (18w)
**Trastuzumab biosimilars**										
FDA/EMA/PMDA	Herzuma/Rituximab biosimilar	NCT02162667	equivalence	RD=95%CI (-15%, 15%)	Early BC	CT-P6	trastuzumab	271	278	pCR (30w)
FDA/EMA	Ogivri	NCT02472964	equivalence	RD=95%CI (-15%, 15%)	mBC	MYL-14010	trastuzumab	250	250	ORR (24w)
FDA/EMA/PMDA	Kanjinti/Rituximab biosimilar	NCT01901146/CT 2012-004319-29	equivalence	RD=90%CI (-13%, 13%); RR=90%CI (0.759, 1.318)	Early BC	ABP 980	trastuzumab	364	361	pCR (7w)
FDA/EMA	Ontruzant	NCT02149524	equivalence	RD=95%CI(-13%, 13%); RR=95%CI (0.785, 1.546)	Early BC	SB3	trastuzumab	437	438	pCR (24w)
FDA/EMA/PMDA	Trazimera/Rituximab biosimilar	NCT01989676	equivalence	RR=95%CI (0.80, 1.25)	mBC	PF-05280014	trastuzumab	352	355	ORR (25w)
EMA/NMPA	Hanquyou	NCT03084237; EudraCT 2016-000206-10; CTR20160526	equivalence	RD=95%CI (-13.5%, 13.5%)	mBC	HLX02	trastuzumab	324	325	ORR (24w)

Abbreviations: NA, not available; ORR, objective response rate; pCR, pathologic complete response; PFS, progression-free survival; NSCLC, non-small cell lung cancer; FL, follicular lymphoma; DLBCL, diffuse large B-cell lymphoma; Early BC, early breast cancer; mBC, metastatic breast cancer; FDA, US Food and Drug Administration; EMA, European Medicines Agency; PMDA, Japanese Pharmaceuticals and Medical Devices Agency; NMPA, National Medical Products Administration. RR, risk ratio; RD, risk difference; w, week; m, month.

© 2023 Luo X et al. *JAMA Network Open.*

eTable 11. Subgroup analysis of cancer biosimilars compared with reference drugs derived from randomized clinical trials.

Variables	Number of studies	Model	Heterogeneity	Outcomes	*P* value[a]
			I^2(%)	RR (95% CI)	
**Bevacizumab versus bevacizumab biosimilar**					
**Stage of NSCLC**					
IIIb /IV stage	4	Random	0	0.97 (0.93, 1.01)	0.0969
Others	12	Random	0	0.99 (0.95, 1.04)	
**Country of the sponsor**					
China	7	Random	0	0.98 (0.92, 1.04)	0.165
The United States	3	Random	0	0.96 (0.87, 1.07)	
European Union	3	Random	0	0.91 (0.83, 0.99)	
Others	3	Random	0	1.06 (0.95, 1.18)	
**Number of included patients**					
n≤500	2	Random	0	0.98 (0.83,1.16)	0.953
n>500	14	Random	0	0.97 (0.93,1.01)	
**Rituximab versus rituximab biosimilar**					
**Cancer type**					
Follicular Lymphoma	7	Random	0	1.02 (0.98, 1.06)	0.762
Chronic Lymphocytic Leukemia	1	Random	NA	0.99 (0.81, 1.22)	
Diffuse large B cell lymphoma	3	Random	91	1.07 (0.93, 1.24)	
Indolent NHL	1	Random	NA	1.03 (0.93, 1.24)	
**Country of the sponsor**					
China	2	Random	52	0.99 (0.93, 1.04)	0.345
The United States	2	Random	0	1.09 (0.99, 1.19)	
European Union	3	Random	0	1.00 (0.95, 1.05)	
Others	5	Random	0	1.02 (0.96,1.08)	
**Number of included patients**					
n≤300	7	Random	0	1.04 (0.98, 1.09)	0.137
n>300	5	Random	4	0.99 (0.96, 1.02)	
**Trastuzumab versus trastuzumab biosimilar**					
**Cancer stage**					
Early BC	5	Random	68	1.07 (0.91, 1.24)	0.522
mBC	5	Random	0	1.01 (0.95, 1.07)	
**Country of the sponsor**					
China	1	Random	NA	1.04 (0.96, 1.10)	0.0213
The United States	2	Random	0	1.22 (1.06, 1.41)	
European Union	2	Random	66	1.07 (0.91, 1.27)	
Others	4	Random	19	0.98 (0.90,1.06)	
**Number of included patients**					
n≤600	5	Random	45	1.02 (0.94, 1.10)	0.440
n>600	5	Random	45	1.09 (0.92, 1.29)	

[a]*P* value indicates a comparison between different subgroups. RR, response rate; CI, confidence interval. FL, follicular lymphoma; CLL, chronic lymphocytic leukemia; DLBCL, diffuse large B cell lymphoma; Indolent NHL, indolent non-Hodgkin lymphomas; Early BC, early breast cancer; mBC, metastatic breast cancer; NSCLC, non-small cell lung cancer; NA, not available.

© 2023 Luo X et al. *JAMA Network Open.*

eTable 12. Pooled analysis of efficacy (secondary end points), safety, and immunogenicity of cancer biosimilars derived from randomized clinical trials.

Outcome and cancer subgroup	RCTs, No.	Sample size, No.		Test of heterogeneity		Results of meta-analysis, effect estimates (95% CI)	*P* value[a]
		Biosimilar	Reference	I^2, %	*P* value		
**Bevacizumab biosimilar vs. Bevacizumab**							
**Secondary efficacy endpoints**							
OS (HR)	11	3044	3011	21	0.25	1.02 (0.94, 1.11)	0.51
PFS (HR)	10	3239	3239	10	0.35	1.03 (0.97, 1.10)	0.30
**Safety outcome**							
SAE	16	4692	4567	0	0.99	1.04 (0.97, 1.11)	0.24
Grade⩾3 AEs	14	4071	3958	0	0.84	0.98 (0.95, 1.00)	0.08
Death	15	4457	4345	0	1.00	1.04 (0.97, 1.12)	0.25
Neutropenia (Grade⩾3 AEs)	11	3504	3491	17	0.28	0.97 (0.84, 1.12	0.66
Thrombocytopenia (Grade⩾3 AEs)	10	3125	3107	0	0.93	1.09 (0.86, 1.37)	0.48
**Immunogenicity outcome**							
ADA	13	3946	2884	0	0.63	1.04 (0.88, 1.25)	0.63
Nab	5	1591	1532	0	0.82	0.78 (0.45, 1.34)	0.36
**Rituximab biosimilar vs. Rituximab**							
**Secondary efficacy endpoints**							
OS (HR)	1	311	315	NA	NA	0.77 (0.45, 1.33)	0.34
PFS (HR)	3	646	649	42	0.18	1.16 (0.87, 1.54)	0.33
**Safety outcome**							
SAE	9	1541	1549	0	0.89	1.11 (0.98, 1.27)	0.11
Grade⩾3 AEs	5	870	878	0	0.82	1.02 (0.96, 1.08)	0.61
Death	8	1471	1479	0	0.92	0.88 (0.64, 1.21)	0.45
Neutropenia (Grade⩾3 AEs)	9	1260	1261	0	0.97	1.07 (0.88, 1.30)	0.48
Thrombocytopenia (Grade⩾3 AEs)	3	485	486	47	0.15	2.20 (0.48, 10.11)	0.31
**Immunogenicity outcome**							
ADA	5	996	1005	0	0.58	1.06 (0.77, 1.46)	0.72
Nab	1	157	158	NA	NA	4.71 (0.21, 105.24)	0.33
**Trastuzumab biosimilar vs. Trastuzumab**							
**Secondary efficacy endpoints**							
OS (HR)	3	926	930	4	0.35	0.84 (0.65, 1.07)	0.15
PFS (HR)	3	926	930	0	0.64	0.89 (0.76, 1.04)	0.15
**Safety outcome**							
SAE	6	1789	1792	54	0.05	0.94 (0.73, 1.21)	0.63
Grade⩾3 AEs	5	1562	1570	0	0.64	0.99 (0.94, 1.05)	0.84
Death	6	1729	1729	25	0.24	0.79 (0.54, 1.16)	0.23
Neutropenia (Grade⩾3 AEs)	6	1789	1792	0	0.89	0.96 (0.74, 1.23)	0.74
Thrombocytopenia (Grade⩾3 AEs)	3	1039	1043	0	0.84	1.95 (0.34, 11.20)	0.46
**Immunogenicity outcome**							
ADA	5	1626	1625	0	0.77	1.12 (0.58, 2.14)	0.74
Nab	2	439	435	0	0.80	0.82 (0.25, 2.66)	0.74

[a]*P* value indicates a comparison between different subgroups.
Abbreviations: NA, not available; ORR, objective response rate; PFS, progression-free survival; HR, hazard ratio; OS, overall survival; SAE, serious adverse event; ADA, anti-drug antibody; Nab, neutralizing antibody; CI, confidence interval.

© 2023 Luo X et al. *JAMA Network Open.*

eTable 13. Pooled analysis of efficacy, safety, and immunogenicity of cancer biosimilars derived from cohort studies.

Outcome and cancer subgroup	cohort studies, No.	Sample size, No.		Test of heterogeneity		Results of meta-analysis, effect estimates (95% CI)	*P* value[a]
		Biosimilar	Reference	I^2, %	*P* value		
**Bevacizumab biosimilar vs. Bevacizumab**							
**Efficacy endpoints**							
ORR	1	551	395	NA	NA	0.93 (0.77, 1.14)	0.50
Safety outcome							
Grade⩾3 AEs	1	551	395	NA	NA	1.14 (0.88, 1.45)	0.34
Neutropenia (Grade⩾3 AEs)	1	551	395	NA	NA	1.14 (0.80, 1.64)	0.47
Thrombocytopenia (Grade⩾3 AEs)	1	551	395	NA	NA	1.58 (0.55, 4.50)	0.39
**Rituximab biosimilar vs. Rituximab**							
Efficacy endpoints							
ORR	3	179	179	0	0.25	1.00 (0.92, 1.01)	0.88
Safety outcome							
Grade⩾3 AEs	2	87	119	0	0.66	0.78 (0.51, 1.18)	0.23
Neutropenia (Grade⩾3 AEs)	2	87	119	0	0.98	0.90 (0.49, 1.64)	0.75
Death	2	164	161	0	0.98	1.32 (0.43, 4.07)	0.63
**Trastuzumab biosimilar vs. Trastuzumab**							
Efficacy endpoints							
ORR	3	229	409	0	0.72	1.00 (0.87, 1.16)	0.96
pCR	5	294	298	0	0.91	1.04 [0.93; 1.17]	0.50
Safety outcome							
Grade⩾3 AEs	3	141	285	41	0.19	1.02 (0.81, 1.30)	0.86
Neutropenia (Grade⩾3 AEs)	1	65	40	NA	NA	1.86 (0.08, 44.67)	0.70
Thrombocytopenia (Grade⩾3 AEs)	1	65	40	NA	NA	1.53 (0.31, 7.56)	0.60

[a]*P* value indicates a comparison between different subgroups.

Abbreviations: NA, not available; ORR, objective response rate; pCR, pathologic complete response; PFS, progression-free survival; OS, overall survival; OS, overall survival; SAE, serious adverse event; CI, confidence interval.

© 2023 Luo X et al. *JAMA Network Open.*

eTable 14. The weighted mean price of rituximab, bevacizumab, trastuzumab, and their biosimilars (per unit) between 2015 and 2022.

	Bevacizumab（$/mg）			Rituximab（$/mg）			Trastuzumab（$/mg）		
**Year**	**Biosimilar**	**Reference**	**B/R[a] (%)**	**Biosimilar**	**Reference**	**B/R[a] (%)**	**Biosimilar**	**Reference**	**B/R[a] (%)**
2022	1.63	2.21	74	1.55	2.23	69	1.66	1.85	90
2021	1.67	2.23	75	1.59	2.35	68	1.68	1.86	90
2020	1.76	2.25	78	1.84	2.36	78	1.69	1.87	90
2019	1.79	3.03	59	2.37	2.47	96	NA	2.59	NA
2018	NA	3.09	NA	NA	3.40	NA	NA	2.64	NA
2017	NA	5.61	NA	NA	4.65	NA	NA	4.39	NA
2016	NA	8.64	NA	NA	5.93	NA	NA	8.42	NA
2015	NA	8.66	NA	NA	6.12	NA	NA	8.22	NA

Abbreviations：B, biosimilar; R, reference; [a]B/R is defined as the price ratio of the biosimilar to the reference drug; NA, not available.

© 2023 Luo X et al. *JAMA Network Open.*

eTable 15. Trends in the uptake rate of rituximab, bevacizumab, and trastuzumab biosimilars vs reference drugs after market entry as of December 2022

Time frames of market entry	Date of market entry	Biosimilar, mg	Reference, mg	Overall[a], mg	B/O[b] (%)
Bevacizumab biosimilar vs. Bevacizumab					
M_{1-3}	2019_{Q4}	7186	493757	500943	1
M_{4-6}	2020_{Q1}	36002	384235	420237	9
M_{7-9}	2020_{Q2}	226007	472741	698748	32
M_{10-12}	2020_{Q3}	377474	502235	879709	43
M_{13-15}	2020_{Q4}	485623	409156	894779	54
M_{16-18}	2021_{Q1}	837009	450771	1287780	65
M_{19-21}	2021_{Q2}	940418	383222	1323640	71
M_{22-24}	2021_{Q3}	1181508	394229	1575737	75
M_{25-27}	2021_{Q4}	1153490	351618	1505108	77
M_{28-30}	2022_{Q1}	1185853	294534	1480387	80
M_{31-33}	2022_{Q2}	1376403	269110	1645513	84
M_{34-36}	2022_{Q3}	1575914	308869	1884783	84
M_{37-39}	2022_{Q4}	1279435	233691	1513126	85
Rituximab biosimilar vs. Rituximab					
M_{1-3}	2019_{Q2}	717	385942	386659	0
M_{4-6}	2019_{Q3}	15498	373160	388658	4
M_{7-9}	2019_{Q4}	23788	303334	327122	7
M_{10-12}	2020_{Q1}	43346	202112	245458	18
M_{13-15}	2020_{Q2}	100002	276208	376210	27
M_{16-18}	2020_{Q3}	158286	229785	388071	41
M_{19-21}	2020_{Q4}	176735	164032	340767	52
M_{22-24}	2021_{Q1}	266696	230927	497623	54
M_{25-27}	2021_{Q2}	320382	205045	525427	61
M_{28-30}	2021_{Q3}	410411	243848	654259	63
M_{31-33}	2021_{Q4}	394913	188240	583153	68
M_{34-36}	2022_{Q1}	383601	180160	563761	68
M_{37-39}	2022_{Q2}	434918	155895	590813	74
M_{40-42}	2022_{Q3}	504666	157110	661776	76
M_{43-45}	2022_{Q4}	410409	121447	531856	77
Trastuzumab biosimilar vs. Trastuzumab					
M_{1-3}	2020_{Q3}	935	200390	201325	0
M_{4-6}	2020_{Q4}	18379	197674	216053	9
M_{7-9}	2021_{Q1}	83070	254566	337636	25
M_{10-12}	2021_{Q2}	120180	263731	383911	31

© 2023 Luo X et al. *JAMA Network Open.*

M_{13-15}	2021_{Q3}	176843	256501	433344	41
M_{16-18}	2021_{Q4}	196913	248212	445125	44
M_{19-21}	2022_{Q1}	215587	226398	441985	49
M_{22-24}	2022_{Q2}	248326	224295	472621	53
M_{25-27}	2022_{Q3}	303579	220637	524216	58
M_{28-30}	2022_{Q4}	279375	202689	482064	58

M_n, Month from launch; Q, Quarter; B, biosimilar; O, [a]overall, overall is defined as the sum of sales volume of biosimilars and reference drugs quarterly; [b]B/O is defined as the ratio of sales volume of biosimilars to overall quarterly.

© 2023 Luo X et al. *JAMA Network Open.*

eTable 16. Uptake rates of cancer biosimilars vs reference drugs in the first year, second year, and latest time on the market

Biosimilars	First year after launching	Second year after launching	Latest year (2022)
Bevacizumab	26%	68%	83%
Rituximab	6%	44%	74%
Trastuzumab	20%	47%	54%

The annual uptake rate for biosimilars is calculated by dividing the one-year sales of biosimilars by the one-year sales of their reference drugs.

© 2023 Luo X et al. *JAMA Network Open.*

eTable 17. Reimbursable cancer indications for the biosimilars and reference drugs of bevacizumab, rituximab, and trastuzumab in the National Reimbursement Drug List in China (As of July 1, 2023).

Originator	Reimbursable indications
Bevacizumab	1. Metastatic colorectal cancer<br>2. Advanced, metastatic, or recurrent non-small non-squamous cell lung cancer<br>3. Recurrent Glioblastoma<br>4. Hepatocellular carcinoma 5. Epithelial ovarian, fallopian tube, or primary peritoneal cancer<br>6. Cervical cancer
Rituximab	1. Non-Hodgkin' s lymphoma 2. Chronic lymphocytic leukemia
Trastuzumab	1. HER2-overexpressing metastatic breast cancer (MBC);<br>2. HER2-overexpressing early breast cancer (Adjuvant and neoadjuvant therapy, paid for no more than 12 months)[a];<br>3. HER2-overexpressing metastatic gastric cancer[b].

[a]HER2-overexpressing early breast cancer indication is included in NRDL, but only paid for no more than 12 months;
[b]Trastuzumab is approved for HER2-overexpressing metastatic gastric or gastroesophageal junction enocarcinoma, but reimbursement only paid for HER2-overexpressing metastatic gastric cancer.

© 2023 Luo X et al. *JAMA Network Open.*

eTable 18. Comparison the approved indications of cancer biosimilars vs reference drugs by the NMPA, FDA, EMA, and PMDA (As of July 2023).

Number	NMPA	FDA	EMA	PMDA
**Bevacizumab and Bevacizumab biosimilars**				
Reference drug	**Avastin**<br>**(Roche)**<br>1. Metastatic colorectal cancer<br>2. Advanced, metastatic, or recurrent non-small non-squamous cell lung cancer<br>3. Recurrent Glioblastoma<br>4. Hepatocellular carcinoma (HCC)<br>5. Epithelial ovarian, fallopian tube, or primary peritoneal cancer<br>6. Cervical cancer	**Avastin**<br>**(Roche)**<br>1. Metastatic colorectal cancer<br>2. Unresectable, locally advanced, recurrent or metastatic non-squamous non-small cell lung cancer<br>3. Recurrent glioblastoma<br>4. Hepatocellular carcinoma (HCC)<br>5. Epithelial ovarian, fallopian tube, or primary peritoneal cancer<br>6. Persistent, recurrent, or metastatic cervical cancer<br>7. Metastatic renal cell carcinoma	**Avastin**<br>**(Roche)**<br>1. Metastatic carcinoma of the colon or rectum<br>2. Unresectable advanced, metastatic or recurrent nono-squamous non-small cell lung cancer<br>3. Epithelial ovarian, fallopian tube, or primary peritoneal cancer<br>4. Persistent, recurrent, or metastatic carcinoma of the cervix<br>5. Advanced and/or metastatic renal cell cancer<br>6. Metastatic breast cancer	**Avastin**<br>**(Roche)**<br>1. Unresectable advanced or recurrent colorectal cancer<br>2. Unresectable advanced or recurrent non-small cell lung cancer<br>3. Malignant Glioblastoma<br>4. Unresectable hepatocellular carcinoma (HCC)<br>5. Ovarian cancer<br>6. Advanced or recurrent cervical cancer<br>7. Inoperable or recurrent breast cancer
1	**Hanbeitai**<br>**(Henlius)**<br>**[HLX04]**<br>1. Metastatic colorectal cancer<br>2. Advanced, metastatic, or recurrent non-small non-squamous cell lung cancer<br>3. Recurrent Glioblastoma<br>4. Hepatocellular carcinoma<br>5. Epithelial ovarian, fallopian tube, or primary peritoneal cancer<br>6. Cervical cancer	**Zirabev**<br>**(Pfizer)**<br>**[PF-06439535]**<br>1. Metastatic colorectal cancer<br>2. Unresectable, locally advanced, recurrent or metastatic non-squamous non-small cell lung cancer<br>3. Recurrent glioblastoma<br>4. Hepatocellular carcinoma (HCC)<br>5. Epithelial ovarian, fallopian tube, or primary peritoneal cancer<br>6. Persistent, recurrent, or metastatic cervical cancer<br>7. Metastatic renal cell carcinoma	**Zirabev**<br>**(Pfizer)**<br>**[PF-06439535]**<br>1. Metastatic carcinoma of the colon or rectum<br>2. Unresectable advanced, metastatic or recurrent nono-squamous non-small cell lung cancer<br>3. Epithelial ovarian, fallopian tube, or primary peritoneal cancer<br>4. Persistent, recurrent, or metastatic carcinoma of the cervix<br>5. Advanced and/or metastatic renal cell cancer<br>6. Metastatic breast cancer	**Bevacizumab biosimilar**<br>**(Pfizer)**<br>**[PF-06439535]**<br>1. Unresectable advanced or recurrent colorectal cancer<br>2. Unresectable advanced or recurrent non-small cell lung cancer<br>3. Malignant Glioblastoma<br>4. Unresectable hepatocellular carcinoma (HCC)<br>5. Ovarian cancer<br>6. Advanced or recurrent cervical cancer<br>7. Inoperable or recurrent breast cancer
2	**Puxinting**<br>**(TOT Biopharm)**<br>**[TAB 008]**<br>1. Metastatic colorectal cancer<br>2. Advanced, metastatic, or recurrent non-small	**Alymsys**<br>**(Amneal)**<br>**[MB02]**<br>1. Metastatic colorectal cancer<br>2. Unresectable, locally advanced, recurrent or metastatic non-squamous non-small cell lung cancer	**Alymsys**<br>**(Mabxience)**<br>**[MB02]**<br>1. Metastatic carcinoma of the colon or rectum<br>2. Unresectable advanced, metastatic or recurrent nono-squamous non-small cell lung cancer	**Bevacizumab biosimilar**<br>**(Amgen/Daiichi-Sankyo)**<br>**[ABP215]**<br>1. Unresectable advanced or recurrent colorectal cancer<br>2. Unresectable advanced or recurrent non-small cell lung cancer

© 2023 Luo X et al. *JAMA Network Open.*

Number	NMPA	FDA	EMA	PMDA
	non-squamous cell lung cancer<br>3. Recurrent Glioblastoma<br>4. Hepatocellular carcinoma<br>5. Epithelial ovarian, fallopian tube, or primary peritoneal cancer<br>6. Cervical cancer	3. Recurrent glioblastoma<br>4. Hepatocellular carcinoma (HCC)<br>5. Epithelial ovarian, fallopian tube, or primary peritoneal cancer<br>6. Persistent, recurrent, or metastatic cervical cancer<br>7. Metastatic renal cell carcinoma	3. Epithelial ovarian, fallopian tube, or primary peritoneal cancer.<br>4. Persistent, recurrent, or metastatic carcinoma of the cervix<br>5. Advanced and/or metastatic renal cell cancer<br>6. Metastatic breast cancer	3. Malignant Glioblastoma<br>4. Unresectable hepatocellular carcinoma (HCC)<br>5. Ovarian cancer<br>6. Advanced or recurrent cervical cancer<br>7. Inoperable or recurrent breast cancer
3	**Beianting**<br>**(Betta/ Mabwork)**<br>**[MIL60]**<br>1. Metastatic colorectal cancer<br>2. Advanced, metastatic, or recurrent non-small non-squamous cell lung cancer<br>3. Recurrent Glioblastoma<br>4. Hepatocellular carcinoma<br>5. Epithelial ovarian, fallopian tube, or primary peritoneal cancer<br>6. Cervical cancer	**Vegzelma**<br>**(Celltrion)**<br>**[CT-P16]**<br>1. Metastatic colorectal cancer<br>2. Unresectable, locally advanced, recurrent or metastatic non-squamous non-small cell lung cancer<br>3. Recurrent glioblastoma<br>4. Hepatocellular carcinoma (HCC)<br>5. Epithelial ovarian, fallopian tube, or primary peritoneal cancer<br>6. Persistent, recurrent, or metastatic cervical cancer<br>7. Metastatic renal cell carcinoma	**Vegzelma**<br>**(Celltrion)**<br>**[CT-P16]**<br>1. Metastatic carcinoma of the colon or rectum<br>2. Unresectable advanced, metastatic or recurrent nono-squamous non-small cell lung cancer<br>3. Epithelial ovarian, fallopian tube, or primary peritoneal cancer<br>4. Persistent, recurrent, or metastatic carcinoma of the cervix<br>5. Advanced and/or metastatic renal cell cancer<br>6. Metastatic breast cancer	**Bevacizumab biosimilar**<br>**(Nipponkayaku/ Celltrion)**<br>**[CT-P16]**<br>1. Unresectable advanced or recurrent colorectal cancer<br>2. Unresectable advanced or recurrent non-small cell lung cancer<br>3. Malignant Glioblastoma<br>4. Unresectable hepatocellular carcinoma (HCC)<br>5. Ovarian cancer<br>6. Advanced or recurrent cervical cancer<br>7. Inoperable or recurrent breast cancer
4	**Pubeixi**<br>**(Bio-Thera)**<br>**[BAT1706]**<br>1. Metastatic colorectal cancer<br>2. Advanced, metastatic, or recurrent non-small non-squamous cell lung cancer<br>3. Recurrent Glioblastoma<br>4. Hepatocellular carcinoma<br>5. Epithelial ovarian, fallopian tube, or primary peritoneal cancer<br>6. Cervical cancer	**Mvasi**<br>**(Amgen)**<br>**[ABP215]**<br>1. Metastatic colorectal cancer<br>2. Unresectable, locally advanced, recurrent or metastatic non-squamous non-small cell lung cancer<br>3. Recurrent glioblastoma<br>4. Hepatocellular carcinoma (HCC)<br>5. Epithelial ovarian, fallopian tube, or primary peritoneal cancer<br>6. Persistent, recurrent, or metastatic cervical cancer<br>7. Metastatic renal cell carcinoma	**Mvasi**<br>**(Amgen)**<br>**[ABP215]**<br>1. Metastatic carcinoma of the colon or rectum<br>2. Unresectable advanced, metastatic or recurrent nono-squamous non-small cell lung cancer<br>3. Epithelial ovarian, fallopian tube, or primary peritoneal cancer<br>4. Persistent, recurrent, or metastatic carcinoma of the cervix<br>5. Advanced and/or metastatic renal cell cancer<br>6. Metastatic breast cancer	**Bevacizumab biosimilar**<br>**(Nichiiko)**<br>**[MB02]**<br>1. Unresectable advanced or recurrent colorectal cancer<br>2. Unresectable advanced or recurrent non-small cell lung cancer<br>3. Malignant Glioblastoma<br>4. Unresectable hepatocellular carcinoma (HCC)<br>5. Ovarian cancer<br>6. Advanced or recurrent cervical cancer<br>7. Inoperable or recurrent breast cancer

© 2023 Luo X et al. *JAMA Network Open.*

Number	NMPA	FDA	EMA	PMDA
5	**Airuituo**<br>**(Hengrui)**<br>**[BP102]**<br>1. Metastatic colorectal cancer<br>2. Advanced, metastatic, or recurrent non-small non-squamous cell lung cancer<br>3. Recurrent Glioblastoma<br>4. Hepatocellular carcinoma<br>5. Epithelial ovarian, fallopian tube, or primary peritoneal cancer<br>6. Cervical cancer		**Abevmy**<br>**(Mylan)**<br>**[MYL-1402O]**<br>1. Metastatic carcinoma of the colon or rectum<br>2. Unresectable advanced, metastatic or recurrent nono-squamous non-small cell lung cancer<br>3. Epithelial ovarian, fallopian tube, or primary peritoneal cancer<br>4. Persistent, recurrent, or metastatic carcinoma of the cervix<br>5. Advanced and/or metastatic renal cell cancer<br>6. Metastatic breast cancer	
6	**Boyounuo**<br>**(Luye)**<br>**[LY01008]**<br>1. Metastatic colorectal cancer<br>2. Advanced, metastatic, or recurrent non-small non-squamous cell lung cancer<br>3. Recurrent Glioblastoma<br>4. Hepatocellular carcinoma<br>5. Epithelial ovarian, fallopian tube, or primary peritoneal cancer<br>6. Cervical cancer		**Aybintio**<br>**(Samsung)**<br>**[SB8]**<br>1. Metastatic carcinoma of the colon or rectum<br>2. Unresectable advanced, metastatic or recurrent nono-squamous non-small cell lung cancer<br>3. Epithelial ovarian, fallopian tube, or primary peritoneal cancer<br>4. Persistent, recurrent, or metastatic carcinoma of the cervix<br>5. Advanced and/or metastatic renal cell cancer<br>6. Metastatic breast cancer	

© 2023 Luo X et al. *JAMA Network Open.*

Number	NMPA	FDA	EMA	PMDA
7	**Dayoutong**<br>**(Innovent）**<br>**[IBI305]**<br>1. Metastatic colorectal cancer<br>2. Advanced, metastatic, or recurrent non-small non-squamous cell lung cancer<br>3. Recurrent Glioblastoma<br>4. Hepatocellular carcinoma<br>5. Epithelial ovarian, fallopian tube, or primary peritoneal cancer<br>6. Cervical cancer		WITHDRAWAL<br>**Equidacent**<br>**(Centus)**<br>**[FKB-238]**<br>1. Metastatic carcinoma of the colon or rectum<br>2. Unresectable advanced, metastatic or recurrent no-squamous non-small cell lung cancer<br>3. Epithelial ovarian, fallopian tube, or primary peritoneal cancer<br>4. Persistent, recurrent, or metastatic carcinoma of the cervix<br>5. Advanced and/or metastatic renal cell cancer<br>6. Metastatic breast cancer	
8	**Ankeda**<br>**(Qilu)**<br>**[QL1101]**<br>1. Metastatic colorectal cancer<br>2. Advanced, metastatic, or recurrent non-small non-squamous cell lung cancer<br>3. Recurrent Glioblastoma<br>4. Hepatocellular carcinoma<br>5. Epithelial ovarian, fallopian tube, or primary peritoneal cancer<br>6. Cervical cancer			
**Rituximab versus rituximab biosimilar**				

© 2023 Luo X et al. *JAMA Network Open.*

Number	NMPA	FDA	EMA	PMDA
reference drug	**MabThera (Roche)**<br>1. Non-Hodgkin' s lymphoma (NHL)<br>2. Chronic lymphocytic leukemia (CLL)	**Rituxan (Roche)**<br>1. Non-Hodgkin' s lymphoma (NHL)<br>2. Chronic lymphocytic leukemia (CLL)<br>3. Pediatric mature B-cell NHL, diffuse large B-cell lymphoma (DLBCL), Burkitt lymphoma (BL), Burkitt-like lymphoma (BLL) or mature B-cell acute leukemia (B-AL)	**MabThera (Roche)**<br>1. Non-Hodgkin's lymphoma (NHL)<br>2. Chronic lymphocytic leukaemia (CLL)<br>3. Pediatric advanced stage CD20 positive diffuse large B-cell lymphoma (DLBCL), Burkitt lymphoma (BL)/Burkitt leukaemia (mature B-cell acute leukaemia) (BAL) or Burkitt-like lymphoma (BLL)	**Rituxan (Zenyaku)**<br>1. Non-Hodgkin's lymphoma (NHL)<br>2. Chronic lymphocytic leukaemia (CLL)<br>3. B-cell lymphoproliferative disease under immunosuppressive conditions
1	**Dabohua (Innovent) [IBI301]**<br>1. Non-Hodgkin' s lymphoma (NHL)<br>2.Chronic lymphocytic leukemia (CLL)	**Riabni (Amgen) [ABP 798]**<br>1. Non-Hodgkin' s lymphoma (NHL)<br>2. Chronic lymphocytic leukemia (CLL)<br>3. Pediatric mature B-cell NHL, diffuse large B-cell lymphoma (DLBCL), Burkitt lymphoma (BL), Burkitt-like lymphoma (BLL) or mature B-cell acute leukemia (B-AL)	**Rixathon (Sandoz) [GP2013]**<br>1. Non-Hodgkin's lymphoma (NHL)<br>2. Chronic lymphocytic leukaemia (CLL)<br>3. Pediatric advanced stage CD20 positive diffuse large B-cell lymphoma (DLBCL), Burkitt lymphoma (BL)/Burkitt leukaemia (mature B-cell acute leukaemia) (BAL) or Burkitt-like lymphoma (BLL)	**Rituximab biosimilar-1 (Sandoz/Kyowa) [GP13-301]**<br>1. Non-Hodgkin's lymphoma (NHL)<br>2.Chronic lymphocytic leukaemia (CLL)<br>3. B-cell lymphoproliferative disease under immunosuppressive conditions
2	**Hanlikang (Henlius) [HLX01]**<br>1. Non-Hodgkin' s lymphoma (NHL)<br>2. Chronic lymphocytic leukemia (CLL)	**Ruxience (Pfizer) [PF-05280586]**<br>1. Non-Hodgkin' s lymphoma (NHL)<br>2. Chronic lymphocytic leukemia (CLL)<br>3. Pediatric mature B-cell NHL, diffuse large B-cell lymphoma (DLBCL), Burkitt lymphoma (BL), Burkitt-like lymphoma (BLL) or mature B-cell acute leukemia (B-AL)	**Ruxience (Pfizer) [PF-05280586]**<br>1. Non-Hodgkin's lymphoma (NHL)<br>2. Chronic lymphocytic leukaemia (CLL)<br>3. Pediatric advanced stage CD20 positive diffuse large B-cell lymphoma (DLBCL), Burkitt lymphoma (BL)/Burkitt leukaemia (mature B-cell acute leukaemia) (BAL) or Burkitt-like lymphoma (BLL)	**Rituximab biosimilar-2 (Pfizer) [PF-05280586]**<br>1. Non-Hodgkin's lymphoma (NHL)<br>2.Chronic lymphocytic leukaemia (CLL)<br>3. B-cell lymphoproliferative disease under immunosuppressive conditions
3		**Truxima (Celltrion) [CT-P10]**<br>1. Non-Hodgkin' s lymphoma (NHL)<br>2. Chronic lymphocytic leukemia (CLL)<br>3. Pediatric mature B-cell NHL, diffuse large B-cell lymphoma (DLBCL), Burkitt lymphoma (BL), Burkitt-like lymphoma (BLL) or mature B-cell acute leukemia (B-AL)	**Truxima (Celltrion) [CT-P10]**<br>1. Non-Hodgkin's lymphoma (NHL)<br>2. Chronic lymphocytic leukaemia (CLL)<br>3. Pediatric advanced stage CD20 positive diffuse large B-cell lymphoma (DLBCL), Burkitt lymphoma (BL)/Burkitt leukaemia (mature B-cell acute leukaemia) (BAL) or Burkitt-like lymphoma (BLL)	

© 2023 Luo X et al. *JAMA Network Open.*

Number	NMPA	FDA	EMA	PMDA
**Trastuzumab versus trastuzumab biosimilar**				
Reference drug	**Herceptin (Roche)**<br>1.HER2-overexpressing metastatic breast cancer (MBC)<br>2.HER2-overexpressing early breast cancer (EBC)<br>3.HER2-overexpressing metastatic gastric or gastroesophageal junction enocarcinoma	**Herceptin (Roche)**<br>1.HER2-overexpressing metastatic breast cancer (MBC)<br>2.Adjuvant therapy of HER2-overexpressin breast cancer<br>3.HER2-overexpressing metastatic gastric or gastroesophageal junction enocarcinoma	**Herceptin (Roche)**<br>1. HER2-positive metastatic breast cancer (MBC)<br>2.HER2-positive early breast cancer (EBC)<br>3.HER2-positive metastatic adenocarcinoma of the stomach or gastroesophageal junction	**Herceptin (Chugai)**<br>1. HER2-positive breast cancer<br>2. HER2-positive unresectable advanced or recurrences of gastric cancer<br>3. HER2-positive unresectable advanced recurrent salivary gland cancer<br>4. HER2-positive unresectable advanced and recurred colorectal cancer
1	**Hanquyou (Henlius) [HLX02]**<br>1. HER2-overexpressing metastatic breast cancer (MBC)<br>2. HER2-overexpressing early breast cancer (EBC)<br>3. HER2-overexpressing metastatic gastric or gastroesophageal junction enocarcinoma	**Kanjinti (Amgen) [ABP 980]**<br>1. HER2-overexpressing metastatic breast cancer (MBC)<br>2. Adjuvant therapy of HER2-overexpressin breast cancer<br>3. HER2-overexpressing metastatic gastric or gastroesophageal junction enocarcinoma	**Kanjinti (Amgen) [ABP 980]**<br>1. HER2-positive metastatic breast cancer (MBC)<br>2. HER2-positive early breast cancer (EBC)<br>3.HER2-positive metastatic adenocarcinoma of the stomach or gastroesophageal junction	**Trastuzumab biosimilar-2 (Daiichi-Sankyo/AMGEN) [ABP 980]**<br>1. HER2-positive breast cancer<br>2. HER2-positive unresectable advanced or recurrences of gastric cancer<br>3. HER2-positive unresectable advanced recurrent salivary gland cancer<br>4. HER2-positive unresectable advanced and recurred colorectal cancer
2		**Trazimera (Pfizer) [PF-05280014]**<br>1. HER2-overexpressing metastatic breast cancer (MBC)<br>2. Adjuvant therapy of HER2-overexpressin breast cancer<br>3. HER2-overexpressing metastatic gastric or gastroesophageal junction enocarcinoma	**Trazimera (Pfizer) [PF-05280014]**<br>1. HER2-positive metastatic breast cancer (MBC)<br>2. HER2-positive early breast cancer (EBC)<br>3. HER2-positive metastatic adenocarcinoma of the stomach or gastroesophageal junction	**Trastuzumab biosimilar-3 (Pfizer) [PF-05280014]**<br>1. HER2-positive breast cancer<br>2. HER2-positive unresectable advanced or recurrences of gastric cancer<br>3. HER2-positive unresectable advanced recurrent salivary gland cancer<br>4. HER2-positive unresectable advanced and recurred colorectal cancer

© 2023 Luo X et al. *JAMA Network Open.*

Number	NMPA	FDA	EMA	PMDA
3		**Ontruzant (Samsung) [SB3]**<br>1. HER2-overexpressing metastatic breast cancer (MBC)<br>2. Adjuvant therapy of HER2-overexpressin breast cancer<br>3. HER2-overexpressing metastatic gastric or gastroesophageal junction enocarcinoma	**Ontruzant (Samsung) [SB3]**<br>1. HER2-positive metastatic breast cancer (MBC)<br>2. HER2-positive early breast cancer (EBC)<br>3. HER2-positive metastatic adenocarcinoma of the stomach or gastroesophageal junction	**Trastuzumab biosimilar-1 (Celltrion) [CT-P6]**<br>1. HER2-positive breast cancer<br>2. HER2-positive unresectable advanced or recurrences of gastric cancer<br>3. HER2-positive unresectable advanced recurrent salivary gland cancer<br>4. HER2-positive unresectable advanced and recurred colorectal cancer
4		**Herzuma (Celltrion) [CT-P6]**<br>1. HER2-overexpressing metastatic breast cancer (MBC)<br>2. Adjuvant therapy of HER2-overexpressin breast cancer<br>3. HER2-overexpressing metastatic gastric or gastroesophageal junction enocarcinoma	**Herzuma (Celltrion) [CT-P6]**<br>1. HER2-positive metastatic breast cancer (MBC)<br>2. HER2-positive early breast cancer (EBC)<br>3. HER2-positive metastatic adenocarcinoma of the stomach or gastroesophageal junction	
5		**Ogivri (Viatris) [MYL-1401O]**<br>1. HER2-overexpressing metastatic breast cancer (MBC)<br>2. Adjuvant therapy of HER2-overexpressin breast cancer<br>3. HER2-overexpressing metastatic gastric or gastroesophageal junction enocarcinoma	**Ogivri (Viatris) [MYL-1401O]**<br>1. HER2-positive metastatic breast cancer (MBC)<br>2. HER2-positive early breast cancer (EBC)<br>3. HER2-positive metastatic adenocarcinoma of the stomach or gastroesophageal junction	
6			**Zercepac (Accord) [HLX02]**<br>1. HER2-positive metastatic breast cancer (MBC)<br>2. HER2-positive early breast cancer (EBC)<br>3. HER2-positive metastatic adenocarcinoma of the stomach or gastroesophageal junction	

Red font indicates that the four regulators (NMPA, FDA, EMA and PMDA) have approved the originator's indication, but the biosimilar has not yet been approved for that indication.
Abbreviations: FDA, US Food and Drug Administration; EMA, European Medicines Agency; PMDA, Japanese Pharmaceuticals and Medical Devices Agency; NMPA, National Medical Products Administration.

© 2023 Luo X et al. *JAMA Network Open.*

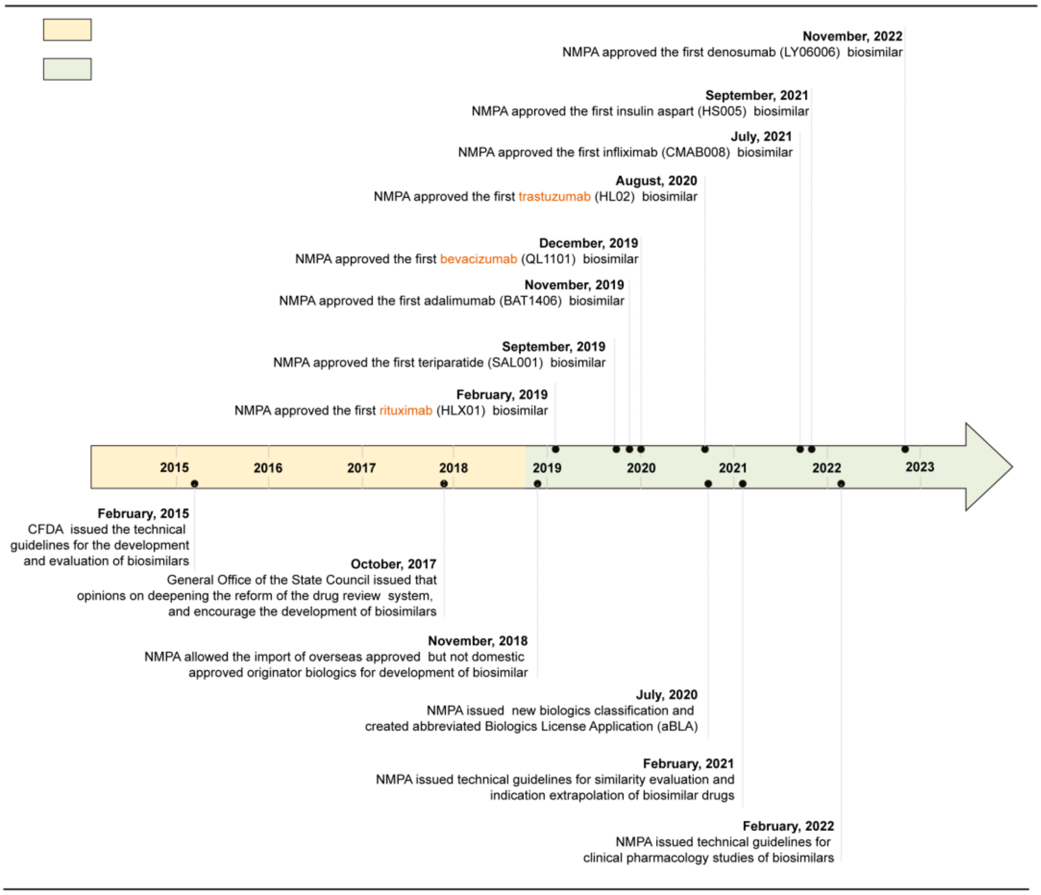

eFigure 1. The development history of biosimilars in China.
CFDA: China Food and Drug Administration. NMPA: National Medical Products Administration (Name changed to NMPA in September, 2018).

© 2023 Luo X et al. *JAMA Network Open.*

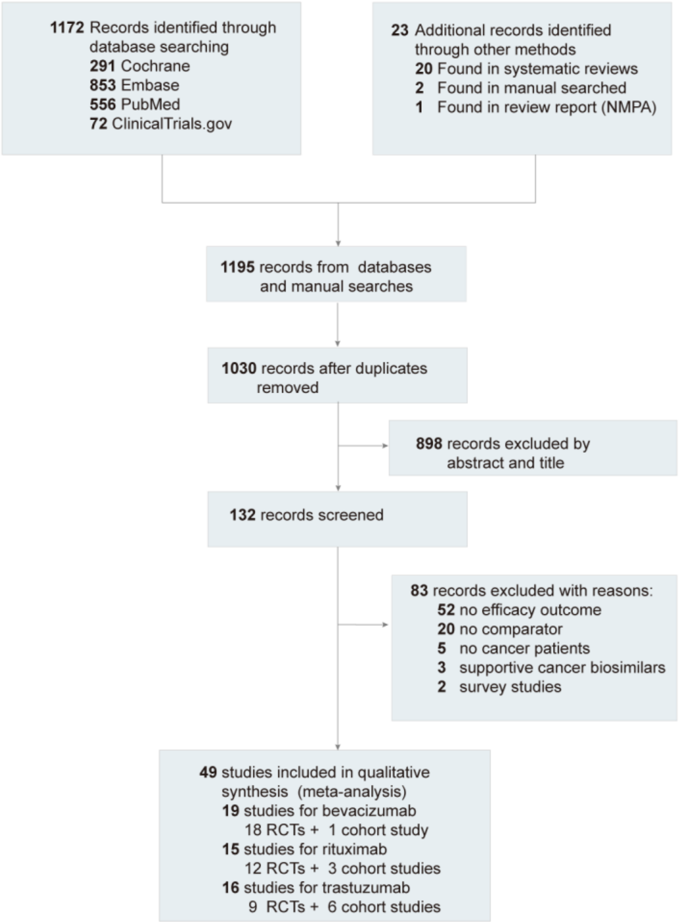

eFigure 2. Flowchart of included studies for the systematic review and meta-analysis.
Abbreviations: NMPA, National Medical Products Administration; RCT, randomized clinical trial.

Study	Biosimilar	Originator	Biosimilar Case, No.	Biosimilar Patients, No.	Originator Case, No.	Originator Patients, No.	RR (95%CI)	Weight, %
Kim et al, 2021	BI695502	Bevacizumab	181	335	207	328	0.86 (0.75; 0.97)	9.9
Trukhin et al, 2021	MB02	Bevacizumab	127	315	139	312	0.90 (0.75; 1.09)	4.9
Shi et al, 2021	LY01008	Bevacizumab	142	293	157	296	0.91 (0.78; 1.07)	6.4
Chu et al, 2021	QL1101	Bevacizumab	142	269	151	266	0.93 (0.80; 1.08)	6.9
Chen et al, 2022	BAT1706	Bevacizumab	145	325	156	326	0.93 (0.79; 1.10)	6.0
Socinski et al, 2021	MYL−14020	Bevacizumab	140	337	144	324	0.93 (0.78; 1.11)	5.3
Thatcher et al, 2019	ABP215	Bevacizumab	128	328	131	314	0.94 (0.77; 1.13)	4.6
Yang et al, 2019	IBI305	Bevacizumab	98	221	102	220	0.96 (0.78; 1.17)	3.9
Syrigos et al, 2021	FKB238	Bevacizumab	188	364	197	367	0.96 (0.84; 1.10)	8.7
Lu et al, 2023	TAB 008	Bevacizumab	155	277	151	271	1.00 (0.87; 1.17)	7.4
Verschraegen et al, 2022	CT−P16	Bevacizumab	145	342	146	347	1.01 (0.85; 1.20)	5.4
Reinmuth et al, 2019	PF−06439535	Bevacizumab	162	358	161	361	1.01 (0.86; 1.19)	6.2
Stroyakovskiy et al, 2022	BCD−021	Bevacizumab	71	205	46	136	1.02 (0.76; 1.38)	1.8
Hengrui, 2022	BP102	Bevacizumab	180	262	169	255	1.04 (0.92; 1.17)	11.4
Reck et al, 2019	SB8	Bevacizumab	181	379	164	383	1.12 (0.95; 1.30)	6.7
Wan et al, 2021	MIL60	Bevacizumab	123	253	110	255	1.13 (0.93; 1.36)	4.6
**Random effects model (DL)**				**4863**		**4761**	**0.97 (0.93; 1.01)**	**100.0**

Heterogeneity: $I^2 = 0\%$, $\tau^2 = 0$, $p = 0.60$

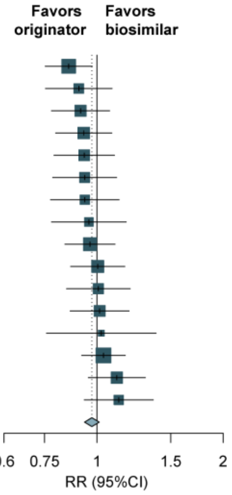

eFigure 3. Meta-analysis of the primary efficacy end points between bevacizumab biosimilars and the originator derived from RCTs.

Abbreviations: RR, risk ratio. DL, dersimonian and laird method. CI, confidence interval.

© 2023 Luo X et al. *JAMA Network Open.*

Study	Biosimilar	Originator	Biosimilar Case, No.	Biosimilar Patients, No.	Originator Case, No.	Originator Patients, No.	RR (95%CI)	Weight, %
FL, ORR								
Archigen, 2020	SAIT101	Rituximab	104	157	112	158	0.93 (0.80; 1.09)	6.3
Jurczak et al, 2017	GP2013	Rituximab	271	311	274	313	1.00 (0.94; 1.06)	14.9
Ogura et al, 2018	CT−P10	Rituximab	108	130	104	128	1.02 (0.91; 1.15)	8.9
Kim et al, 2017	CT−P10	Rituximab	64	66	63	68	1.05 (0.97; 1.13)	12.4
Kaplanov et al, 2014	BCD−020	Rituximab	18	46	17	46	1.06 (0.63; 1.78)	0.7
Sharman et al, 2020	PF−05280586	Rituximab	148	196	140	198	1.07 (0.95; 1.20)	8.4
Niederwieser et al, 2020	ABP 798	Rituximab	96	123	87	124	1.11 (0.96; 1.29)	6.4
Random effects model (DL)				1029		1035	1.02 (0.98; 1.06)	58.0
Heterogeneity: $I^2 = 0\%$, $p = 0.64$								
DLBCL, ORR								
Song et al, 2021	IBI301	Rituximab	186	207	196	209	0.96 (0.90; 1.01)	15.2
Shi et al, 2020	HLX01	Rituximab	177	188	180	194	1.01 (0.96; 1.07)	15.7
Candelaria et al, 2019	RTXM83	Rituximab	102	122	71	117	1.38 (1.17; 1.63)	5.5
Random effects model (DL)				517		520	1.07 (0.93; 1.24)	36.4
Heterogeneity: $I^2 = 91\%$, $p < 0.01$								
CLL, ORR								
Toogeh et al, 2018	Zytux	Rituximab	22	25	23	26	0.99 (0.81; 1.22)	4.1
Indolent NHL, ORR								
Poddunaya et al, 2020	BCD−020	Rituximab	38	85	31	74	1.07 (0.75; 1.53)	1.5
Random effects model (DL)				1656		1655	1.03 (0.98; 1.08)	100.0
Heterogeneity: $I^2 = 52\%$, $p = 0.02$								

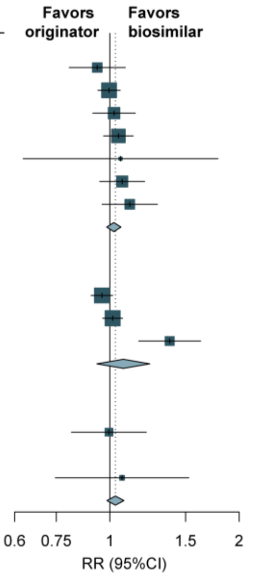

eFigure 4. Meta-analysis of the primary efficacy end points between rituximab biosimilars and the originator stratified by cancer type derived from RCTs.

Abbreviations: RR, risk ratio. DL, dersimonian and laird method. CI, confidence interval.

© 2023 Luo X et al. *JAMA Network Open.*

Study	Biosimilar	Originiator	Biosimilar Case, No.	Biosimilar Patients, No.	Originator Case, No.	Originator Patients, No.	RR (95%CI)	Weight, %
Early BC, pCR								
Pivot et al, 2022	HD201	Trastuzumab	107	238	115	236	0.92 (0.76; 1.12)	9.5
Stebbing et al, 2017	CT−P6	Trastuzumab	116	248	129	256	0.93 (0.78; 1.11)	10.3
Von Minckwitz et al, 2018	ABP 980	Trastuzumab	172	358	137	338	1.19 (1.00; 1.40)	11.2
Pivot et al, 2018	SB3	Trastuzumab	208	402	167	398	1.23 (1.06; 1.43)	12.7
Random effects model				1246		1228	1.07 (0.91; 1.24)	43.7
Heterogeneity: $I^2 = 68\%$ $P = 0.02$								
Metastatic BC, ORR								
Pegram et al, 2019	PF−05280014	Trastuzumab	220	352	236	355	0.94 (0.84; 1.05)	16.6
Xu et al, 2021	HLX02	Trastuzumab	231	324	232	325	1.00 (0.91; 1.10)	17.9
Rugo et al, 2017	MYL−14010	Trastuzumab	160	230	146	228	1.09 (0.95; 1.24)	14.5
Reza et al, 2022	TA4415V	Trastuzumab	18	48	15	44	1.10 (0.63; 1.91)	1.7
Alexeev et al, 2020	BCD−022	Trastuzumab	56	113	48	110	1.14 (0.86; 1.51)	5.5
Random effects model				1067		1062	1.01 (0.95; 1.07)	56.3
Heterogeneity: $I^2 = 0\%$, $P = 0.45$								
Random effects model				2313		2290	1.04 (0.97; 1.12)	100.0%
Heterogeneity: $I^2 = 47\%$, $P = 0.05$								

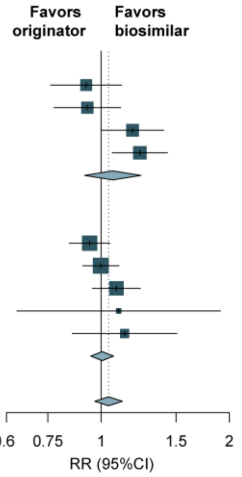

eFigure 5. Meta-analysis of the primary efficacy end points between trastuzumab biosimilars and the originator stratified by cancer type derived from RCTs.

Abbreviations: RR, risk ratio. DL, dersimonian and laird method. CI, confidence interval.

© 2023 Luo X et al. *JAMA Network Open.*

Study	RR	95%CI	*P* value	Tau²	Tau	*I*²
Omitting Stroyakovskiy et al, 2022	0.97	[0.93; 1.01]	0.10	0	0	0%
Omitting Thatcher et al, 2019	0.97	[0.93; 1.01]	0.14	0	0	0%
Omitting Reinmuth et al, 2019	0.97	[0.93; 1.01]	0.09	0	0	0%
Omitting Reck et al, 2019	0.96	[0.92; 1.00]	0.05	0	0	0%
Omitting Yang et al, 2019	0.97	[0.93; 1.01]	0.12	0	0	0%
Omitting Trukhin et al, 2021	0.97	[0.93; 1.01]	0.16	0	0	0%
Omitting Shi et al, 2021	0.97	[0.93; 1.01]	0.17	0	0	0%
Omitting Chu et al, 2021	0.97	[0.93; 1.01]	0.16	0	0	0%
Omitting Hengrui, 2022	0.96	[0.92; 1.00]	0.06	0	0	0%
Omitting Wan et al, 2021	0.96	[0.92; 1.00]	0.06	0	0	0%
Omitting Syrigos et al, 2021	0.97	[0.93; 1.01]	0.13	0	0	0%
Omitting Kim et al, 2021	0.98	[0.94; 1.02]	0.36	0	0	0%
Omitting Socinski et al, 2021	0.97	[0.93; 1.01]	0.14	0	0	0%
Omitting Chen et al, 2022	0.97	[0.93; 1.01]	0.15	0	0	0%
Omitting Lu et al, 2023	0.97	[0.93; 1.01]	0.10	0	0	0%
Omitting Verschraegen et al, 2022	0.97	[0.93; 1.01]	0.10	0	0	0%
Omitting Qin et al, 2021	0.97	[0.93; 1.01]	0.17	0	0	0%
**Random effects model (DL)**	**0.97**	**[0.93; 1.01]**	**0.11**	**0**	**0**	**0%**

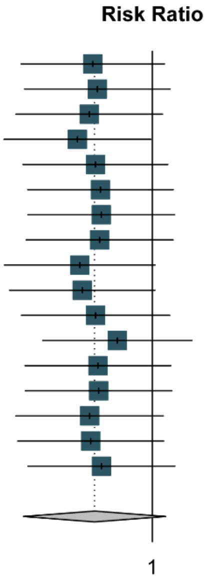

eFigure 6. Sensitivity analysis of primary efficacy end points for bevacizumab derived from RCTs.

Abbreviations: DL, dersimonian and laird method. CI, confidence interval.

© 2023 Luo X et al. *JAMA Network Open.*

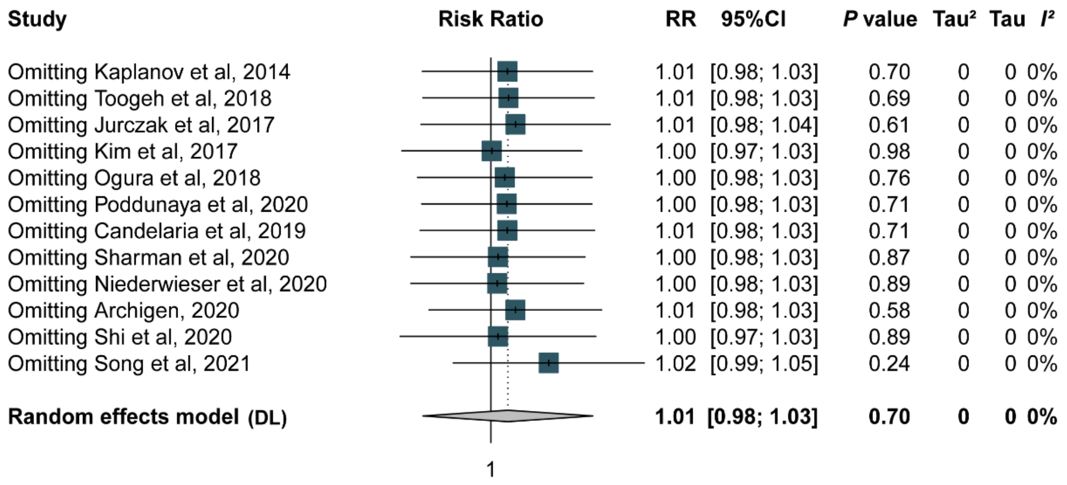

eFigure 7. Sensitivity analysis of primary efficacy end points for rituximab derived from RCTs.

Abbreviations: DL, dersimonian and laird method. CI, confidence interval.

© 2023 Luo X et al. *JAMA Network Open.*

Study	RR	95%CI	*P* value	Tau²	Tau	*I*²
Omitting Stebbing et al, 2017	1.06	[0.97; 1.14]	0.19	0.0060	0.0773	50%
Omitting Von Minckwitz et al, 2018	1.02	[0.95; 1.10]	0.54	0.0046	0.0676	43%
Omitting Pivot et al, 2018	1.01	[0.95; 1.08]	0.71	0.0017	0.0412	21%
Omitting Pivot et al, 2022	1.06	[0.97; 1.14]	0.19	0.0059	0.0771	50%
Omitting Nodehi et al, 2022	1.04	[0.96; 1.13]	0.32	0.0064	0.0801	54%
Omitting Rugo et al, 2017	1.04	[0.95; 1.13]	0.43	0.0072	0.0848	52%
Omitting Pegram et al, 2019	1.06	[0.98; 1.15]	0.13	0.0047	0.0689	40%
Omitting Alexeev et al, 2020	1.04	[0.96; 1.12]	0.38	0.0063	0.0792	53%
Omitting Xu et al, 2021	1.05	[0.96; 1.15]	0.27	0.0081	0.0898	52%
**Random effects model (DL)**	**1.04**	**[0.97; 1.12]**	**0.29**	**0.0056**	**0.0749**	**47%**

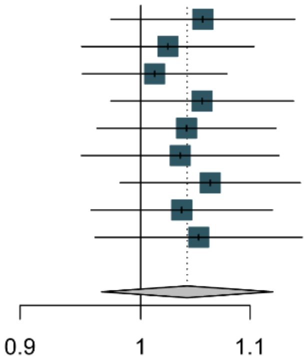

eFigure 8. Sensitivity analysis of primary efficacy end points for trastuzumab derived from RCTs.

Abbreviations: DL, dersimonian and laird method. CI, confidence interval.

© 2023 Luo X et al. *JAMA Network Open.*

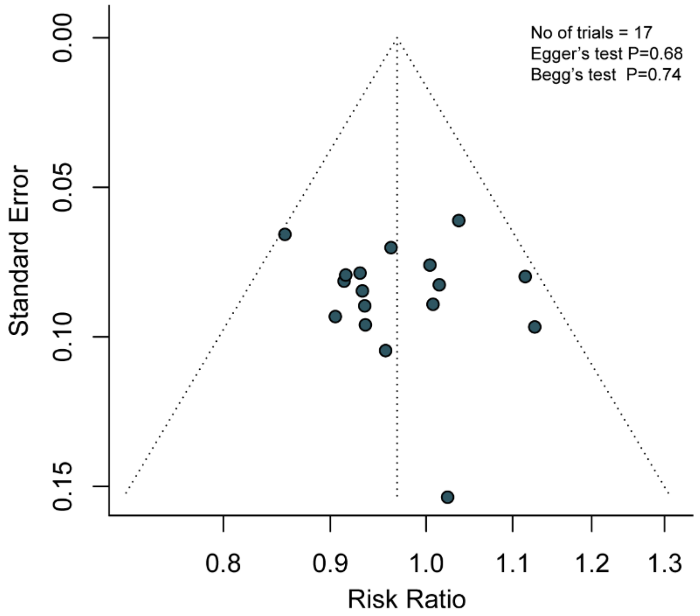

eFigure 9. Publication bias assessment of primary efficacy end points for bevacizumab derived from RCTs.

Abbreviations: RCTs: randomized controlled trials.

© 2023 Luo X et al. *JAMA Network Open.*

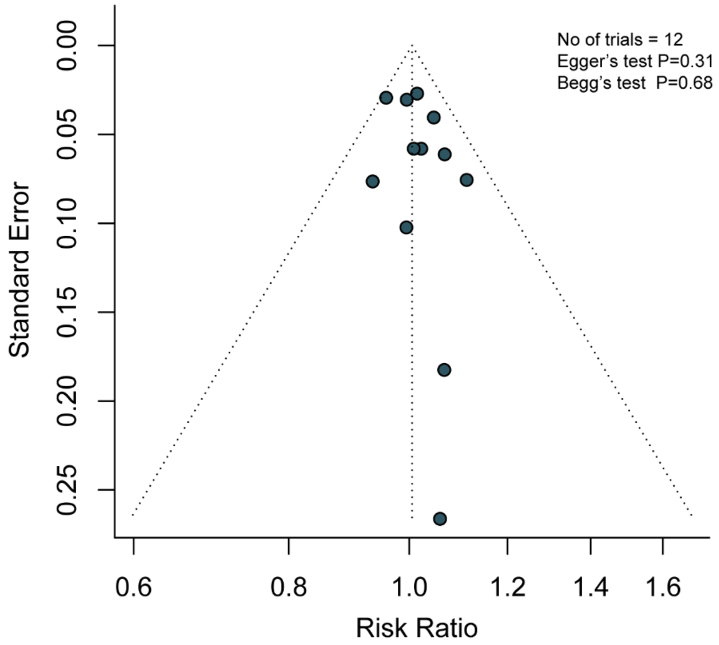

eFigure 10. Publication bias assessment of primary efficacy end points for rituximab derived from RCTs.

Abbreviations: RCTs: randomized controlled trials.

© 2023 Luo X et al. *JAMA Network Open.*

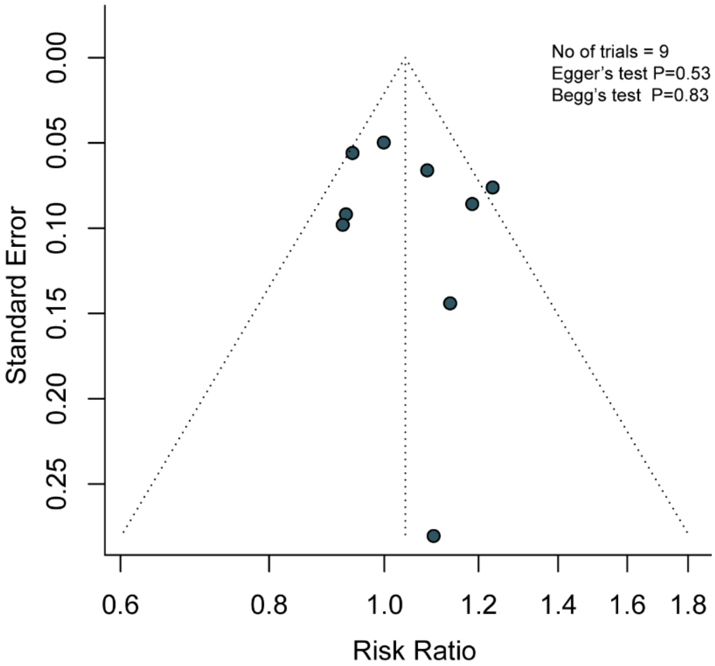

eFigure 11. Publication bias assessment of primary efficacy end points for trastuzumab derived from RCTs.

Abbreviations: RCTs: randomized controlled trials.

© 2023 Luo X et al. *JAMA Network Open.*

eReferences

eAppendix 3

1. Luo X, Du X, Huang L, et al. The price, efficacy, and safety of within-class targeted anticancer medicines between domestic and imported drugs in China: a comparative analysis. The Lancet regional health Western Pacific. 2023;32:100670.
2. Rui M, Li H. Cost-effectiveness of Osimertinib vs Docetaxel-bevacizumab in Third-line Treatment in EGFR T790M Resistance Mutation Advanced Non-Small Cell Lung Cancer in China. Clinical therapeutics. 2020;42(11):2159-2170.e2156.
3. Pharnexcloud. National hospital sales volume. Accessed August, 2023.https://pharma.bcpmdata.com/.

1. Stroyakovskiy DL, Fadeeva NV, Matrosova MP, et al. Randomized double-blind clinical trial comparing safety and efficacy of the biosimilar BCD-021 with reference bevacizumab. *BMC cancer.* 2022;22(1):129.
2. Thatcher N, Goldschmidt JH, Thomas M, et al. Efficacy and Safety of the Biosimilar ABP 215 Compared with Bevacizumab in Patients with Advanced Nonsquamous Non-small Cell Lung Cancer (MAPLE): A Randomized, Double-blind, Phase III Study. *Clinical cancer research : an official journal of the American Association for Cancer Research.* 2019;25(7):2088-2095.
3. Reinmuth N, Bryl M, Bondarenko I, et al. PF-06439535 (a Bevacizumab Biosimil ar) Compared with Reference Bevacizumab (Avastin(®)), Both Plus Paclitaxel an d Carboplatin, as First-Line Treatment for Advanced Non-Squamous Non-Small-Cell Lung Cancer: A Randomized, Double-Blind Study. *BioDrugs : clinical immun otherapeutics, biopharmaceuticals and gene therapy.* 2019;33(5):555-570.
4. Reck M, Luft A, Bondarenko I, et al. A phase III, randomized, double-blind, multicenter study to compare the efficacy, safety, pharmacokinetics, and immunogenicity between SB8 (proposed bevacizumab biosimilar) and reference bevacizumab in patients with metastatic or recurrent nonsquamous non-small cell lung cancer. *Lung cancer.* 2020;146:12-18.
5. Yang Y, Wu B, Huang L, et al. Biosimilar candidate IBI305 plus paclitaxel/carboplatin for the treatment of non-squamous non-small cell lung cancer. *Translational lung cancer research.* 2019;8(6):989-999.
6. Trukhin D, Poddubskaya E, Andric Z, et al. Efficacy, Safety and Immunogenicity of MB02 (Bevacizumab Biosimilar) versus Reference Bevacizumab in Advanced Non-Small Cell Lung Cancer: A Randomized, Double-Blind, Phase III Study (STELLA). *Br J Clin Pharmacol*. 2022; 88(3): 1063- 1073.
7. Rezvani H, Mortazavizadeh SM, Allahyari A, et al. Efficacy and Safety of Proposed Bevacizumab Biosimilar BE1040V in Patients With Metastatic Colorectal Cancer: A Phase III, Randomized, Double-blind, Noninferiority Clinical Trial. *Clinical therapeutics.* 2020;42(5):848-859.
8. Qin S, Li J, Bai Y, et al. Efficacy, Safety, and Immunogenicity of HLX04 Versus Reference Bevacizumab in Combination with XELOX or mFOLFOX6 as First-Line Treatment for Metastatic Colorectal Cancer: Results of a Randomized, Double-Blind Phase III Study. *BioDrugs.* 2021 Jul;35(4):445-458.
9. Shi Y, Lei K, Jia Y, et al. Bevacizumab biosimilar LY01008 compared with bevacizumab (Avastin) as first-line treatment for Chinese patients with unresectable, metastatic, or recurrent non-squamous non-small-cell lung cancer: A multicenter, randomized, double-blinded, phase III trial. *Cancer Commun*. 2021; 41: 889– 903.
10. Chu T, Lu J, Bi M, et al. Equivalent efficacy study of QL1101 and bevacizumab on untreated advanced non-squamous non-small cell lung cancer patients: a phase 3 randomized, double-blind clinical trial. *Cancer Biol Med.* 2021;18(3):816-824.
11. China National Medical Products Administration. The review reports of bevacizumab biosimilar. Accessed August 4, 2023. https://file1.dxycdn.com/2022/0922/070/7198507456499203753-117.pdf

© 2023 Luo X et al. *JAMA Network Open.*

12. Wan R, Dong X, Chen Q, et al. Efficacy and safety of MIL60 compared with bevacizumab in advanced or recurrent non-squamous non-small cell lung cancer: a phase 3 randomized, double-blind study. *EClinicalMedicine.* 2021;42:101187.
13. Syrigos K, Abert I, Andric Z, et al. Efficacy and Safety of Bevacizumab Biosimilar FKB238 Versus Originator Bevacizumab: Results from AVANA, a Phase III Trial in Patients with Non-Squamous Non-Small-Cell Lung Cancer (non-sq-NSCLC). *BioDrugs* .2021;35(4):417-428.
14. Kim ES, Balser S, Rohr KB, Lohmann R, Liedert B, Schliephake D. Phase 3 Trial of BI 695502 Plus Chemotherapy Versus Bevacizumab Reference Product Plus Chemotherapy in Patients With Advanced Nonsquamous NSCLC. *JTO clinical and research reports.* 2022;3(1):100248.
15. Socinski MA, Waller CF, Idris T, et al. Phase III double-blind study comparing th e efficacy and safety of proposed biosimilar MYL-1402O and reference bevacizu mab in stage IV non-small-cell lung cancer. *Ther Adv Med Oncol.* 2021;13:1758 8359211045845.
16. Chen L, Trukhin D, Kolesnik O, et al. Clinical efficacy and safety of the BAT1706 (proposed bevacizumab biosimilar) compared with reference bevacizumab in patients with advanced nonsquamous NSCLC: A randomized, double-blind, phase III study. *Journal of Clinical Oncology.* 2022 40:16_suppl, 9041-9041.
17. Lu S, Qin S, Zhou Z, et al. Bevacizumab biosimilar candidate TAB008 compared to Avastin(®) in patients with locally advanced, metastatic EGFR wild-type non-squamous non-small cell lung cancer: a randomized, double-blind, multicenter study. *Journal of cancer research and clinical oncology.* 2023;149(9):5907-5914.
18. Verschraegen C, Andric Z, Moiseenko F, et al. Candidate Bevacizumab Biosimilar CT-P16 versus European Union Reference Bevacizumab in Patients with Metastatic or Recurrent Non-Small Cell Lung Cancer: A Randomized Controlled Trial. *BioDrugs.* 2022;36(6):749-760.
19. Kaplanov K, Zaritskiy A, Alexeev S, et al. Key Results of International Randomized Open-Label Clinical Study of BCD-020 (rituximab biosimilar candidate) in Patients with B-Cell Non-Hodgkin’s Lymphoma. *Blood.* 2014;124(21):5467-5467.
20. Toogeh G, Faranoush M, Razavi SM, et al. A Double-Blind, Randomized Comparison Study between Zytux™ vs MabThera® in Treatment of CLL with FCR Regimen: Non-Inferiority Clinical Trial. *Int J Hematol Oncol Stem Cell Res.* 2018 Apr 1;12(2):84-91.
21. Jurczak W, Moreira I, Kanakasetty GB, et al. Rituximab biosimilar and reference rituximab in patients with previously untreated advanced follicular lymphoma (ASSIST-FL): primary results from a confirmatory phase 3, double-blind, randomised, controlled study. *The Lancet Haematology.* 2017;4(8):e350-e361.
22. Kim WS, Buske C, Ogura M, et al. Efficacy, pharmacokinetics, and safety of the biosimilar CT-P10 compared with rituximab in patients with previously untreated advanced-stage follicular lymphoma: a randomised, double-blind, parallel-group, non-inferiority phase 3 trial. *The Lancet Haematology.* 2017;4(8):e362-e373.
23. Ogura M, Sancho JM, Cho SG, et al. Efficacy, pharmacokinetics, and safety of the biosimilar CT-P10 in comparison with rituximab in patients with previously untreated low-tumour-burden follicular lymphoma: a randomised, double-blind, parallel-group, phase 3 trial. *The Lancet Haematology.* 2018;5(11):e543-e553.
24. Poddubnaya IV, Alekseev SM, Kaplanov KD, et al. Proposed rituximab biosimilar BCD-020 versus reference rituximab for treatment of patients with indolent non-Hodgkin lymphomas: An international multicenter randomized trial. *Hematological oncology.* 2020;38(1):67-73.
25. Candelaria M, González DE, Delamain MT, et al. Rituximab biosimilar RTXM83 versus reference rituximab in combination with CHOP as first-line treatment for diffuse large B-cell lymphoma: a randomized, double-blind study. *Leukemia & lymphoma.* 2019;60(14):3375-3385.
26. Sharman JP, Liberati AM, Ishizawa K, et al. A Randomized, Double-Blind, Effica cy and Safety Study of PF-05280586 (a Rituximab Biosimilar) Compared with Ri tuximab Reference Product (MabThera(®)) in Subjects with Previously Untreated CD20-Positive, Low-Tumor-Burden Follicular Lymphoma (LTB-FL). *BioDrugs.* 202 0;34(2):171-181.
27. Niederwieser D, Hamm C, Cobb P, et al. Efficacy and Safety of ABP 798: Results from

the JASMINE Trial in Patients with Follicular Lymphoma in Comparison with Rituximab Reference Product. *Targeted oncology.* 2020;15(5):599-611.
28. Clinicaltrials.gov. A Randomized, Double-blind, Multi-center, Multi-national Trial to Evaluate the Efficacy, Safety, and Immunogenicity of SAIT101 Versus Rituximab as a First-line Immunotherapy Treatment in Patients With Low Tumor Burden Follicular Lymphoma. Accessed August 4, 2023. https://clinicaltrials.gov/study/NCT02809053?term=NCT02809053&rank=1.
29. Shi Y, Song Y, Qin Y, et al. A phase 3 study of rituximab biosimilar HLX01 in patients with diffuse large B-cell lymphoma. *Journal of hematology & oncology.* 2020;13(1):38.
30. Song Y, Zhou H, Zhang H, et al. Efficacy and Safety of the Biosimilar IBI301 Plus Standard CHOP (I-CHOP) in Comparison With Rituximab Plus CHOP (R-CHOP) in Patients with Previously Untreated Diffuse Large B-Cell Lymphoma (DLBCL): A Randomized, Double-Blind, Parallel-Group, Phase 3 Trial. *Advances in therapy.* 2021;38(4):1889-1903.
31. Stebbing J, Baranau Y, Baryash V, et al. CT-P6 compared with reference trastuzumab for HER2-positive breast cancer: a randomised, double-blind, active-controlled, phase 3 equivalence trial. *The Lancet Oncology.* 2017;18(7):917-928.
32. Rugo HS, Barve A, Waller CF, et al. Effect of a Proposed Trastuzumab Biosimilar Compared With Trastuzumab on Overall Response Rate in Patients With ERBB2 (HER2)-Positive Metastatic Breast Cancer: A Randomized Clinical Trial. *JAMA.* 2017;317(1):37-47.
33. von Minckwitz G, Colleoni M, Kolberg HC, et al. Efficacy and safety of ABP 980 compared with reference trastuzumab in women with HER2-positive early breast cancer (LILAC study): a randomised, double-blind, phase 3 trial. *The Lancet Oncology.* 2018;19(7):987-998.
34. Pivot X, Bondarenko I, Nowecki Z, et al. Phase III, Randomized, Double-Blind Study Comparing the Efficacy, Safety, and Immunogenicity of SB3 (Trastuzumab Biosimilar) and Reference Trastuzumab in Patients Treated With Neoadjuvant Therapy for Human Epidermal Growth Factor Receptor 2-Positive Early Breast Cancer. *Journal of clinical oncology.* 2018;36(10):968-974.
35. Pegram MD, Bondarenko I, Zorzetto MMC, et al. PF-05280014 (a trastuzumab biosimilar) plus paclitaxel compared with reference trastuzumab plus paclitaxel for HER2-positive metastatic breast cancer: a randomised, double-blind study. *British journal of cancer.* 2019;120(2):172-182.
36. Pivot X, Georgievich MA, Shamrai V, et al. Efficacy of HD201 vs Referent Trastuzumab in Patients With ERBB2-Positive Breast Cancer Treated in the Neoadjuvant Setting: A Multicenter Phase 3 Randomized Clinical Trial. *JAMA oncology.* 2022;8(5):698-705.
37. Alexeev SM, Khorinko AV, Mukhametshina GZ, et al. Randomized double-blind clinical trial comparing safety and efficacy of the biosimilar BCD-022 with reference trastuzumab. *BMC cancer.* 2020;20(1):783.
38. Xu B, Zhang Q, Sun T, et al. Efficacy, Safety, and Immunogenicity of HLX02 Compared with Reference Trastuzumab in Patients with Recurrent or Metastatic HER2-Positive Breast Cancer: A Randomized Phase III Equivalence Trial. *BioDrugs.* 2021;35(3):337-350.
39. Nodehi RS, Kalantari B, Raafat J, et al. A randomized, double-blind, phase III, non-inferiority clinical trial comparing the efficacy and safety of TA4415V (a proposed Trastuzumab biosimilar) and Herceptin (Trastuzumab reference product) in HER2-positive early-stage breast Cancer patients. *BMC pharmacology & toxicology.* 2022;23(1):57.
40. Roy PS, John S, Karankal S, et al. Comparison of the efficacy and safety of Rituximab (Mabthera™) and its biosimilar (Reditux™) in diffuse large B-cell lymphoma patients treated with chemo-immunotherapy: A retrospective analysis. *Indian journal of medical and paediatric oncology.* 2013;34(4):292-298.
41. Bankar A, Korula A, Abraham A, et al. Comparison of the Efficacy of Innovator Rituximab and its Biosimilars in Diffuse Large B Cell Lymphoma Patients: A Retrospective Analysis. *Indian journal of hematology & blood transfusion.* 2020;36(1):71-77.
42. Deng W, Yang S, Yang C, et al. Rituximab biosimilar HLX01 versus reference rituximab in the treatment of diffuse large B-cell lymphoma: Real-world clinical

experience. *Journal of oncology pharmacy practice.* 2022:10781552221110470.

43. Zhao Z, Zhao L, Xia G, et al. Efficacy and safety of bevacizumab biosimilar compared with reference bevacizumab in locally advanced and advanced non-small cell lung cancer patients: A retrospective study. *Frontiers in oncology.* 2022;12:1036906.
44. Bae SJ, Kim JH, Ahn SG, et al. Real-World Clinical Outcomes of Biosimilar Trastuzumab (CT-P6) in HER2-Positive Early-Stage and Metastatic Breast Cancer. *Frontiers in oncology.* 2021;11:689587.
45. Park JH, Yeo JH, Kim YS, et al. Efficacy and Safety of Trastuzumab Biosimilar (CT-P6) Compared With Reference Trastuzumab in Patients With HER2-positive Advanced Gastric Cancer: A Retrospective Analysis. *Am J Clin Oncol.* 2022;45(2):61-65.
46. Yang C, Khwaja R, Tang P, Nixon N, King K, Lupichuk S. A Review of Trastuzumab Biosimilars in Early Breast Cancer and Real World Outcomes of Neoadjuvant MYL-1401O versus Reference Trastuzumab. *Curr Oncol*. 2022;29(6):4224-4234.
47. Bernat-Peguera A, Trigueros M, Ferrando-Díez A, et al. Efficacy of CT-P6 (trastuzumab biosimilar) versus reference trastuzumab in combination with pertuzumab in HER2-positive early-stage breast cancer: Preclinical and real-life clinical data. *Breast.* 2022;62:1-9.
48. Eser K, Sezer E, Erçolak V, İnal A. Evaluation of biosimilar trastuzumab MYL-1401O in HER2-positive breast cancer. *The American journal of managed care.* 2023;29(2):e36-e42.
49. Liu Z, Guan Y, Yao Y, et al. Effectiveness and Safety of Zercepac and Reference Trastuzumab in the Neoadjuvant Setting for Early-Stage Breast Cancer: A Retrospective Cohort Study. *Journal of oncology.* 2022;2022:9998114.
