# Supplementary material for: Clinical Benefit, Price, and Uptake for Cancer Biosimilars vs Reference Drugs in China: A Systematic Review and Meta-Analysis
Source: JAMA Netw Open. 2023 Oct 12;6(10):e2337348. doi: 10.1001/jamanetworkopen.2023.37348 (PMC10570888; doi:10.1001/jamanetworkopen.2023.37348)
Supplement: Supplement 2. — Data Sharing Statement [file jamanetwopen-e2337348-s002.pdf]

Data Sharing Statement

Luo. Clinical Benefit, Price, and Uptake for Cancer Biosimilars vs Reference Drugs in China. *JAMA Netw Open*. Published October 12, 2023. doi:10.1001/jamanetworkopen.2023.37348

Data
Data available: No
